# Mechanistic Investigation into Copper(I) Hydride Catalyzed Formic Acid Dehydrogenation

**DOI:** 10.1021/acscatal.4c05008

**Published:** 2024-10-07

**Authors:** Roel L. M. Bienenmann, Anne Olarte Loyo, Martin Lutz, Daniël L. J. Broere

**Affiliations:** † Organic Chemistry and Catalysis, Institute for Sustainable and Circular Chemistry, Faculty of Science, Utrecht University, Universiteitsweg 99, 3584 CG Utrecht, The Netherlands; ‡ Structural Biochemistry, Bijvoet Centre for Biomolecular Research, Faculty of Science, Utrecht University, Universiteitsweg 99, 3584 CG Utrecht, The Netherlands

**Keywords:** formic acid, dehydrogenation, dinuclear, homogeneous catalysis, copper, mechanistic investigation

## Abstract

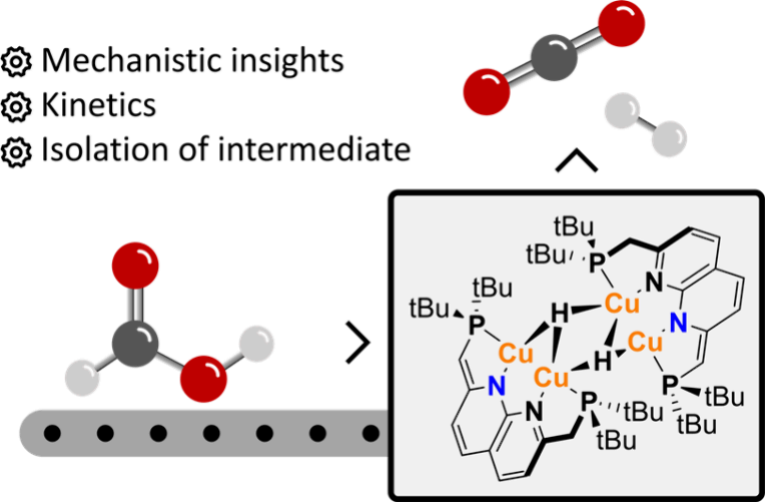

Copper(I) hydride complexes are typically known to react with CO_2_ to form their corresponding copper formate counterparts. However, recently it has been observed that some multinuclear copper hydrides can feature the opposite reactivity and catalyze the dehydrogenation of formic acid. Here we report the use of a multinuclear PNNP copper hydride complex as an active (pre)catalyst for this reaction. Mechanistic investigations provide insights into the catalyst resting state and the rate-determining step and identify an off-cycle species that is responsible for the unexpected substrate inhibition in this reaction.

## Introduction

**Figure 1:**
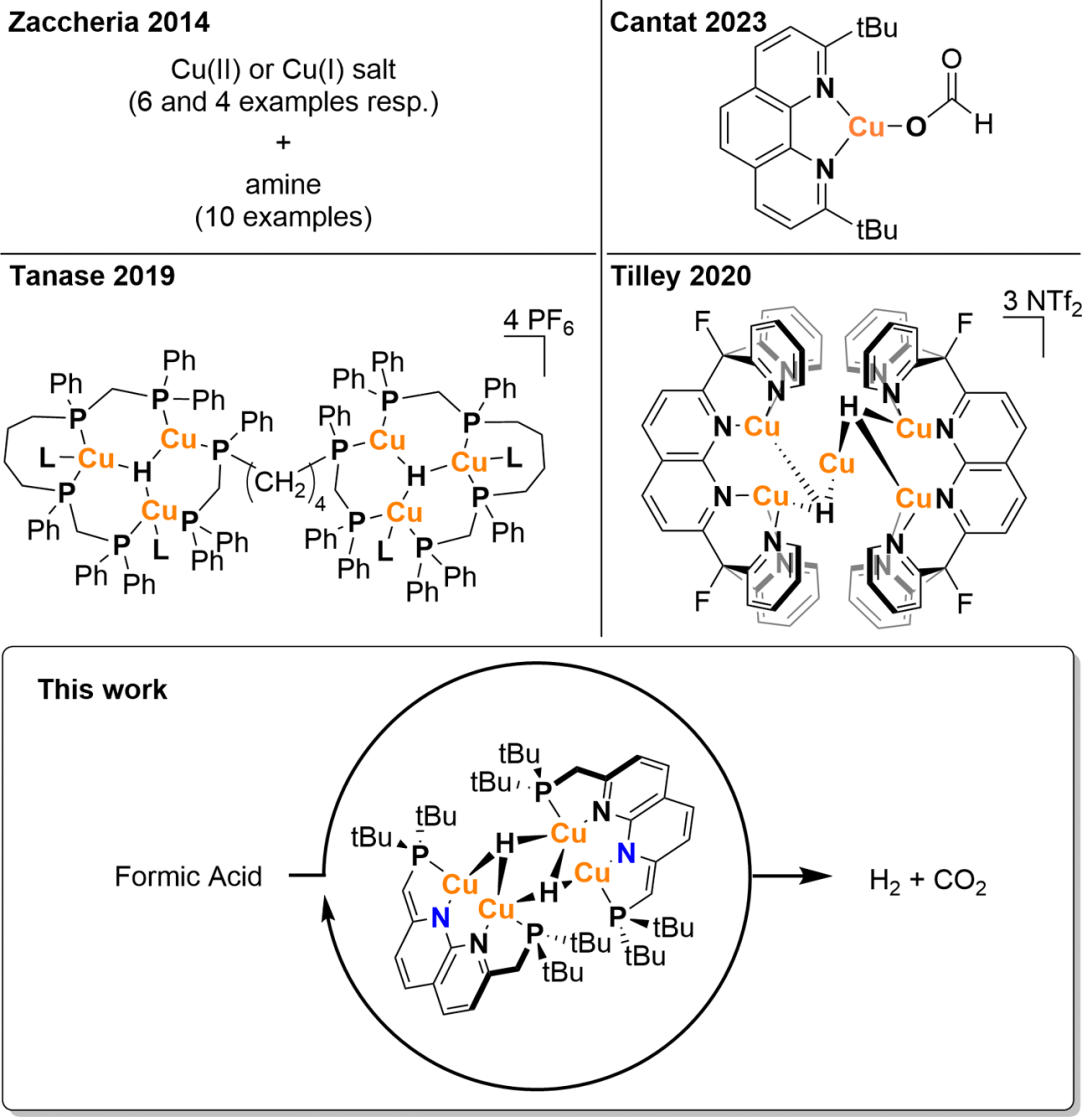
An overview of the copper-based homogeneous catalysts reported for the dehydrogenation of formic acid^11,13,14,16^ and the PNNP-based tetracopper dihydride^17^ catalyzed formic acid dehydrogenation described herein.

Both low-nuclearity copper(I) hydride complexes and copper clusters are well-known to react with CO_2_ to form the corresponding formate complexes by hydride insertion.^1−10^ However, recently some examples of copper complexes which feature the opposite reactivity have been found. In these cases, either no reactivity of the copper hydride species is observed with CO_2_, or facile decarboxylation of the formate species to the corresponding copper hydride is found. For example, Tanase and co-workers reported a hexacopper dihydride complex (Figure 1) which reacts to the corresponding formate complex at 1 atm of CO_2_, but which also releases the CO_2_ again upon removal of the CO_2_ atmosphere.^11^ In addition, this complex is able to catalytically dehydrogenate formic acid (FA) selectively at mol % only 45 °C without additives (3.3 mol % Cu, TON 8.1 in 3 h). With triethylamine and tBuNC at 70 °C, this activity can be increased (0.13 mol % Cu, TON 720 in 3 h). The group of Tilley reported a pentanuclear copper dihydride complex (Figure 1) as well as a trinuclear one, both of which do not react with CO_2_ but are active for catalytic FA dehydrogenation at 80 °C (5 mol % Cu, TON 14 in 5 h^12^).^13^

Catalytic FA dehydrogenation with copper complexes is rare and only a handful of reports of this reactivity exist to date. Besides the two examples mentioned above, the group of Zaccheria reported the use of various simple copper(I) and (II) salts in combination with amine ligands to catalytically dehydrogenate FA (Figure 1).^14^ These reactions take place with high selectivity, but a high temperature (95 °C) is required and the conversion is low (0.9 mol % Cu, TON 72 in 22 h for CuI with triethylamine). In a mechanistic study, it was found that the rate-determining step (RDS) is the dissociation of an amine ligand from the mononuclear copper complexes, which opens up a free coordination site for β-hydride elimination.^15^ In this case, the high barrier for ligand dissociation necessitates the harsh reaction conditions. Recently, the group of Cantat reported catalytic FA dehydrogenation with a mononuclear Cu(I) complex supported by a phenanthroline-based ligand (Figure 1), which requires no additives to enable the reaction.^16^ Also in this case, high temperature (100 °C) and high catalyst loadings are required for the FA dehydrogenation reaction (5 mol % Cu, TON 5.4 in 3 h).

The higher reaction temperatures in these latter two cases with mononuclear complexes imply that the nuclearity of the complex is important for the conditions under which FA dehydrogenation is feasible. Additional stabilization of the hydride that is formed during the rate-determining C–H cleavage step has been proposed to play an important role.^11^ However, since there are only two examples of multinuclear copper(I) complexes for which this reactivity has been reported, it is unclear whether this trend extends to other multinuclear copper hydride complexes and what the design principles for copper-based FA dehydrogenation catalysts are.

In this work, we describe the use of our previously reported PNNP-based copper hydride complex **1** (Figure 1 bottom)^17^ for the catalytic dehydrogenation of FA. In addition to high activity relative to other copper systems and high selectivity under mild conditions, the system is well-behaved as clean reformation of the (pre)catalyst is observed after full conversion of FA. Through detailed kinetic studies, kinetic isotope effect (KIE) measurements, the isolation of catalytic intermediates, and the study of synthetic analogues we provide insight into the species involved in this catalytic reaction. Furthermore, we performed an extensive computational study which provides new insights into the mechanism copper catalyzed FA dehydrogenation.

## Formic Acid Dehydrogenation

**Figure 2:**
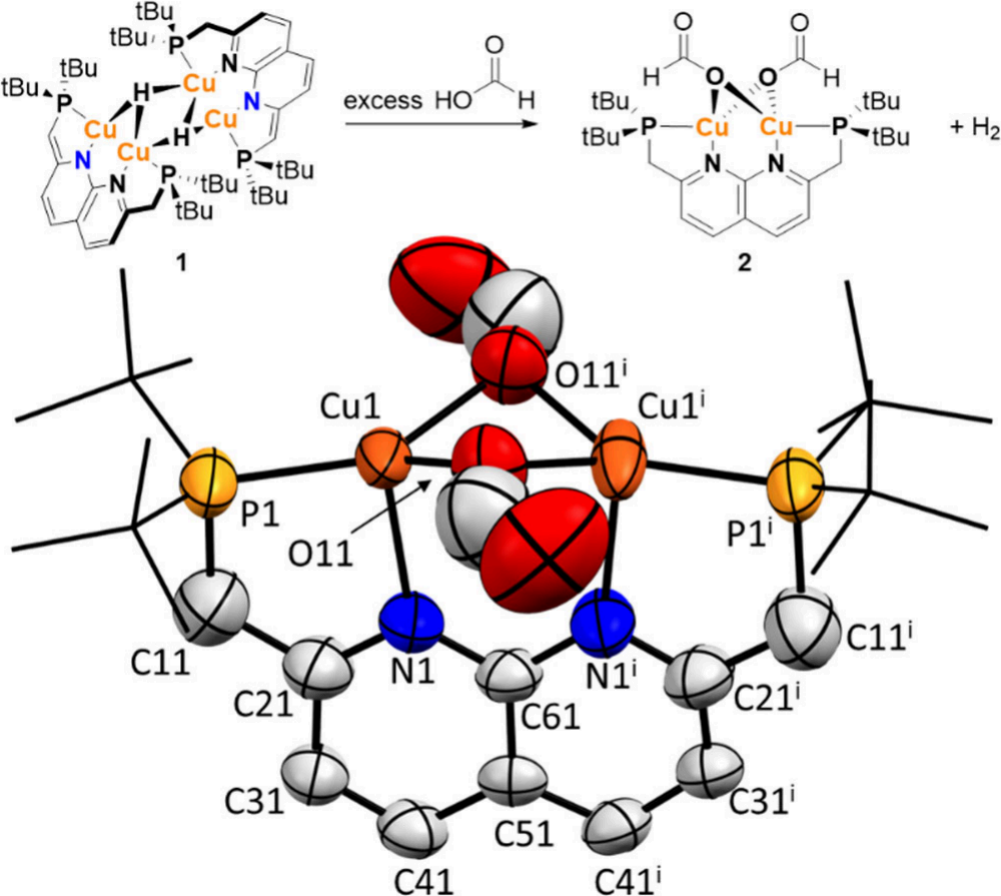
Synthesis and solid-state structure of **2**. Ellipsoids are shown at 50% probability. Only one disorder component is shown. Hydrogen atoms and the second independent molecule were removed for clarity. tBu-groups are shown in wireframe. Selected distances (Å): Cu1–Cu1^i^ 2.847(4), Cu1–O11 2.048(11), Cu1–O11^i^ 2.024(11), Cu1–P1 2.155(4), Cu1–N1 2.242(9), C11–C21 1.495(18). Selected distances for the second molecule of **2** in the asymmetric unit (Å): Cu3–Cu3^ii^ 2.819(5), Cu3–O13 2.081(10), Cu3–O13^ii^ 2.036(11), Cu3–P3 2.162(4), Cu3–N3 2.228(8), C13–C23 1.502(18). Symmetry codes i: -x, 1-y, z; ii: 1-x, 1-y, z.

When tetranuclear copper hydride complex **1** is exposed to a CO_2_ atmosphere, no reaction is observed, even after heating the solution at 80 °C. This indicated to us that the dehydrogenation of formic acid was potentially feasible with this complex.^13^ Reacting **1** with 10 equiv of FA at room temperature in THF leads to an immediate color change from red to yellow and the formation of an orange precipitate. ^1^H NMR analysis in THF shows the formation of H_2_ and full conversion of **1** into a new species (**2**). Complex **2**, features two aromatic doublets (8.27 and 7.57 ppm) for the naphthyridine backbone and one doublet (1.30 ppm) for the tBu-groups on the phosphines. Whereas **1** features unsymmetric partially dearomatized PNNP ligands, the observed resonances of **2** are indicative of a C_2V_ symmetrical complex with a fully aromatic naphthyridine backbone. The signal for the methylene linker is not visible in THF due to overlap with the solvent resonance. In addition, a very broad resonance at 11.13 ppm is observed for the FA OH group and a broadened resonance is observed between 7.93 and 8.43 ppm for the formate/FA C–H hydrogen. These resonances are indicative of the formation of a neutral bisformate complex **2** (Figure 2), which exchanges its formate ligands rapidly with free FA in solution, hence showing only one formate C–H resonance.

After heating the mixture to 50 °C for 1 h, an 82% decrease in the FA concentration is observed (Figure S22). Moreover, the C–H resonance of FA is shifted downfield, away from the position of this resonance in free FA. These observations support the proposed fast exchange of the formate ligands in **2** with free formic acid. The formation of both H_2_ gas and CO_2_ is observed by ^1^H NMR and ^13^C NMR analysis, respectively. In addition, no CO is observed in either the solution phase or headspace based on NMR and GC analysis. This shows that **1** is an active and selective (pre)catalyst for the catalytic FA dehydrogenation.

Interestingly, after heating for 3.5 h at 50 °C, the red color of the solution reappears and ^1^H NMR shows the presence of **1**. Further heating at 50 °C for 3 days leads to full conversion of formic acid and clean formation of complex **1**.^18^ It should be noted here that the formic acid dehydrogenation also proceeds at room temperature, but at a lower rate. The combination of the selectivity of the catalytic dehydrogenation reaction and the observation that the catalyst returns to its original form means that the reaction is well-behaved. Since copper hydride catalyzed FA dehydrogenation is a rare reaction, especially under mild conditions, we sought to better understand the mechanism of this reaction. This could help to better understand what features make **1** an active (pre)catalyst for FA dehydrogenation under mild conditions.

## Resting State of the Catalyst

Although complex **2** is formed during the FA dehydrogenation in THF, it is only sparingly soluble (1.7 mmol/L) in this solvent based on quantitative ^1^H NMR analysis. Hence, we attempted to generate **2** in a more polar solvent to aid its characterization. Although **1** is not soluble in MeCN, upon the addition of excess FA a yellow homogeneous solution forms. The ^1^H NMR resonance for the methylene linker is not obscured in MeCN and is observed as a doublet at 3.50 ppm. The general features that are observed in the NMR spectra, are similar to those described for the solution of **2** in THF, indicating that the same species is formed in both solvents. In MeCN, the formic acid dehydrogenation reaction also takes place, however, it is much slower than in THF at the same temperature (see Supporting Information). We hypothesize that the ability of MeCN to better stabilize charged species causes the reaction to be slower in this solvent (see below). In contrast to the reaction in THF, after depletion of formic acid in MeCN, the reaction does not return to its original color. Instead, complex **2** crystallizes out as orange-brown needles, on which we performed a single-crystal X-ray structure determination (Figure 2). The solid-state structure of **2** reveals a neutral bisformate complex consistent with the NMR analysis. Two independent molecules of **2** which are disordered on 2-fold rotation axes, respectively, are present in the asymmetric unit. In solution, **2** displays higher symmetry than is observed in the solid-state due to facile rotation around the C–O bonds of the formate ligands. An interesting observation is that the formate ligands bind in a terminal fashion rather than an η_2_-fashion in the solid-state, although it is likely that both binding modes are feasible in solution as is also supported by DFT calculations (see below). When the crystals of **2** are redissolved in THF, complex **1** is observed due to the facile conversion of **2** in this solvent. This further supports the assignment of **2** as the resting state that is also observed in THF. We reason that **2** crystallizes in MeCN because the decrease in FA concentration decreases the solubility of **2** in this solvent, leading to crystallization. Since the FA dehydrogenation reaction is fastest in THF and since **1** is formed after full conversion of the FA substrate in THF, this solvent was used for the further catalytic experiments.

**Scheme 1:**
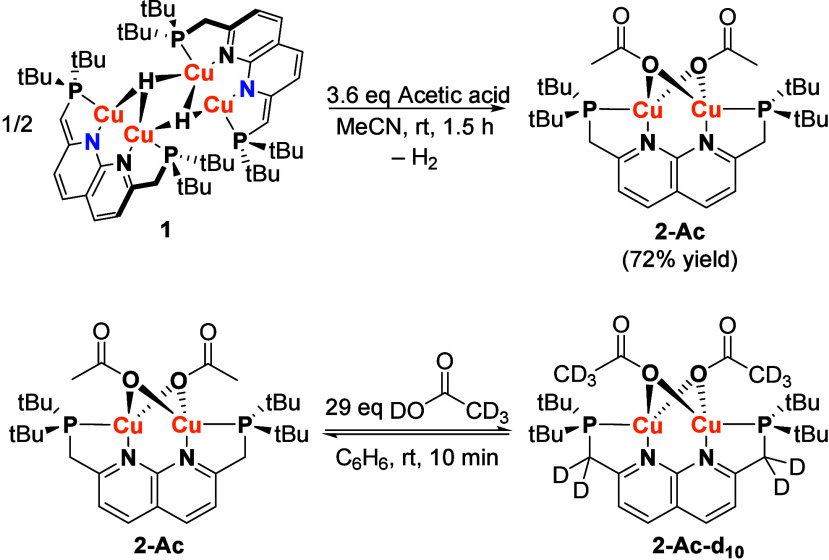
Synthesis of Complex **2-Ac** (Top) and the Reaction of **2-Ac** with Deuterated Acetic Acid (Bottom).

As discussed previously, only one formate peak is observed in ^1^H NMR for complex **2** in the presence of excess FA, which indicates a fast exchange of the formate ligand in solution. Since such an exchange is important to consider for the FA dehydrogenation mechanism, we probed whether this exchange proceeds through a metal–ligand cooperative pathway. However, since complex **2** in THF solution converts into complex **1** in the absence of FA, it is challenging to study its stoichiometric reactivity. Therefore, we synthesized an analogue of complex **2** which contains acetate ligands rather than formate ligands (**2-Ac**, Scheme 1), since this prevents the conversion to **1**. Complex **2-Ac** is readily synthesized by reacting **1** with acetic acid at room temperature. It is stable in solution and the NMR spectra also show a C_2V_ symmetrical complex with a fully aromatic naphthyridine backbone, for which the resonances are found at similar chemical shifts as those observed for **2**. The acetate peak of **2-Ac** at 2.34 ppm integrates to 6 protons, indicating that there are two acetate ligands present, analogous to what was observed for **2** in the solid-state structure. In the presence of excess acetic acid, the acetate resonance of **2-Ac** shifts upfield toward the position where free acetic acid is found. This indicates that a rapid exchange takes place analogous to what was observed for **2** and FA.

^1^H NMR analysis of a benzene solution containing complex **2-Ac** and 29 equiv of acetic acid-d_4_, shows the rapid exchange of the acetate ligands with acetic acid-d_4_ as expected (Scheme 1). However, also almost complete deuteration of the methylene linkers was observed in the first spectrum measured (after ∼10 min). This deuteration was confirmed by ^2^H NMR analysis of the mixture. Analogous experiments using the free PNNP ligand or PNNPCu_2_Cl_2_^17^ instead of **2-Ac** show no ligand deuteration. In addition, performing the FA dehydrogenation of FA-*d*_2_ with **1** also yields complex **1** with deuterated methyne and methylene linkers at the end of the reaction. This indicates that the mechanism of formate/acetate exchange involves metal–ligand cooperative elimination of formic/acetic acid with a proton that originates from a methylene linker, and that this reaction also takes place under catalytic conditions.

## Rate Law

**Figure 3:**
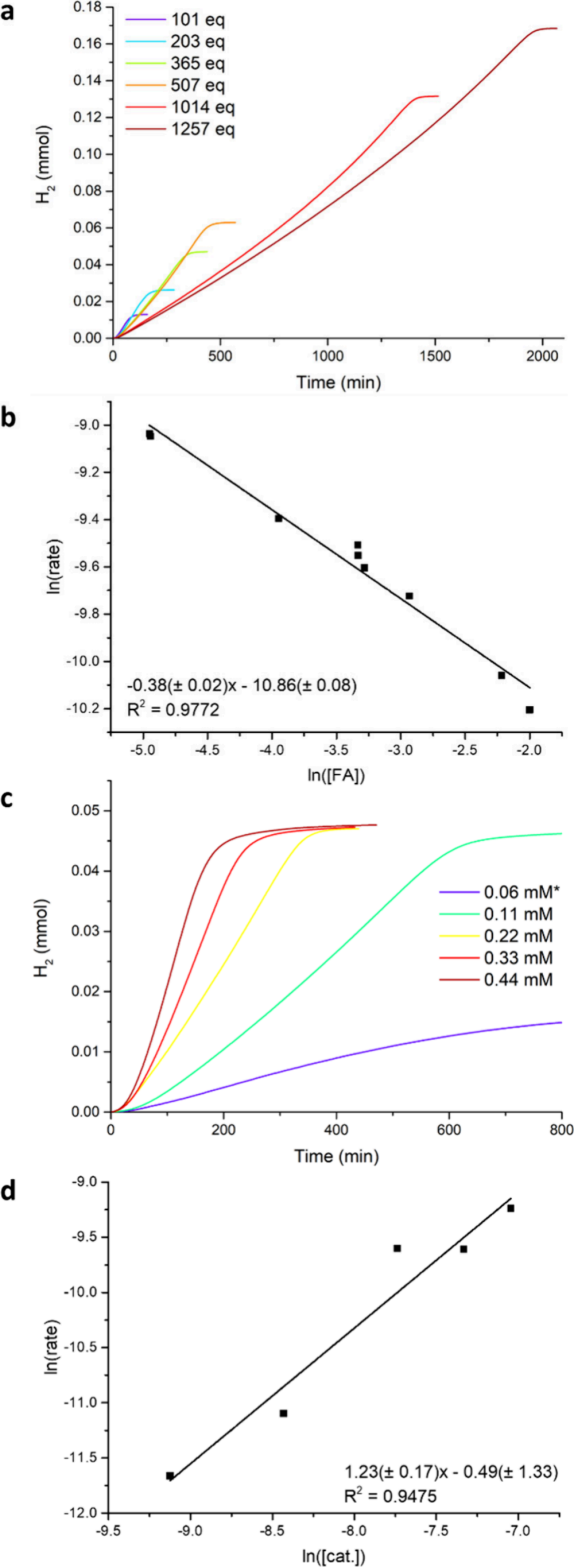
a) The H_2_ production over time of FA dehydrogenation by **1** (0.22 mM) with different amounts of FA added. b) The ln of the rate plotted against the ln of the formic acid concentration for the experiments from a. c) The H_2_ production over time of FA (80 mM) dehydrogenation with different amounts of catalyst **1** (2.2–0.3 mol % Cu). d) The ln of the rate plotted against the ln of the catalyst concentration for the experiments in c. All reactions were performed at 50 °C and the rate was taken as the average conversion in mmol/min over the first 40 min.*reaction with 0.06 mM **1** only reaches ∼35% yield. The S-shape of the traces in a and c is due to the equilibration of the system with H_2_ gas which is inherent to the online GC setup.

Next, we sought to obtain more insight into the mechanism of FA dehydrogenation catalyzed by **2**. To this end, we measured the kinetics of the reaction using an online GC for the detection of H_2_. For these reactions, a low catalyst concentration is used to ensure that **2** is fully dissolved during the reaction. We observe near quantitative formation of H_2_, in line with the observed selectivity of the reaction discussed above, and at 2.2 mol % Cu loading we are able to reach full conversion at 50 °C within 8 h (TON 45 per Cu). With this setup, the rate law of the FA dehydrogenation reaction was determined. Surprisingly, the observed order in FA concentration is –0.38 ± 0.02 (Figure 3 a-b), showing that the reaction is substrate inhibited. This is also evident in the individual kinetic traces which show an increase in rate as FA is consumed during the reaction. The observed order in catalyst concentration is 1.23 ± 0.17 (Figure 3 c-d). Since no reaction was observed upon the addition of a mixture of H_2_ and CO_2_ to **1**, and since the pressure in our system is constant, we assume that the rate under these conditions does not depend on H_2_ and CO_2_. This leads to the observed rate law in eq 1 below.
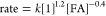
1

## pH Effects

**Scheme 2:**
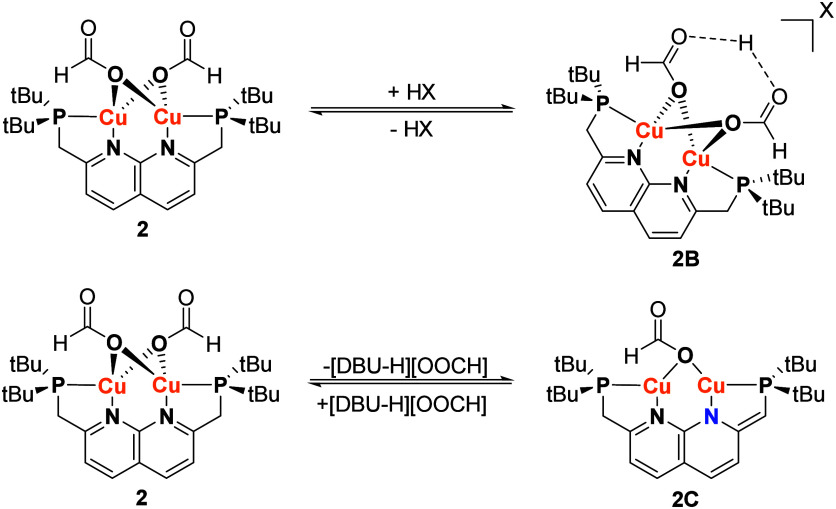
Proposed Reaction of **2** with Acids to Form **2B** (Top) and the Proposed Deprotonation of **2** by DBU to Form **2C** (Bottom) Note that the position of H^+^ in **2B** formate binding modes in **2B** and **2C** is proposed.

Since the catalytic dehydrogenation of FA by complex **1** is inhibited by the FA substrate, we investigated the influence of acids and bases on this reaction. Using a protonated analogue of complex **1** (i.e., **1** + 2 eq HBArF_24_),^19^ the rate of the reaction is diminished to only a fraction of what is observed for **1** (see Figure S24). This implies that protonation of **2** likely is the mechanism by which the catalysis is inhibited. To obtain insights into the protonation of **2**, we again used **2-Ac** to probe its stoichiometric reactivity. Adding 1eq of HBArF_24_ to **2-Ac** leads to small shifts of the resonances corresponding to the PNNP ligand of **2-Ac** in the ^1^H NMR spectrum. These small shifts are also observed when adding an excess of acetic acid to **2-Ac**. We do, however, also see an upfield shift of 0.13 ppm for the acetate resonance in the ^1^H NMR spectrum, which suggests that protonation takes place on the acetate ligands. Important to note is that the color of the mixture changes from brown to yellow when adding an acid to **2-Ac**. Analogously, a similar color difference is observed between the brown crystals of **2** and yellow solutions of **2** containing FA. This all is in line with a hypothesized protonation of **2** to form an off-cycle inactive species **2B** (Scheme 2, top) that is responsible for the substrate inhibition in the FA dehydrogenation reaction. For the structure of **2B**, we are unsure about the position of the proton, although it seems likely that it resides on one or both of the formate ligands. Given the rapid exchange of carboxylate ligands in **2** and **2-Ac** in the presence of free acid observed by NMR analysis, the structure of **2B**, including the position of the proton, is presumably fluctional.

Based on the observed inhibition by acids, we hypothesized that adding a base to the reaction mixture might be beneficial. In the work of Tanase,^11^ they showed that triethylamine (TEA) enhances the catalytic activity of their complex in MeCN-*d*_3_, therefore we also attempted the catalysis with **1** in the presence of TEA. Surprisingly, the addition of TEA does not have an influence on the rate of FA dehydrogenation catalyzed by **1** in THF. This is likely because in THF, the p*K*_a_ of Et_3_NH^+^ is lower than that of carboxylic acids,^20,21^ and hence it also does not significantly affect the equilibrium between **2** and **2B**. However, when using the deprotonated analogue of **1** (Figure S25)^22^ or when using 1,8-diazabicyclo[5.4.0]undec-7-ene (DBU) as a stronger base in small amount (0.3 equiv vs **1**, Figure S29), the reaction proceeds at a slightly lower rate. The addition of 0.3 or 0.6 equiv of DBU with respect to FA leads to a seeming change in mechanism, as now the reaction slows down at lower FA concentration, in contrast to the increase observed for the reaction without a base (Figure S28). In addition, we observe in this case that the solution does not turn yellow after the addition of FA, but instead, the red/orange color persists. This distinct color is an indication that one of the methylene linkers in **2** is deprotonated concomitant with the partial dearomatization of the naphthyridine backbone as has been seen in the previously reported Cu(I) PNNP complexes.^17,22,23^ We hypothesize that DBU deprotonates complex **2** in solution to form complex **2C** (Scheme 2, bottom), which is expected to be red/orange due to its partially dearomatized backbone. To probe this deprotonation stoichiometrically, **2-Ac** was reacted with 1 and with 10 eq of DBU. With 1 eq DBU, mostly peak broadening and a minor new species was observed in ^1^H NMR analysis indicating little deprotonation. However, upon addition of 10 eq of DBU, the solution changed color from brown to red and a new asymmetric species was observed in ^31^P and ^1^H NMR which is consistent with the formation of the acetate analogue of **2C** (**2C–Ac**). Since at this concentration **2C** is in equilibrium with **2C–Ac**, we were able to calculate the p*K*_a_ of the methylene linkers of **2C** in THF, which is 18.^20^

We rationalize the different kinetic behavior by considering that not only the equilibrium between **2** and **2B** influences the rate, but also the equilibrium between **2** and **2C**. In the absence of base the latter equilibrium does not significantly influence the rate of the reaction since the equilibrium between **2** and **2C** lies on the side of **2** due to the excess of acid. However, in the presence of DBU this changes because DBU effectively decreases the acid concentration (by deprotonating and neutralizing the acid) and while this decreases the amount of **2B**, it strongly increases the presence of **2C** and hence inhibits the reaction. Such inhibition by a base is atypical since FA dehydrogenation reactions are typically accelerated by the addition of a base. However, it appears that in the case of **1**, the acidic methylene linkers prevent such a beneficial effect.^11,14,24^

## Kinetic Isotope Effect

**Figure 4:**
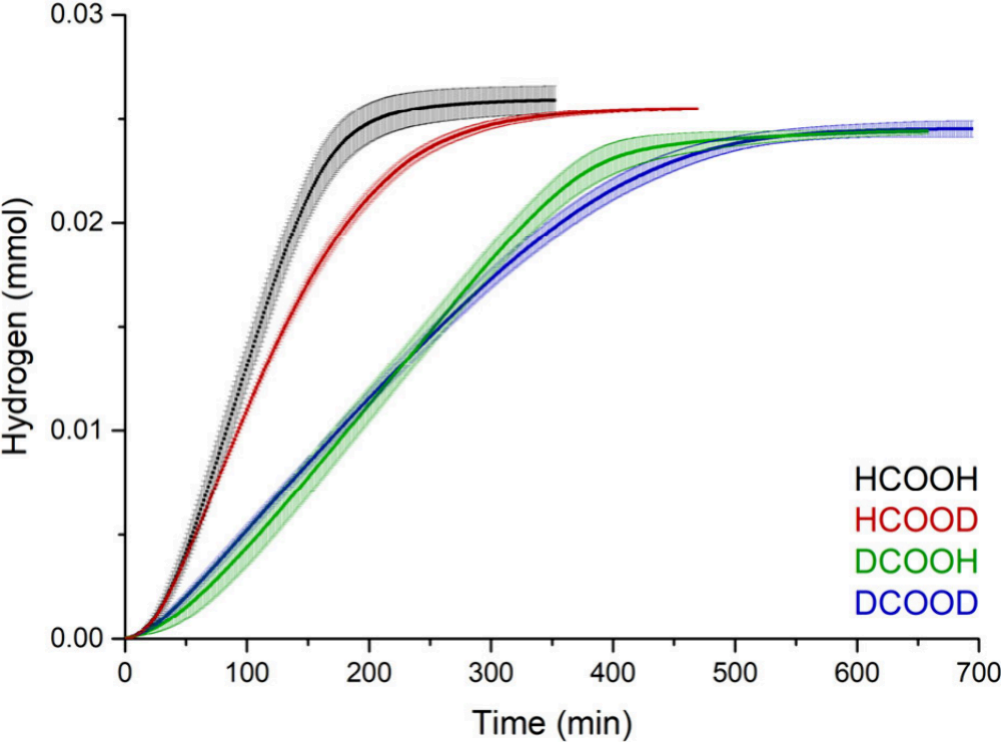
Cumulative H_2_, HD or D_2_ production for the differently deuterium labeled forms of formic acid over time. The average of three measurements was calculated and the error bars show the corresponding standard deviations. Reactions were performed at 50 °C, 0.22 mM concentration of catalyst (**1**) and 44 mM concentration of FA. The average rate over the 38 min after 0.005 mmol conversion for each experiment was used for calculating the KIE (see Supporting Information for details).

To obtain insight into the rate-determining step (RDS), the KIE was determined for both the C–H and O–H bond in formic acid. For this, the kinetics of the dehydrogenation of formic acid were measured for HCOOH, HCOOD, DCOOH and DCOOD in triplicate (Figure 4). For the C–H bond, a primary KIE of 2.4 ± 0.2 was observed, which is typical for this type of reaction.^11,25−27^ This indicates that the rate-determining step of the reaction is the cleavage of this C–H bond. For the O–H bond, a KIE of 1.2 ± 0.1 was found, indicating a secondary KIE which is also consistent with a rate-limiting C–H cleavage.

## Computational Investigation

**Figure 5:**
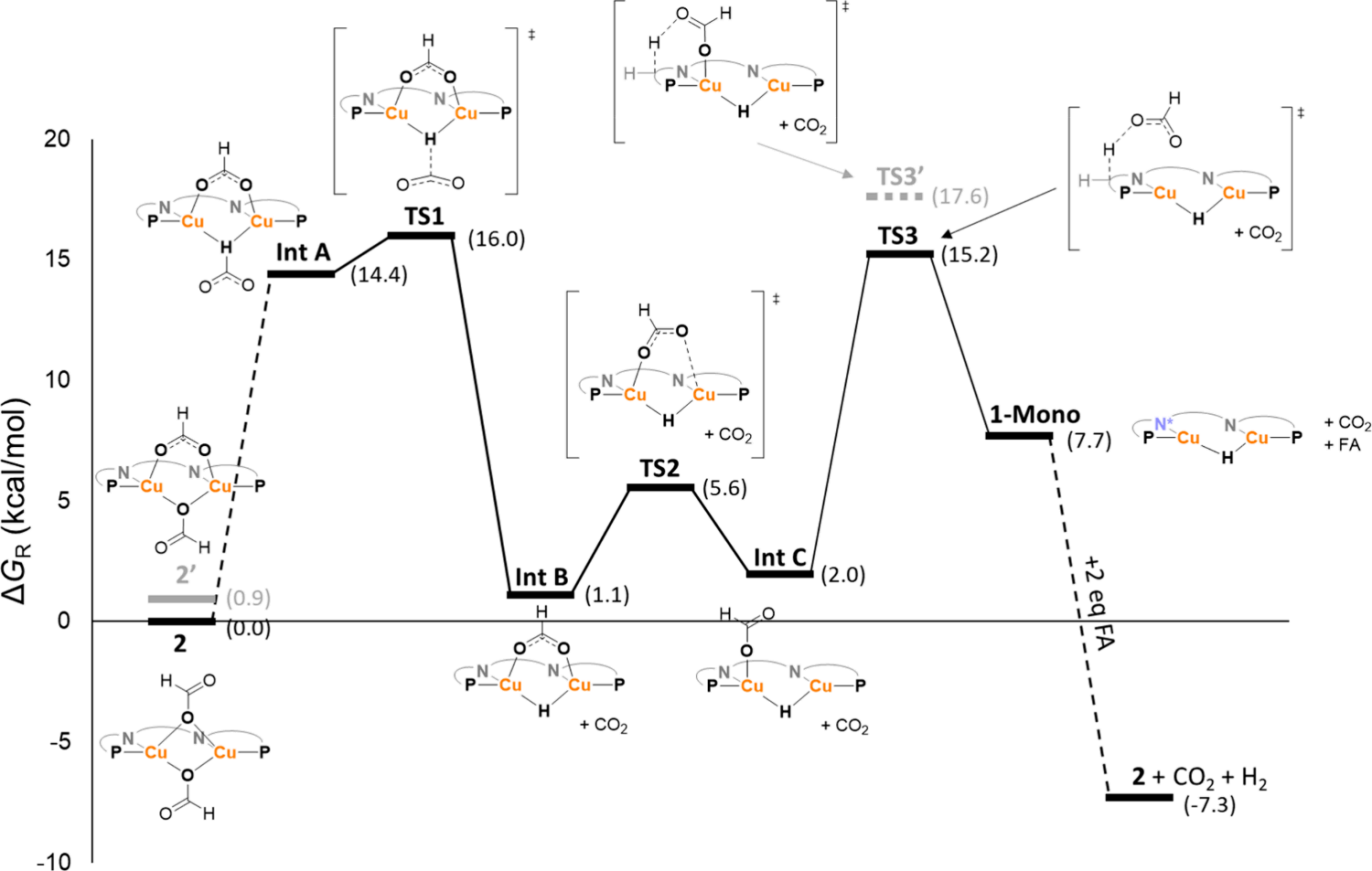
Calculated energy profile for FA dehydrogenation catalyzed by **1**. The energies are Gibbs free energies (at 25 °C) calculated at ωB97XD/def2-TZVP level of theory with SMD(THF) solvent correction. The calculated steps only include those that we were not able to probe experimentally. Dashed lines connect intermediates between which no transition state was found computationally. The feasibility of these steps was evaluated either with a potential energy surface scan or experimentally. The PNNP ligand in the structures is depicted schematically for clarity, the blue N* indicates an anionic nitrogen due to the partial dearomatization of the naphthyridine moiety caused by the deprotonation of one of the methylene linkers. The energies of the different species are shown in brackets for clarity.

To supplement the experimental insights into the mechanism of FA dehydrogenation catalyzed by **1**, we performed computational studies using DFT (at ωB97XD/def2-TZVP level of theory with SMD(THF) solvent correction). Our calculations show that both the terminal formate binding mode (**2**) as well as an η_2_-bridging formate binding mode (**2’**) are energetically accessible with an energy difference of 0.9 kcal/mol (Figure 5) and likely both occur in solution. At the start of the mechanism, one of the formate ligands on **2** tumbles to form **Int A** in which this formate ligand is now coordinated with its hydrogen to the two copper centers.^28^ For this tumbling motion we were not able to locate a transition state, however, a potential energy surface (PES) scan indicates that this transformation has only a small barrier of ∼1 kcal/mol (Figure S42). It should be noted that optimizing **Int A** with the “spectator” formate ligand in a terminal binding mode was unsuccessful, this suggests that the η^2^-binding mode of one of the formates stabilizes this intermediate. From **Int A**, cleavage of the formate C–H proceeds with a low barrier transition state of 1.6 kcal/mol (**TS1**, 16.0 kcal/mol from **2**) to form CO_2_ and **Int B**, which is a η^2^-formate hydride complex. From this point, we envision that the direct protonation of the hydride in **Int B** by FA to form **2** may be possible yielding a productive catalytic cycle that does not require MLC, however, we were not able to verify this pathway computationally. Alternatively, a metal ligand cooperative deprotonation of the backbone could also occur from **Int B**. For such a deprotonation there are two options. First we considered a metal ligand cooperative H_2_ formation with the hydride ligand and a methylene proton from the backbone to yield **2C**.^29^ However, a PES scan indicated that the barrier for this reaction would be >30 kcal/mol which is not feasible for a reaction that occurs also at room temperature (see Figure S45).^30^ Alternatively the η_2_-bridging “spectator” formate ligand in **Int B** can dissociate from one of the copper centers to form **Int C** via **TS2**.^31^ The formate ligand can now first dissociate from the complex (which is an endergonic but barrierless process, see Figure S43) and then abstract a proton from the backbone via **TS3** to form the monomeric analogue of **1** (**1-mono**) and a FA molecule. A concerted deprotonation from **Int C** to **1-mono** was also probed but according to our calculations that is too high in energy (17.6 kcal/mol with respect to **2**, see Figure S44). **1-mono** can then either directly react with 2 equiv of FA to form **2** or it can rapidly dimerize to form **1**^17^ which then reacts with 4 equiv FA to form **2** closing the catalytic cycle. This metal ligand cooperative pathway is supported by the observed formation of **1** upon depletion of FA, which indicates that at least in the last cycle this MLC-pathway is likely active.

In the calculated mechanism, cleavage of the formate C–H bond via **TS1** is the rate-determining step with a barrier of 16.0 kcal/mol. This is consistent with the observed primary KIE of 2.4 ± 0.2 for this bond. Moreover, the experimentally observed KIE is reproduced reasonably well by the DFT calculated value of 3.8 (at 50 °C). In a dinuclear complex, two potential modes of formate C–H cleavage (hydride abstraction or a bimetallic β-hydride elimination) can be envisioned as was also proposed by Tanase et al. for their dicopper system.^11^ In their case, they were not able to computationally locate either transition state and were therefore unable to distinguish these. In our case, we find that the hydride abstraction pathway from **TS1** is more favorable than the alternative bimetallic β-hydride elimination (see Figure S46). Based on these observations, we propose that the reason that dicopper complexes are able to catalyze FA dehydrogenation under milder conditions than their mononuclear counterparts lies in the metal–metal cooperativity in the rate determining hydride abstraction step. Previous work on dicopper hydrides has shown that the electron deficient multicenter two-electron bonding stabilizes the copper hydride core in comparison to mononuclear analogues.^22,32,33^ We reason that **TS1**, in which the dicopper hydride intermediate (**Int B**) is formed, is also stabilized through the same electronic effects. This stabilization leads to a lower reaction barrier of this rate-determining step, and hence allows for lower reaction temperatures.

## Mechanistic Proposal

**Scheme 3:**
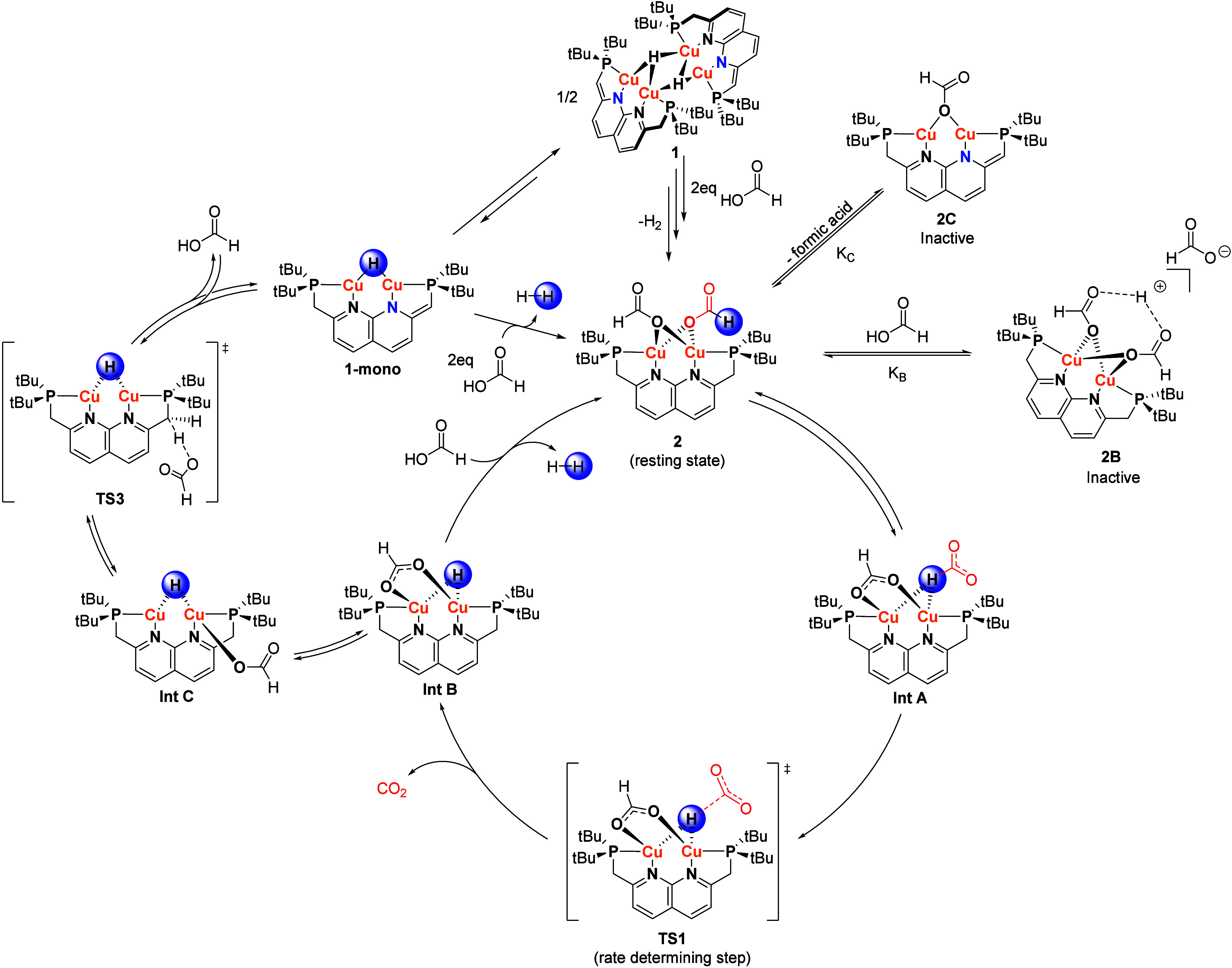
Proposed Mechanistic Cycle for FA Dehydrogenation Catalyzed by Complex **1**

The full mechanistic proposal combining both the experimental and computational findings is shown in Scheme 3. The first step in the mechanism is the reaction of **1** with formic acid to form complex **2**. Although this step entails the reaction of **1** with 4 equiv of formic acid, this reaction has no observable intermediates (see Figure S21 for details). Complex **2** is proposed to be the resting state of the catalyst since the NMR spectra observed during the reaction are consistent with this species. Based on the observed reactivity of **2-Ac** with acids, as well as the inhibition of the FA dehydrogenation reaction by formic acid, we propose an equilibrium between **2** and off-cycle **2B** (Scheme 2) that is dependent on the acid concentration. This is in line with the observed negative order of –0.38 ± 0.02 in formic acid which is supported by modeling of the expected rate law (see Supporting Information).^34^ Complex **2** then goes through a rate-limiting hydride abstraction as C–H cleavage step as is indicated by the observed KIE as well as by our calculations. The calculated energy barrier for the reaction of 16.0 kcal/mol is reasonable considering the reaction rate at low FA concentrations (see SI for details). From **Int B** which is formed after the RDS, the catalytic cycle can be either closed by direct protonation of the hydride ligand to form **2** or by MLC FA loss to regenerate **1-mono**/**1**. The latter pathway is consistent with our observation that in THF, **1** is formed again after the reaction.

## Conclusion and Outlook

Complex **1** constitutes the third example of a well-defined multinuclear copper(I) complex that catalyzes the dehydrogenation of formic acid under mild conditions. Our mechanistic investigations indicate that bisformate complex **2** is the catalyst resting state. The rate-determining step of this mechanism is a C–H cleavage that goes via a hydride abstraction mechanism rather than the β-hydride elimination pathway that was previously also postulated. These findings provide important mechanistic support for the hypothesis^11^ that multicopper active sites provide stabilization for the hydride that is formed in the rate-determining step, thereby enabling facile catalytic formic acid dehydrogenation.

The FA dehydrogenation catalyzed by **1** was surprisingly found to be substrate inhibited due to an off-cycle protonated species. Interestingly, base additives that commonly increase the rate of catalytic FA dehydrogenation reactions, were found to decrease the reaction rate in our system. This is due to the deprotonation of the acidic methylene linkers on the PNNP ligand of the catalyst, which also inhibits the reaction. To improve the compatibility with external bases and potentially combat the substrate inhibition, future efforts will focus on employing PNNP ligands without acidic methylene protons, such as the example very recently reported by the group of Tilley,^35^ or a permethylated analogue as was previously proposed by our group,^36^ for copper catalyzed FA dehydrogenation. This could also shed more light on the feasibility of a non-MLC pathway (i.e., by direct protonation of the hydride in **Int B**) for copper catalyzed FA dehydrogenation.

## Materials and Methods

### Generation of Complex **2**

[^*t*-Bu^PNNP*Cu_2_H]_2_ (5.6 mg, 4.9 μmol, 1 equiv) was dissolved in deuterated MeCN (0.7 mL). Once a red suspension was obtained, formic acid (2 μL, 49 μmol, 10 equiv) was added to obtain a brown solution that rapidly changed into a yellow solution. This mixture was analyzed by NMR spectroscopy without additional workup, since (albeit slowly) the complex decomposes the formic acid forming CO_2_ and H_2_ gas. After consumption of much of the excess FA (upon heating to 50 °C for 1 h) and leaving the solution to cool at rt, the complex crystallizes out as orange needles suitable for X-ray diffraction. These crystals were also used for obtaining an IR spectrum of **2**. Redissolving these crystals did not yield clean NMR spectra due to the composition of **2** into **1** as described. Analogously, complex **2** can be prepared in THF, in which case the formic acid decomposition is faster. After the consumption of excess FA, the complex decomposes into complex **1** again. ^1^H NMR (400 MHz, CD_3_CN, 298 K): δ_H_ (ppm) = 8.39 (d, ^3^J_H,H_ = 8.3 Hz, 2H), 8.25* (s, 2H*), 7.67 (d, ^3^J_H,H_ = 8.3 Hz, 2H), 3.50 (d, ^2^J_H,P_ = 8.3 Hz, 4H), 1.27 (d, ^3^J_H,P_ = 13.5 Hz, 36H). *This peak is a combination of the C*H* resonance of free formic acid and the formic acid bound to complex **2** due to fast exchange of the formate ligands. Hence the position of this resonance shifts according to the amount of formic acid present in solution and as such, the peak integral is higher than expected for the complex. ^13^C NMR (101 MHz, CD_3_CN, 298 K): δ_C_ (ppm) = 166.6 (s), 165.2–164.3 (m), 154.0 (s), 139.6 (s), 124.7 (d, ^3^J_C,P_ = 2.9 Hz), 121.6 (s), 118.3 (s), 68.25 (s), 33.6 (d, ^1^J_C,P_ = 9.0 Hz), 32.7 (d, ^1^J_C,P_ = 14.2 Hz), 29.3 (d, ^2^J_C,P_ = 8.6 Hz). ^31^P NMR (162 MHz, CD_3_CN, 298 K): δ_P_ (ppm) = 35.0 (bs). IR-ATR (cm^–1^): 3252, 3049, 2941, 2896, 2863, 2791, 2586, 2324, 1626(s, HCO_2_), 1608, 1596, 1504, 1538, 1466, 1430, 1416, 1391, 1367, 1286, 1178, 1163, 1130, 1022, 937, 871, 820, 796, 760, 598, 518, 477, 419.

### Synthesis of complex **2-Ac**

Complex **1** (19.7 mg, 17.2 μmol, 1 equiv) was suspended in MeCN (4 mL). Degassed glacial acetic acid (3.5 μL, 61 μmol, 3.6 equiv) was added and the mixture was stirred for 1.5 h. Upon addition of the acetic acid, the red suspension turned brown and much more of the complex went into solution. The suspension was filtered leaving a red residue and a brown filtrate. The residue was washed with MeCN (2 mL) with which barely any color came off. The combined MeCN fractions were dried leaving a dark brown product. The solid was stripped with 2 mL benzene to yield **2-Ac** as an orange powder (17.2 mg, 72%). Dissolving this powder in benzene again gives a dark brown solution. ^1^H NMR (400 MHz, C_6_D_6_, 298 K): δ 7.33 (d, ^3^*J*_*H,H*_ = 8.1 Hz, 2H), 6.66 (d, ^3^*J*_*H,H*_ = 8.1 Hz, 2H), 2.99 (d, ^2^*J*_*P,H*_ = 7.4 Hz, 4H), 2.34 (s, 6H) 1.22 (d, ^3^*J*_*H,P*_ = 12.9 Hz, 36H). ^31^P{^1^H} NMR (162 MHz, C_6_D_6_, 298 K): δ 30.7 (bs) ppm. ^13^C {^1^H} APT (101 MHz, C_6_D_6_, 298 K): δ 175.8, 164.8, 154.0, 137.0, 123.2, 120.5, 33.1 (d, ^1^*J*_*C,P*_ = 6.7 Hz), 32.7 (d, ^1^*J*_*C,P*_ = 10.0 Hz), 29.6 (d, ^2^*J*_*C,P*_ = 8.8 Hz), 25.4. IR-ATR (cm^–1^): 3053, 2941, 2899, 2865, 1621, 1601, 1578, 1538, 1505, 1471, 1407, 1365, 1304, 1181, 1068, 1016, 866, 822, 812, 657, 607.

### General Procedure for Formic Acid Dehydrogenation Kinetics

0.6 mL of a stock solution of complex **1** (5.0 mg, 4.4 μmol) in THF (20 mL) was added to a J-Young NMR tube in the glovebox. This sealed tube was consecutively attached to a Schlenk line and cooled to 0 °C in an NMR tube adaptor containing degassed silicon oil with a height equal to or higher than the sample liquid in the NMR tube. After cooling, the cap was removed and with a degassed micro syringe, the appropriate amount of a 10%_V/V_ stock solution of formic acid in THF was added after which the tube was quickly sealed and cooled in an ice bath. On ice, the sample was transported to the GC for H_2_ gas within 5 min. There it was connected (Figure S23) and heated up to 50 °C in a preheated oil bath. After connecting the tube, the GC setup was flushed with N_2_ (10 mL/min) for 50 s after which the flow was reduced to 1 mL/min and a blank was measured. After another minute, the J-Young tube was opened to the system and the measurements were started. The measurement is continued until the amount of H_2_ produced drops under the detection limit.

### Computational Methods

Calculations were performed using Gaussian 16 rev. C01 software.^37^ The ωB97X-D functional developed by Head-Gordon and co-workers was used.^38^ The redefinition of Ahlrichs triple-ζ split valence basis set (def2-TZVP) was used on all atoms unless noted otherwise.^39^ All calculations were performed with an SMD implicit solvation model (Tetrahydrofuran) unless mentioned otherwise.^40^ All intermediates were confirmed to be stationary points by the absence of imaginary vibrational frequencies and the transition states were confirmed by the presence of a singular imaginary frequency. All calculations were performed at 298.15 K except for the KIE calculations which were calculated at 323.15 K. To calculate the KIE, the frequencies of the structures of **2**, **2**’ and **TS1** were calculated with deuterium on both OOC-H̲ positions to obtain the free energy barrier with deuterium. Then using the Arrhenius equation, the ratio between the reaction rates with hydrogen and deuterium was calculated at 323.15 K to obtain the calculated KIE.

All other information pertaining to the materials and methods used in this study is provided in the Supporting Information.

## Data Availability

NMR data files and the DFT output files supporting this work can be obtained free of charge from YODA at 10.24416/UU01-80REW7. CCDC 2332075 (compound **2**) contains the supplementary crystallographic data for this paper. These data can be obtained free of charge from The Cambridge Crystallographic Data Centre via www.ccdc.cam.ac.uk/data_request/cif.

## Supporting Information Available

The Supporting Information contains the . The Supporting Information is available free of charge at https://pubs.acs.org/doi/10.1021/acscatal.4c05008.

Experimental details for this manuscript as well as the synthetic procedures and spectroscopic data of complexes **2** and **2-Ac**, and the crystal structure determination of complex **2**. It also contains the computational methodology and additional computational data for this manuscript (PDF)

## References

[ref1] Goeden G. V.; Huffman J. C.; Caulton K. G. A Cu-(μ-H) Bond Can Be Stronger Than an Intramolecular P⃗Cu Bond. Synthesis and Structure of Cu_2_(μ-H)_2_[Η_2_-CH_3_C(CH_2_PPh_2_)_3_]_2_. Inorg. Chem. 1986, 25 (15), 2484–2485. 10.1021/ic00235a002.

[ref2] Wyss C. M.; Tate B. K.; Bacsa J.; Gray T. G.; Sadighi J. P. Bonding and Reactivity of a μ-Hydrido Dicopper Cation. Angew. Chem., Int. Ed. 2013, 52 (49), 12920–12923. 10.1002/anie.201306736. 24132865

[ref3] Jordan A. J.; Wyss C. M.; Bacsa J.; Sadighi J. P. Synthesis and Reactivity of New Copper(I) Hydride Dimers. Organometallics 2016, 35 (5), 613–616. 10.1021/acs.organomet.6b00025.

[ref4] Nakamae K.; Kure B.; Nakajima T.; Ura Y.; Tanase T. Facile Insertion of Carbon Dioxide into Cu_2_(μ-H) Dinuclear Units Supported by Tetraphosphine Ligands. Chem. Asian. J. 2014, 9 (11), 3106–3110. 10.1002/asia.201402900. 25204731

[ref5] Zhang L.; Cheng J.; Hou Z. Highly Efficient Catalytic Hydrosilylation of Carbon Dioxide by an N-Heterocyclic Carbene Copper Catalyst. Chem. Commun. 2013, 49 (42), 4782–4784. 10.1039/c3cc41838c. 23598425

[ref6] Patrick E. A.; Bowden M. E.; Erickson J. D.; Bullock R. M.; Tran B. L. Single-Crystal to Single-Crystal Transformations: Stepwise CO_2_ Insertions into Bridging Hydrides of [(NHC)CuH]_2_ Complexes. Angew. Chem., Int. Ed. 2023, 62 ( (30), ). 10.1002/anie.202304648. 37221959

[ref7] Romero E. A.; Zhao T.; Nakano R.; Hu X.; Wu Y.; Jazzar R.; Bertrand G. Tandem Copper Hydride–Lewis Pair Catalysed Reduction of Carbon Dioxide into Formate with Dihydrogen. Nat. Catal. 2018, 1 (10), 743–747. 10.1038/s41929-018-0140-3.

[ref8] Beguin B.; Denise B.; Sneeden R. P. A. Hydrocondensation of CO_2_. J. Organomet. Chem. 1981, 208 (1), C18–C20. 10.1016/S0022-328X(00)89188-3.

[ref9] Nakamae K.; Tanaka M.; Kure B.; Nakajima T.; Ura Y.; Tanase T. A Fluxional Cu_8_H_6_ Cluster Supported by Bis(Diphenylphosphino)Methane and Its Facile Reaction with CO_2_. Chem. Eur. J. 2017, 23 (40), 9457–9461. 10.1002/chem.201702071. 28488381

[ref10] Nakamae K.; Nakajima T.; Ura Y.; Kitagawa Y.; Tanase T. Facially Dispersed Polyhydride Cu_9_ and Cu_16_ Clusters Comprising Apex-Truncated Supertetrahedral and Square-Face-Capped Cuboctahedral Copper Frameworks. Angew. Chem., Int. Ed. 2020, 132 (6), 2282–2287. 10.1002/ange.201913533. 31724276

[ref11] Nakajima T.; Kamiryo Y.; Kishimoto M.; Imai K.; Nakamae K.; Ura Y.; Tanase T. Synergistic Cu_2_ Catalysts for Formic Acid Dehydrogenation. J. Am. Chem. Soc. 2019, 141 (22), 8732–8736. 10.1021/jacs.9b03532. 31083993

[ref12] Values given for the pentanuclear complex.

[ref13] Desnoyer A. N.; Nicolay A.; Ziegler M. S.; Torquato N. A.; Tilley T. D. A Dicopper Platform That Stabilizes the Formation of Pentanuclear Coinage Metal Hydride Complexes. Angew. Chem., Int. Ed. 2020, 59 (31), 12769–12773. 10.1002/anie.202004346. 32372506

[ref14] Scotti N.; Psaro R.; Ravasio N.; Zaccheria F. A New Cu-Based System for Formic Acid Dehydrogenation. RSC Adv. 2014, 4 (106), 61514–61517. 10.1039/C4RA11031E.

[ref15] Correa A.; Cascella M.; Scotti N.; Zaccheria F.; Ravasio N.; Psaro R. Mechanistic Insights into Formic Acid Dehydrogenation Promoted by Cu-Amino Based Systems. Inorg. Chim. Acta 2018, 470, 290–294. 10.1016/j.ica.2017.06.043.

[ref16] Phung K.; Thuéry P.; Berthet J.-C.; Cantat T. CO_2_/^13^CO_2_ Dynamic Exchange in the Formate Complex [(2,9-(^*t*^Bu)_2_-Phen)Cu(O_2_CH)] and Its Catalytic Activity in the Dehydrogenation of Formic Acid. Organometallics 2023, 42, 3357 10.1021/acs.organomet.3c00302.

[ref17] Kounalis E.; Lutz M.; Broere D. L. J. Cooperative H_2_ Activation on Dicopper(I) Facilitated by Reversible Dearomatization of an “Expanded PNNP Pincer” Ligand. Chem. Eur. J. 2019, 25 (58), 13280–13284. 10.1002/chem.201903724. 31424132 PMC6856846

[ref18] After 3.5 h, no excess FA is observed anymore; however, full conversion to **1** takes much longer due to the poor solubility of **2** in THF.

[ref19] Full characterization of this complex will be reported in a separate study. Bienenmann R. L. M.; Thangsrikeattigun C.; Schanz A. J.; Lutz M.; Baik M.-H.; Broere D. L. J. Manuscript in preparation.

[ref20] Kütt A.; Selberg S.; Kaljurand I.; Tshepelevitsh S.; Heering A.; Darnell A.; Kaupmees K.; Piirsalu M.; Leito I. PKa Values in Organic Chemistry–Making Maximum Use of the Available Data. Tetrahedron Lett. 2018, 59 (42), 3738–3748. 10.1016/j.tetlet.2018.08.054.

[ref21] The p*K*_a_ of FA in THF has to our knowledge not been reported; however, the p*K*_a_ of acetic acid (22.48) and triethylammonium (12.5) in THF are known (see ref (20)). Mixing TEA and FA in THF leads to some peak broadening of the OH resonance and minor shifts in the formate and TEA resonances (∼0.1 ppm), from which we infer that it is likely the FA and ammonium salt are in equilibrium but that the equilibrium is on the side of FA and TEA.

[ref22] Bienenmann R. L. M.; Schanz A. J.; Ooms P. L.; Lutz M.; Broere D. L. J. A Well-Defined Anionic Dicopper(I) Monohydride Complex That Reacts like a Cluster. Angew. Chem., Int. Ed. 2022, 61 (29), e202202318 10.1002/anie.202202318. PMC9400846 35412679

[ref23] Kounalis E.; Lutz M.; Broere D. L. J. Tuning the Bonding of a μ-Mesityl Ligand on Dicopper(I) through a Proton-Responsive Expanded PNNP Pincer Ligand. Organometallics 2020, 39 (4), 585–592. 10.1021/acs.organomet.9b00829.

[ref24] Loges B.; Boddien A.; Junge H.; Beller M. Controlled Generation of Hydrogen from Formic Acid Amine Adducts at Room Temperature and Application in H_2_/O_2_ Fuel Cells. Angew. Chem., Int. Ed. 2008, 47 (21), 3962–3965. 10.1002/anie.200705972. 18457345

[ref25] Boddien A.; Mellmann D.; Gärtner F.; Jackstell R.; Junge H.; Dyson P. J.; Laurenczy G.; Ludwig R.; Beller M. Efficient Dehydrogenation of Formic Acid Using an Iron Catalyst. Science (1979) 2011, 333 (6050), 1733–1736. 10.1126/science.1206613. 21940890

[ref26] Léval A.; Agapova A.; Steinlechner C.; Alberico E.; Junge H.; Beller M. Hydrogen Production from Formic Acid Catalyzed by a Phosphine Free Manganese Complex: Investigation and Mechanistic Insights. Green Chem. 2020, 22 (3), 913–920. 10.1039/C9GC02453K.

[ref27] Kar S.; Rauch M.; Leitus G.; Ben-David Y.; Milstein D. Highly Efficient Additive-Free Dehydrogenation of Neat Formic Acid. Nat. Catal. 2021, 4 (3), 193–201. 10.1038/s41929-021-00575-4. 37152186 PMC7614505

[ref28] This intermediate was located between **2** and the hydride abstraction transition state **TS1**. This type of hydride abstraction has been proposed or found computationally before for other FA dehydrogenation catalysts. For examples see reference (11), Guan C.; Zhang D.-D.; Pan Y.; Iguchi M.; Ajitha M. J.; Hu J.; Li H.; Yao C.; Huang M.-H.; Min S.; Zheng J.; Himeda Y.; Kawanami H.; Huang K.-W. Dehydrogenation of Formic Acid Catalyzed by a Ruthenium Complex with an N,N′-Diimine Ligand. Inorg. Chem. 2017, 56 (1), 438–445. 10.1021/acs.inorgchem.6b02334. 27983821

[ref29] We deemed this cooperative reaction plausible based on the observed reverse reaction in which the similar [PNNP**Cu_2_OtBu]K complex reacts with H_2_ to form **1** as reported in ref (17).

[ref30] Ryu H.; Park J.; Kim H. K.; Park J. Y.; Kim S. T.; Baik M. H. Pitfalls in Computational Modeling of Chemical Reactions and How to Avoid Them. Organometallics 2018, 37 (19), 3228–3239. 10.1021/acs.organomet.8b00456.

[ref31] We also considered the potential direct protonation of Int C, but analogously to Int B we were unable to verify such a pathway computationally.

[ref32] Speelman A. L.; Tran B. L.; Erickson J. D.; Vasiliu M.; Dixon D. A.; Bullock R. M. Accelerating the Insertion Reactions of (NHC)Cu–H *via* Remote Ligand Functionalization. Chem. Sci. 2021, 12 (34), 11495–11505. 10.1039/D1SC01911B. 34567502 PMC8409461

[ref33] Tran B. L.; Neisen B. D.; Speelman A. L.; Gunasekara T.; Wiedner E. S.; Bullock R. M. Mechanistic Studies on the Insertion of Carbonyl Substrates into Cu-H: Different Rate-Limiting Steps as a Function of Electrophilicity. Angew. Chem., Int. Ed. 2020, 59 (22), 8645–8653. 10.1002/anie.201916406. 32022415

[ref34] It should be noted that a bimolecular transition state in combination with a 2:1 stoichiometry of **2** and FA to form an alternative **2B**, would also be in line with the observed rate law.

[ref35] See M. S.; Ríos P.; Tilley T. D. Diborane Reductions of CO_2_ and CS_2_ Mediated by Dicopper μ-Boryl Complexes of a Robust Bis(Phosphino)-1,8-Naphthyridine Ligand. Organometallics 2024, 43, 1180 10.1021/acs.organomet.4c00122. 38817536 PMC11134609

[ref36] Killian L.; Bienenmann R. L. M.; Broere D. L. J. Quantification of the Steric Properties of 1,8-Naphthyridine-Based Ligands in Dinuclear Complexes. Organometallics 2023, 42 (1), 27–37. 10.1021/acs.organomet.2c00458. 36644418 PMC9832537

[ref37] Frisch M. J.; Trucks G. W.; Schlegel H. B.; Scuseria G. E.; Robb M. A.; Cheeseman J. R.; Scalmani G.; Barone V.; Petersson G. A.; Nakatsuji H.; Li X.; Caricato M.; Marenich A. V.; Bloino J.; Janesko B. G.; Gomperts R.; Mennucci B.; Hratchian H. P.; Ortiz J. V.; Izmaylov A. F.; Sonnenberg J. L.; Williams-Young D.; Ding F.; Lipparini F.; Egidi F.; Goings J.; Peng B.; Petrone A.; Henderson T.; Ranasinghe D.; Zakrzewski V. G.; Gao J.; Rega N.; Zheng G.; Liang W.; Hada M.; Ehara M.; Toyota K.; Fukuda R.; Hasegawa J.; Ishida M.; Nakajima T.; Honda Y.; Kitao O.; Nakai H.; Vreven T.; Throssell K.; Montgomery J. A. Jr.; Peralta J. E.; Ogliaro F.; Bearpark M. J.; Heyd J. J.; Brothers E. N.; Kudin K. N.; Staroverov V. N.; Keith T. A.; Kobayashi R.; Normand J.; Raghavachari K.; Rendell A. P.; Burant J. C.; Iyengar S. S.; Tomasi J.; Cossi M.; Millam J. M.; Klene M.; Adamo C.; Cammi R.; Ochterski J. W.; Martin R. L.; Morokuma K.; Farkas O.; Foresman J. B.; Fox D. J. Gaussian 16, Revision C.01; Gaussian, Inc.: Wallingford CT, 2019.

[ref38] Chai J.-D.; Head-Gordon M. Long-Range Corrected Hybrid Density Functionals with Damped Atom–Atom Dispersion Corrections. Phys. Chem. Chem. Phys. 2008, 10 (44), 6615 10.1039/b810189b. 18989472

[ref39] Weigend F.; Ahlrichs R. Balanced Basis Sets of Split Valence, Triple Zeta Valence and Quadruple Zeta Valence Quality for H to Rn: Design and Assessment of Accuracy. Phys. Chem. Chem. Phys. 2005, 7 (18), 3297 10.1039/b508541a. 16240044

[ref40] Marenich A. V.; Cramer C. J.; Truhlar D. G. Universal Solvation Model Based on Solute Electron Density and on a Continuum Model of the Solvent Defined by the Bulk Dielectric Constant and Atomic Surface Tensions. J. Phys. Chem. B 2009, 113 (18), 6378–6396. 10.1021/jp810292n. 19366259

