# Supplementary material for: Mechanistic Investigation into Copper(I) Hydride Catalyzed Formic Acid Dehydrogenation
Source: ACS Catal. 2024 Oct 7;14(20):15599–608. doi: 10.1021/acscatal.4c05008 (PMC11494502; doi:10.1021/acscatal.4c05008)
Supplement: Supplementary file 1 — cs4c05008_si_001.pdf [file cs4c05008_si_001.pdf]

Supporting information for: “Mechanistic Investigation Into Copper(I) Hydride Catalyzed Formic Acid Dehydrogenation”

Roel L. M. Bienenmann[1]*, Anne Olarte Loyo[1], Martin Lutz[2] & Daniël L. J. Broere[1]*

[1]Organic Chemistry and Catalysis, Institute for Sustainable and Circular Chemistry, Faculty of Science, Utrecht University, Universiteitsweg 99, 3584 CG, Utrecht, The Netherlands. [2]Structural Biochemistry, Bijvoet Centre for Biomolecular Research, Faculty of Science, Utrecht University, Universiteitsweg 99, 3584 CG, Utrecht, The Netherlands. Email: r.l.m.bienenmann@uu.nl, d.l.j.broere@uu.nl.

Contents

General considerations 3
Generation of complex **2** 4
Synthesis of complex **2-Ac** 8
Stoichiometric reactions 12
Reaction of **2-Ac** with acetic acid-d_4 12
Reaction of **2-Ac** with $HBArF_{24}$ 14
Reactions of **2-Ac** with DBU 14
1 eq of DBU 14
10 eq of DBU 16
Reaction of **1** with 2eq of acetic acid 18
FA dehydrogenation catalysis exploration 19
FA dehydrogenation with **1** in an NMR tube 19
Comparison between THF and MeCN as solvents 19
FA-d_2 dehydrogenation with **1** in an NMR tube 20
Formic acid dehydrogenation kinetics 20
General procedure 20
Rate law determination 21
Kinetic isotope effect experiments 22
FA dehydrogenation with protonated and deprotonated analogues of **1** 22
Dicationic $[PNNPCu2H]_2$ 22
Anionic $PNNP^{**}Cu_2H$ 23
Additive experiments 24
Triethylamine 24
Water 24
DBU 25

FA dehydrogenation in MeCN 26
Rate law modelling........ 26
Rate law modelling with **2B** and **2C** considered........ 27
Rate law modelling with only **2B** considered........ 29
Rate law derivation........ 31
Computational methods 32
General considerations........ 32
Structures of intermediates and transition states 33
Potential energy surface scan from **2** to **Int A** 35
Potential energy surface scan formate dissociation from **Int C** 37
Potential energy surface scan concerted backbone deprotonation 38
Potential energy surface scan MLC H_2 formation 39
Potential energy surface scan β-hydride elimination 40
Estimating the reaction rate at room temperature without excess FA........ 40
X-ray crystal structure of **2** 41
References 41

General considerations

All manipulations were performed under N_2 atmosphere inside a glovebox or on a Schlenk line unless mentioned otherwise. The ambient temperature in our glovebox is on average ~27 °C. Glassware was dried at 130 °C in an oven overnight or flame dried prior using it. DCM was collected from an SPS system and degassed by bubbling N_2 through the liquid for at least 30 min. Then it was further dried over molecular sieves (3 Å). Benzene was dried over 4 Å molecular sieves and degassed by bubbling N_2 through the liquid for at least 30 min. THF was distilled over Na/benzophenone ketyl (purple), or over Na dispersed on silica spheres, and degassed by bubbling N_2 through the liquid for at least 30 min. MeCN was dried over molecular sieves (3 Å) and degassed by bubbling N_2 through the liquid for at least 30 min. Subsequently, it was filtered over alumina. Water content in the solvents was tested using Karl-Fisher titration, and THF and benzene were also tested by titration with a Na/benzophenone ketyl solution. Deuterated solvents were degassed using three freeze pump thaw cycles and dried over molecular sieves. MeCN-d_3 was subsequently filtered over alumina. The water content was tested with Karl-Fisher titration (MeCN-d_3) or titration with a Na/benzophenone ketyl solution (C_6D_6). The PNNP ligand[1,2], complex **1**[1], [PNNP**Cu_2H]K18-crown-6[3] and $HBArF_{24}$ (Brookhart's acid)[4] were synthesized according to literature procedures. Other chemicals were obtained from commercial sources. Liquids were degassed using 3 freeze pump thaw cycles before use and solids were degassed under vacuum before use. Note that formic acid and acetic acid were not dried prior to use, although water does not affect the FA dehydrogenation reaction (see Figure S27). HCOOD and DCOOD were obtained as $95\%_{w/w}$ solutions in D_2O and DCOOH was obtained as a $95\%_{w/w}$ solution in H_2O. These were degassed with three freeze-pump-thaw cycles and used as is. NMR spectra were recorded on a Varian MRF 400 equipped with a OneNMR probe and Optima Tune system, a Varian VNMR-S-400 equipped with a PFG probe or a 400 MHz Jeol EZCL G system with an HFX probe and 90 G/cm gradient amplifier. All resonances in ^{1}H NMR were referenced to residual solvent peaks.[5] IR-data was recorded on a PerkinElmer SpectrumTwo Infrared Spectrophotometer equipped with an ATR-probe. Elemental analysis was performed by MEDAC ltd. in the United Kingdom. Quantitative H_2 measurements were performed using an online Interscience CompactGC4.0 G.A.S. containing a 7m*0.32mm Molsieve 5A column, 50 µL sample loop and Thermal Conductivity Detector, to which a J-Young NMR tube was connected (Figure S23). N_2 carrier gas with a flow rate of 1 mL/min was used.

Generation of complex 2

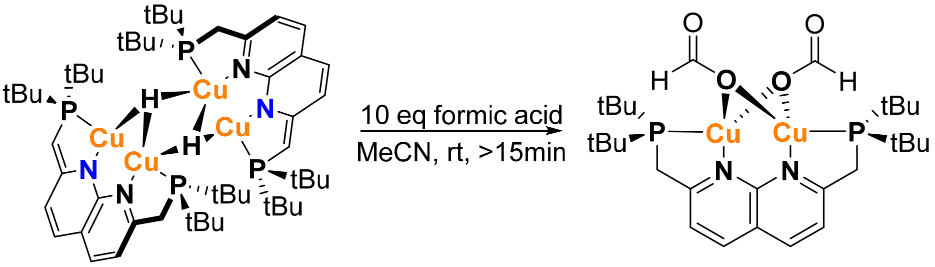

[$^{t\text{-Bu}}$PNNP*Cu_2H]$_2$ (5.6 mg, 4.9 µmol) was dissolved in deuterated MeCN (0.7 mL). Once a red suspension was obtained, formic acid (2 µL, 49 µmol, 10 eq) was added to obtain a brown solution instantly changing to a yellow solution. This mixture was analyzed by NMR spectroscopy without additional work-up, since (albeit slowly) the complex decomposes the formic acid forming CO_2 and H_2 gas. After consumption of much of the excess FA (upon heating to 50°C for 1 hour) and leaving the solution to cool at rt, the complex crystallizes out as orange needles suitable for X-ray diffraction. These crystals were also used for obtaining an IR spectrum of **2**. Redissolving these crystals did not yield clean NMR spectra due to the composition of **2** into **1** as described. Analogously, complex **2** can be prepared in THF, in which case the formic acid decomposition is faster. After the consumption of excess FA, the complex decomposes into complex **1** again.

^{1}H NMR (400 MHz, CD_3CN, 298 K): δ_H (ppm) = 8.39 (d, $^3J_{H,H}$ = 8.3 Hz, 2H), 8.25* (s, 2H*), 7.67 (d, $^3J_{H,H}$ = 8.3 Hz, 2H), 3.50 (d, $^2J_{H,P}$ = 8.3 Hz, 4H), 1.27 (d, $^3J_{H,P}$ = 13.5 Hz, 36H).

^{13}C NMR (101 MHz, CD_3CN, 298 K): δ_C (ppm) = 166.6 (s), 165.2 – 164.3 (m), 154.0 (s), 139.6 (s), 124.7 (d, $^3J_{C,P}$ = 2.9 Hz), 121.6 (s), 118.3 (s), 68.25 (s), 33.6 (d, $^1J_{C,P}$ = 9.0 Hz), 32.7 (d, $^1J_{C,P}$ = 14.2 Hz), 29.3 (d, $^2J_{C,P}$ = 8.6 Hz).

^{31}P NMR (162 MHz, CD_3CN, 298 K): δ_P (ppm) = 35.0 (bs).

IR-ATR (cm^{-1}): 3252, 3049, 2941, 2896, 2863, 2791, 2586, 2324, 1626(s, HCO_2)**, 1608, 1596, 1504, 1538, 1466, 1430, 1416, 1391, 1367, 1286, 1178, 1163, 1130, 1022, 937, 871, 820, 796, 760, 598, 518, 477, 419.

*This peak is a combination of the C*H* resonance of free formic acid and the formic acid bound to complex **2** due to fast exchange of the formate ligands. Hence the position of this resonance shifts according to the amount of formic acid present in solution and as such, the peak integral is higher than expected for the complex. **The assignment of this peak as the asymmetric formate CO_2 stretch is based on analogous observations in literature.[6,7]

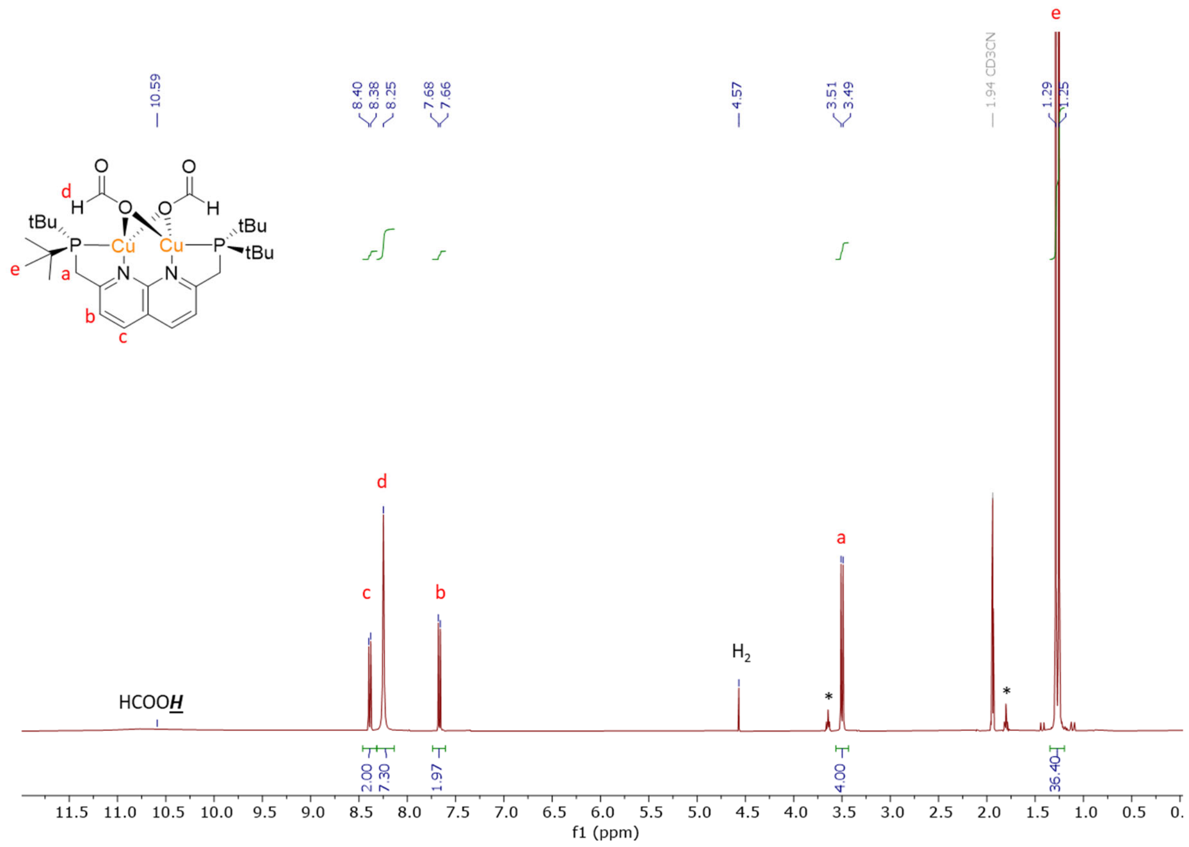

*Figure S1: ^{1}H NMR (MeCN-d_3, 298 K) of complex **2** generated with 10 eq of formic acid.*

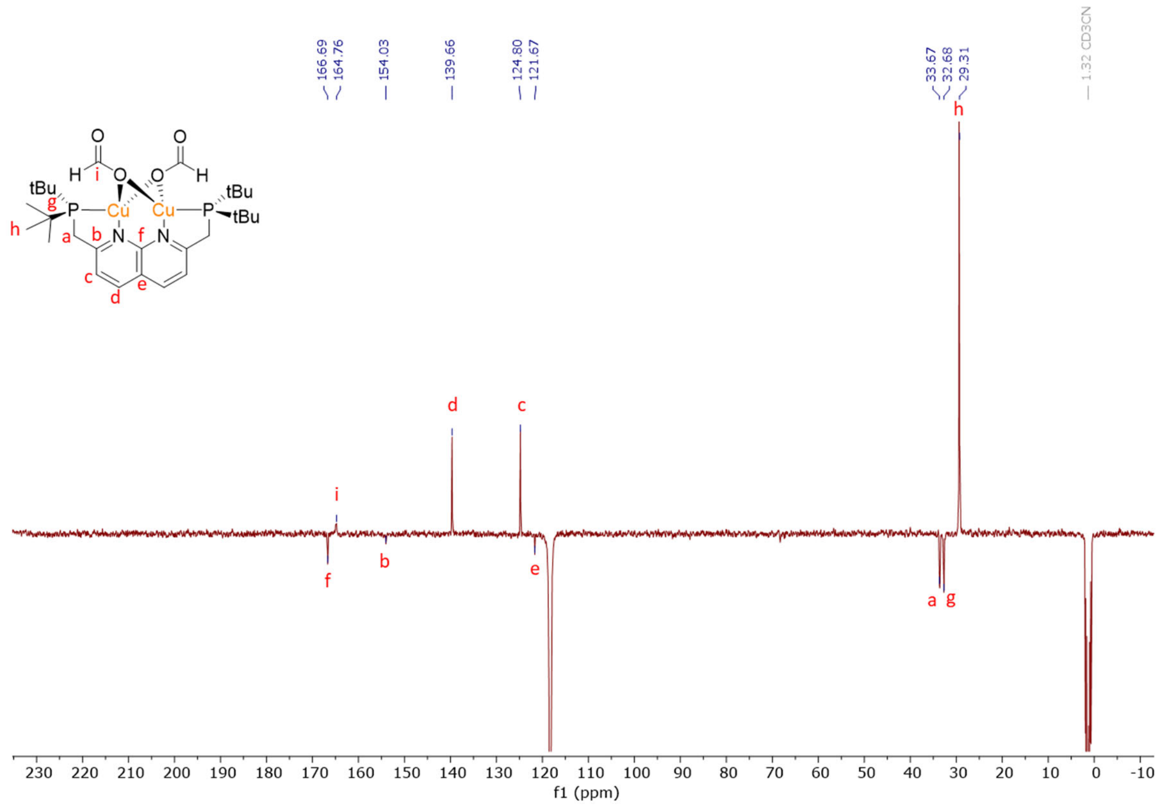

*Figure S2: $^{13}C\{^{1}H\}$ APT NMR (MeCN-d_3, 298 K) of complex **2** generated with 10 eq of formic acid.*

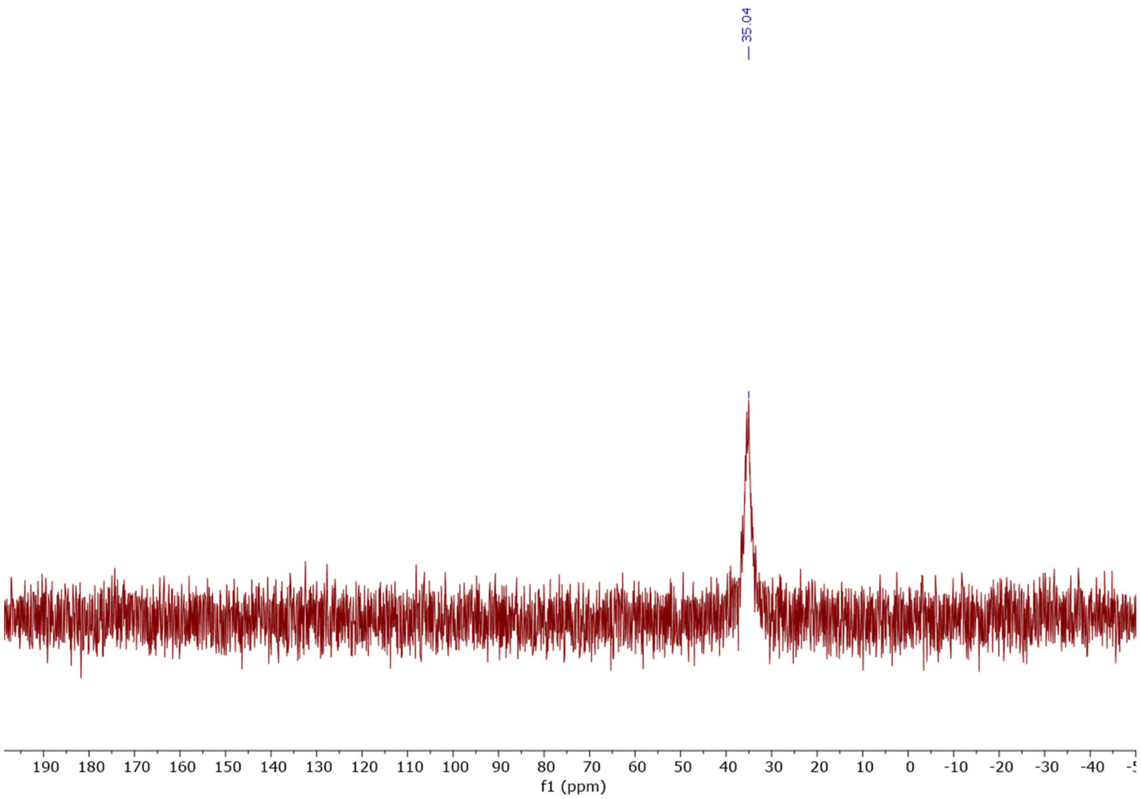

*Figure S3: $^{31}P\{^{1}H\}$ NMR (MeCN-d_3, 298 K) of complex **2** generated with 10 eq of formic acid.*

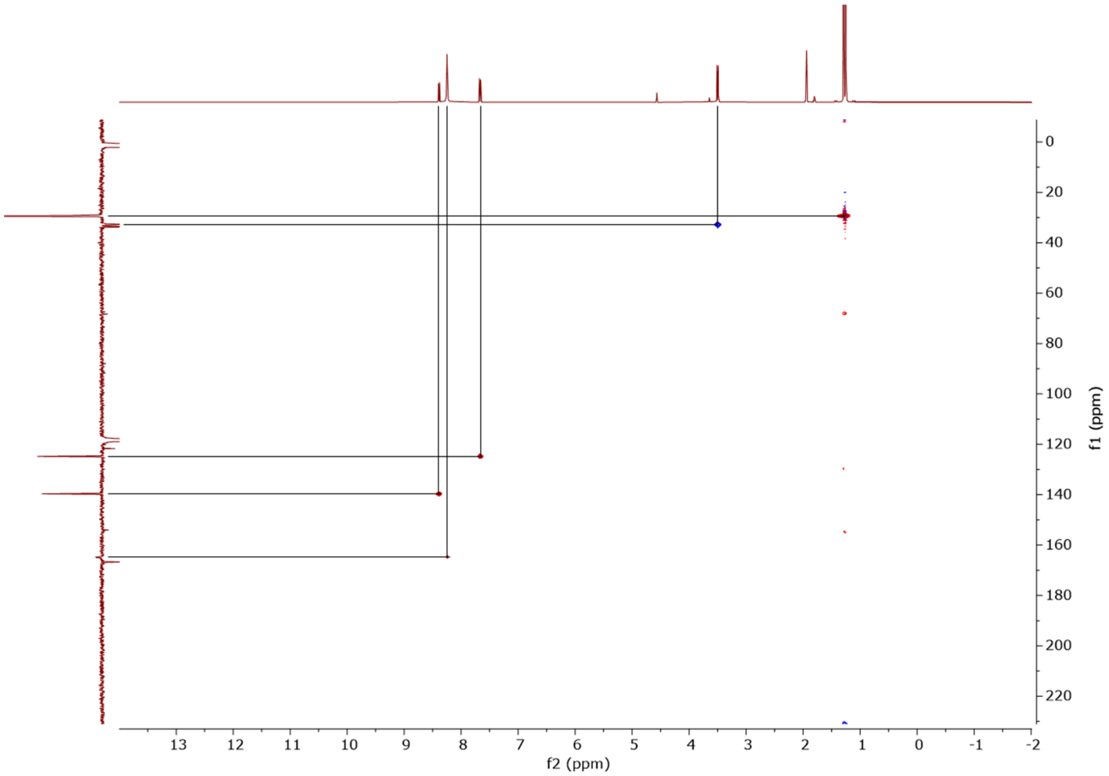

*Figure S4: ^{1}H-^{13}C HSQC NMR (MeCN-d_3, 298 K) of complex **2** generated with 10 eq of formic acid.*

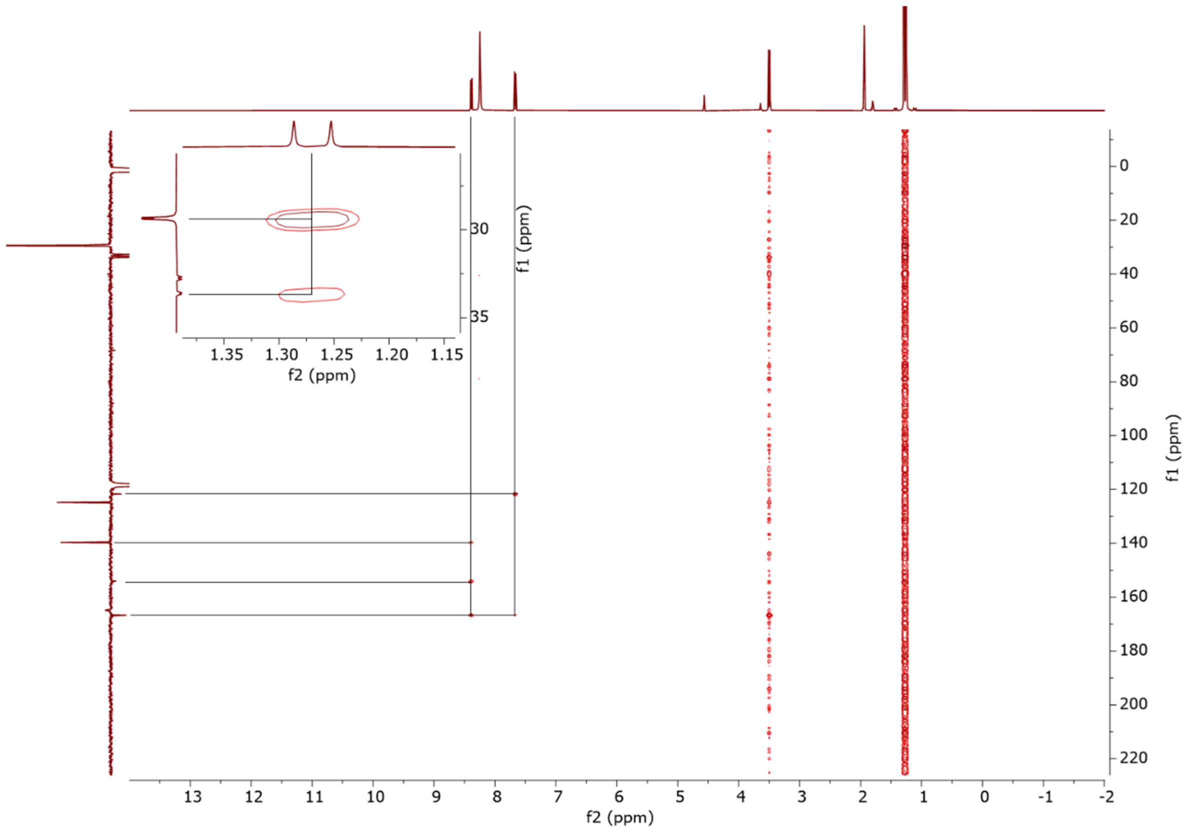

*Figure S5: ^{1}H-^{13}C HMBC NMR (MeCN-d_3, 298 K) of complex **2** generated with 10 eq of formic acid.*

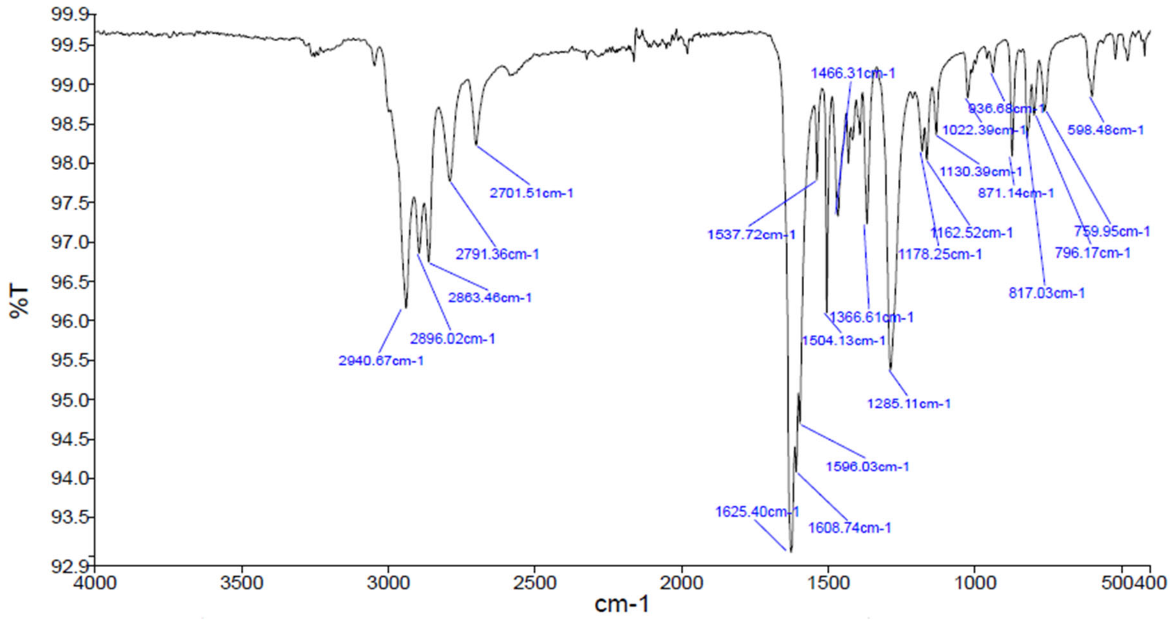

*Figure S6: The ATR-IR spectrum of the crystals of complex **2**.*

Synthesis of complex 2-Ac

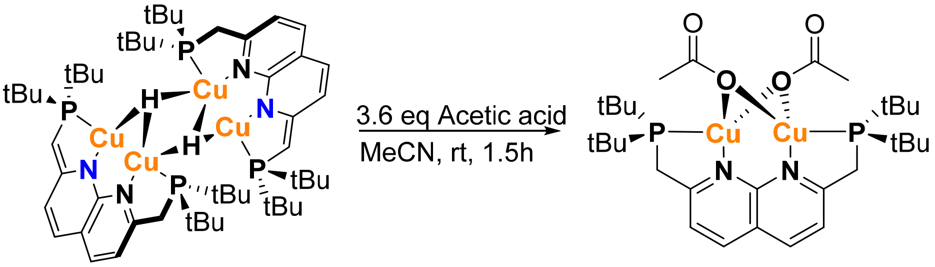

Complex **1** (19.7 mg, 17.2 µmol, 1 eq) was suspended in MeCN (4 ml). Degassed glacial acetic acid (3.5 µl, 61 µmol, 3.6 eq) was added and the mixture was stirred for 1.5h. Upon addition of the acetic acid, the red suspension turned brown and much more of the complex went into solution. The suspension was filtered leaving a red residue and a brown filtrate. The residue was washed with MeCN (2 ml) with which barely any color came off. The combined MeCN fractions were dried leaving a dark brown product. The solid was stripped with 2 ml benzene to yield **2-Ac** as an orange powder (17.2 mg, 72%). Dissolving this powder in benzene again gives a dark brown solution.

^{1}H NMR (400 MHz, C_6D_6, 298 K): δ 7.33 (d, $^3J_{H,H}$ = 8.1 Hz, 2H), 6.66 (d, $^3J_{H,H}$ = 8.1 Hz, 2H), 2.99 (d, $^2J_{P,H}$ = 7.4 Hz, 4H), 2.34 (s, 6H) 1.22 (d, $^3J_{H,P}$ = 12.9 Hz, 36H).

^{31}P{^{1}H} NMR (162 MHz, C_6D_6, 298 K): δ 30.7 (bs) ppm.

13 C {^{1}H} APT (101 MHz, C_6D_6, 298 K): δ 175.8, 164.8, 154.0, 137.0, 123.2, 120.5, 33.1 (d, $^1J_{C,P}$ = 6.7 Hz), 32.7 (d, $^1J_{C,P}$ = 10.0 Hz), 29.6 (d, $^2J_{C,P}$ = 8.8 Hz), 25.4.

IR-ATR (cm^{-1}): 3053, 2941, 2899, 2865, 1621*, 1601, 1578*, 1538, 1505, 1471, 1407, 1365, 1304, 1181, 1068, 1016, 866, 822, 812, 657, 607.

Anal. Calc. for $C_{30}H_{50}Cu_2N_2O_4P_2$: C, 52.09; H, 7.28; N, 4.05. Found C, 51.88; H, 7.09; N, 3.97.

*The IR peak at 1621 cm^{-1} is consistent with the asymmetric CO_2 stretch for a terminally bound acetate ligand[8,9] and the IR peak at 1578 cm^{-1} is consistent with the asymmetric CO_2 stretch for a η^2-bound bridging acetate ligand.[10–12] Therefore, we tentatively assign the presence of both in this IR sample with the same remark as for complex **2**, being that the binding mode of the acetate ligands is likely fluctional. It should be noted that based on this data we cannot assign whether a single molecule of **2-Ph** contains an acetate ligand in each binding mode or that **2-Ph** exists as a mixture with different acetate binding modes.

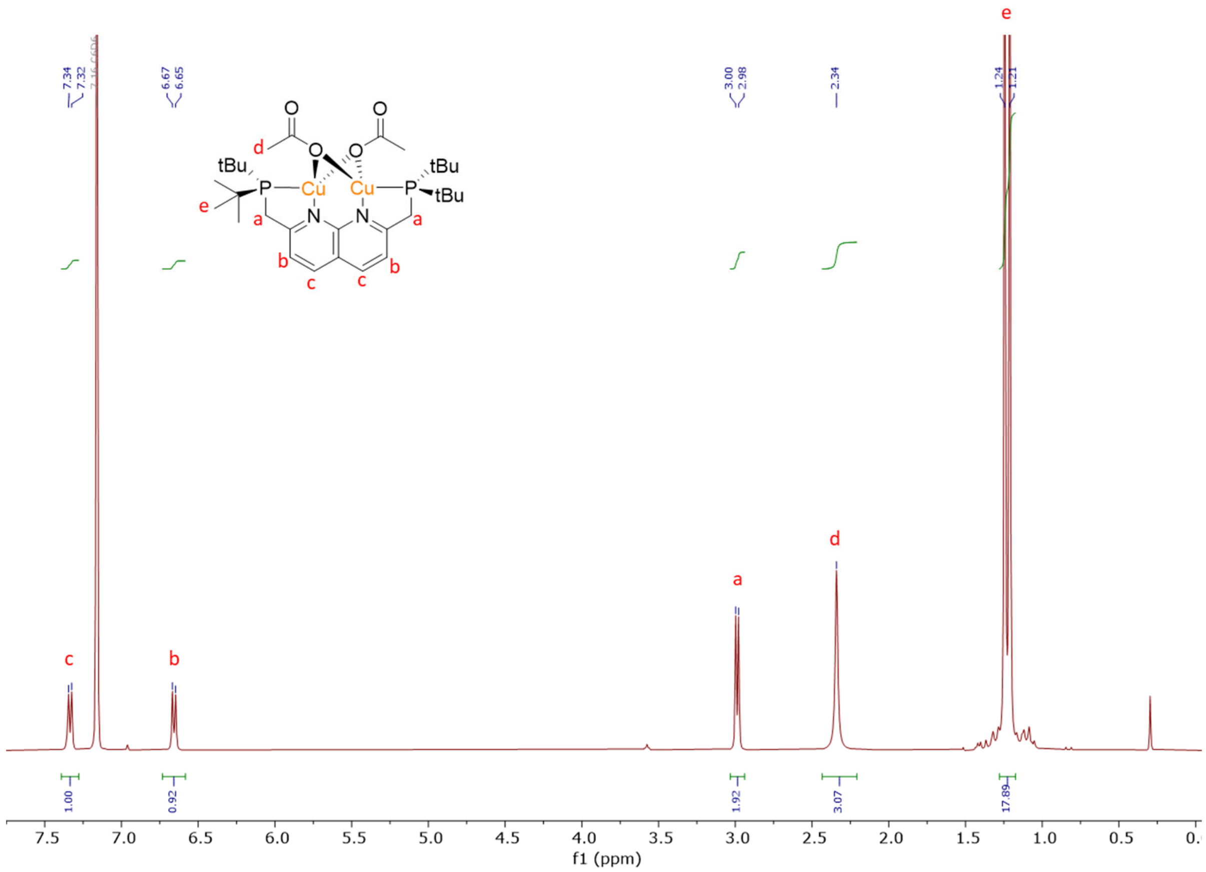

*Figure S7: ^{1}H NMR (C_6D_6, 298 K) of complex **2-Ac**.*

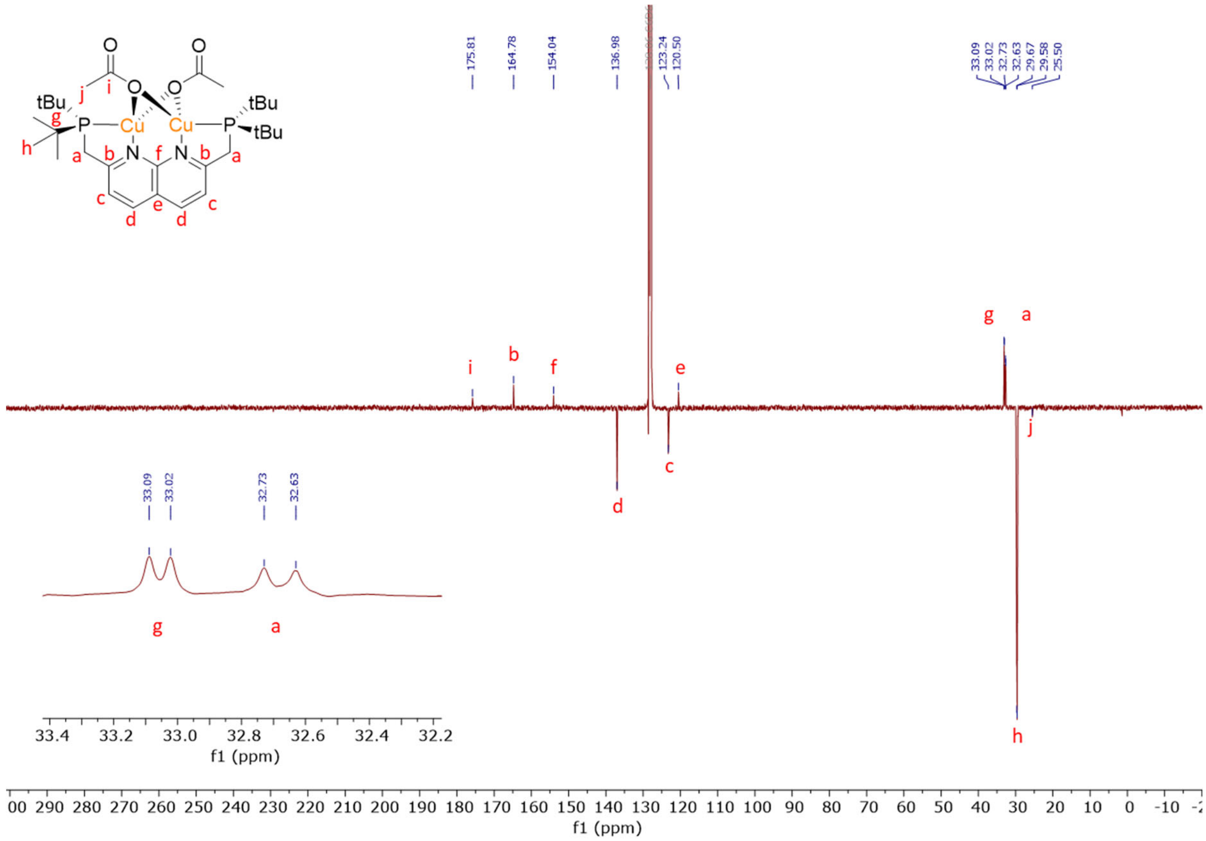

*Figure S8: ^{13}C{^{1}H} APT NMR (C_6D_6, 298 K) of complex **2-Ac**.*

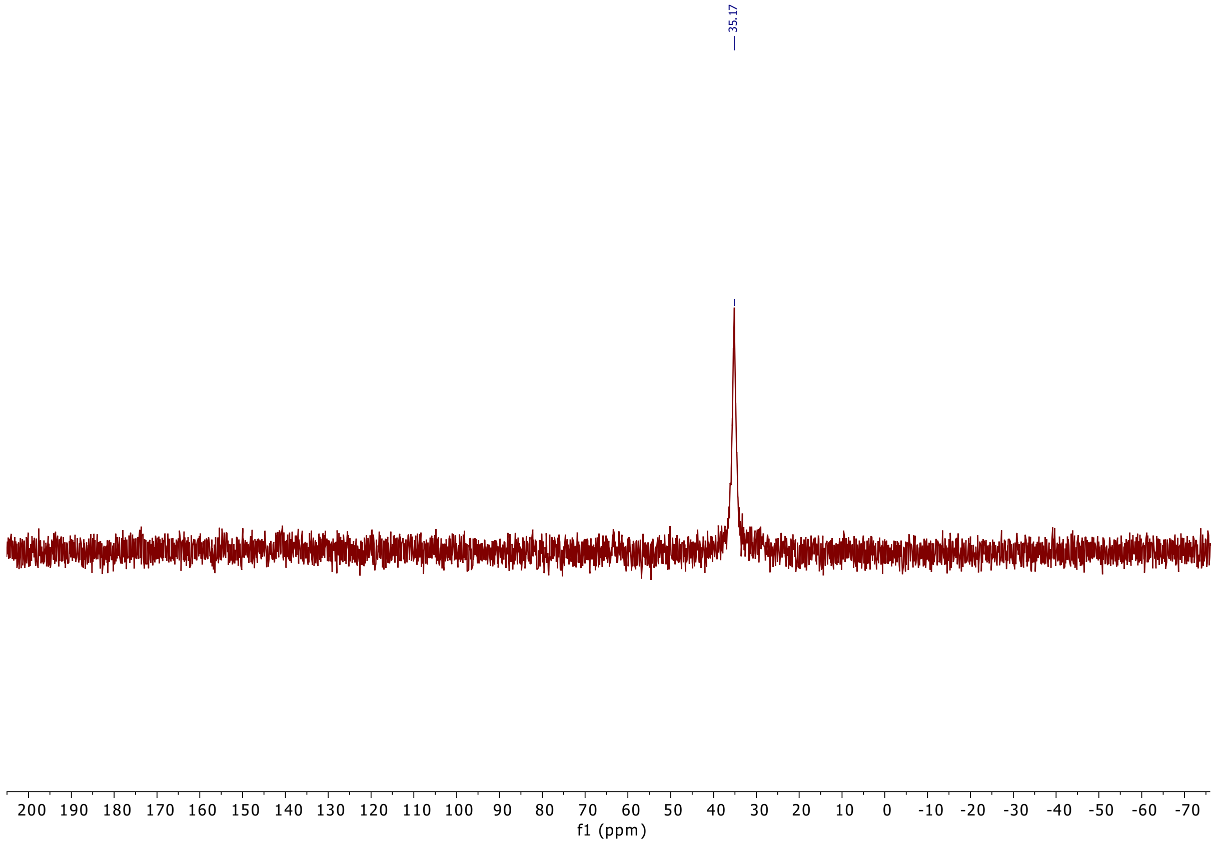

*Figure S9: $^{31}P\{^{1}H\}$ APT NMR (C_6D_6, 298 K) of complex **2-Ac**.*

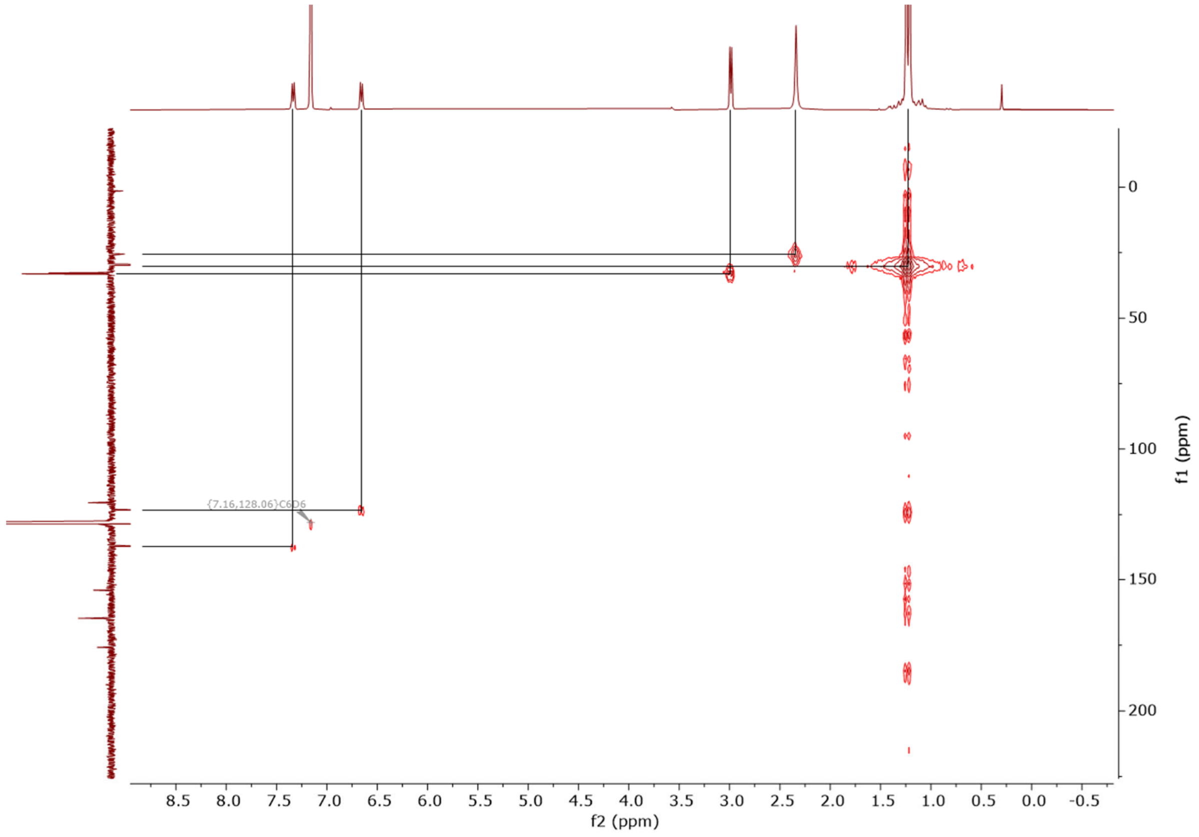

*Figure S10: ^{1}H-^{13}C HMQC NMR (C_6D_6, 298 K) of complex **2-Ac**.*

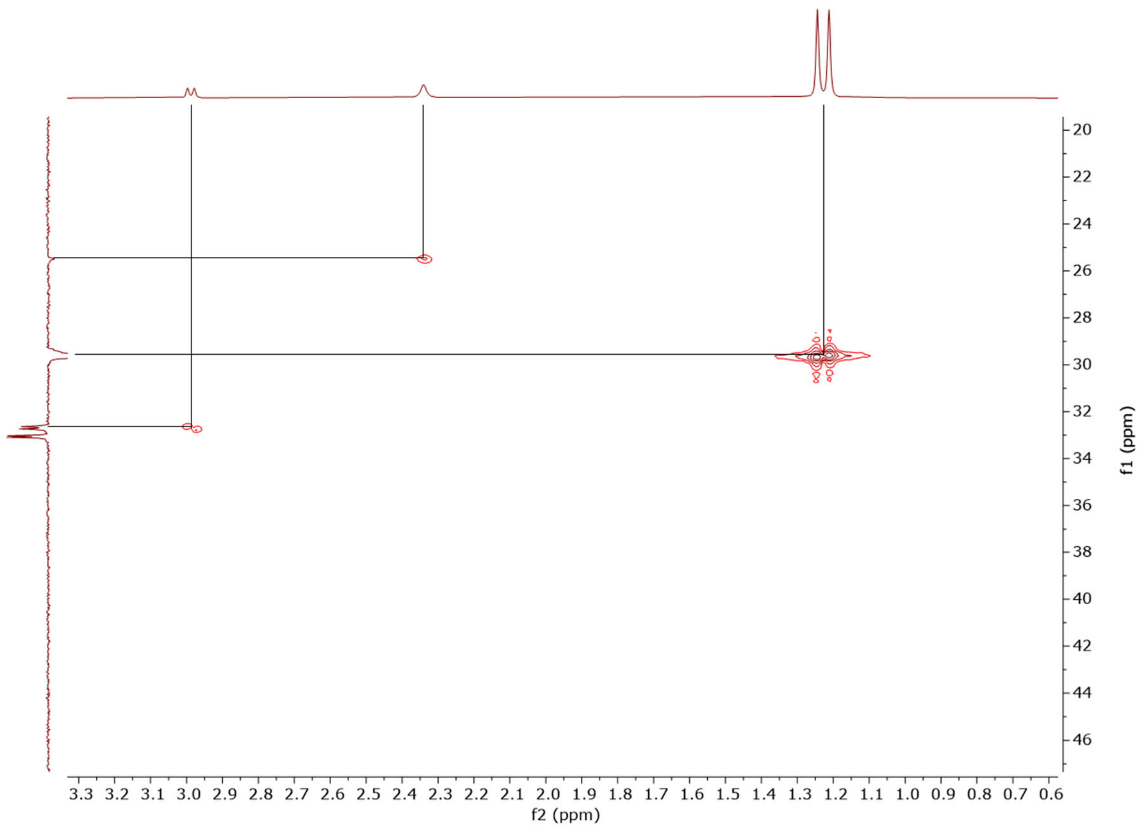

*Figure S11: ^{1}H-^{13}C HMQC NMR (C_6D_6, 298 K) of complex **2-Ac**, over a smaller region for increased resolution.*

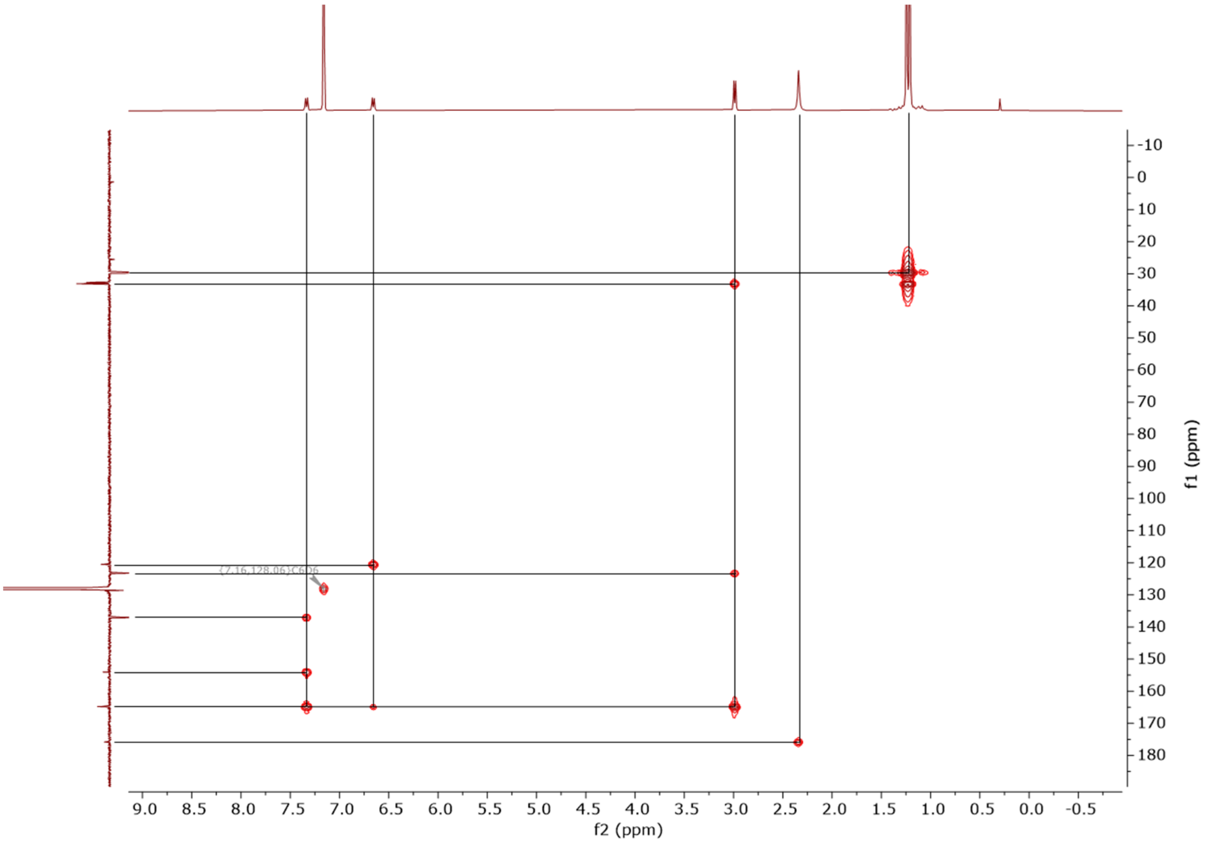

*Figure S12: ^{1}H-^{13}C HMBC NMR (C_6D_6, 298 K) of complex **2-Ac**.*

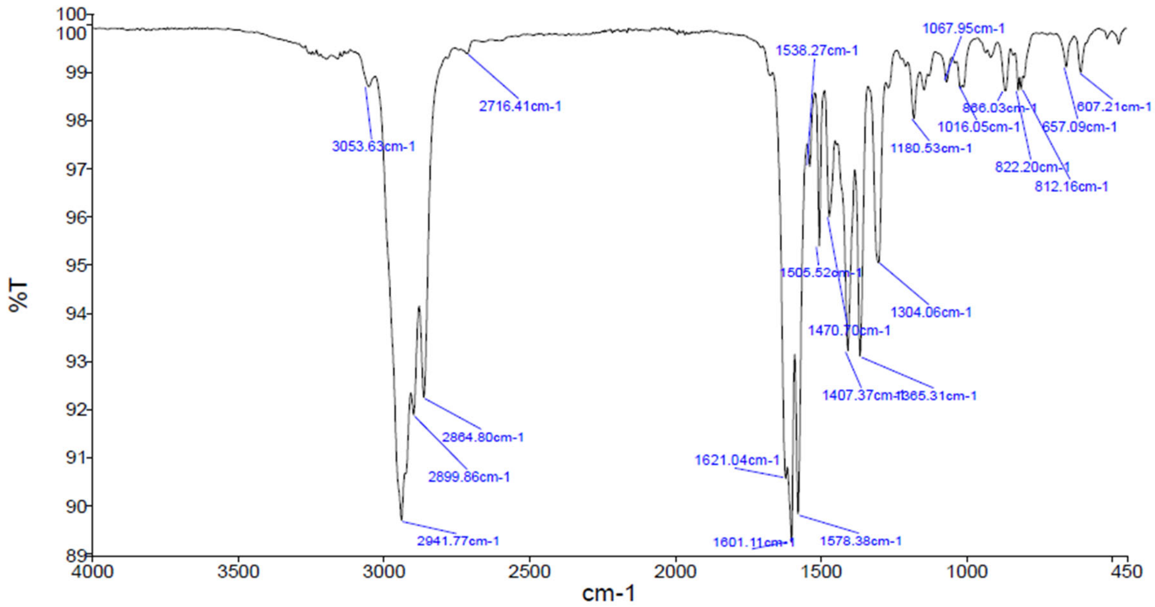

*Figure S13: The ATR-IR spectrum of complex **2-Ac**.*

Stoichiometric reactions

Reaction of 2-Ac with acetic acid-d_4

Complex **2-Ac** (8.3 mg, 12 µmol, 1eq) was dissolved in benzene and acetic acid-d_4 (20 µl, 349 µmol, 29 eq) was added. The mixture turned from brown to lighter brown after the addition. ^{1}H NMR after addition shows the diminishing of the methylene signal for **2-Ac**. Note that the signals in the absence and presence of acid are slightly shifted due to the partial protonation of **2-Ac** which is in fast equilibrium with **2-Ac** itself. The same experiment was performed with the PNNP ligand in THF and with the previously reported PNNPCu$_2$Cl$_2$ complex[1] in DCM, in both cases no exchange of the methylene protons for deuterium was observed.

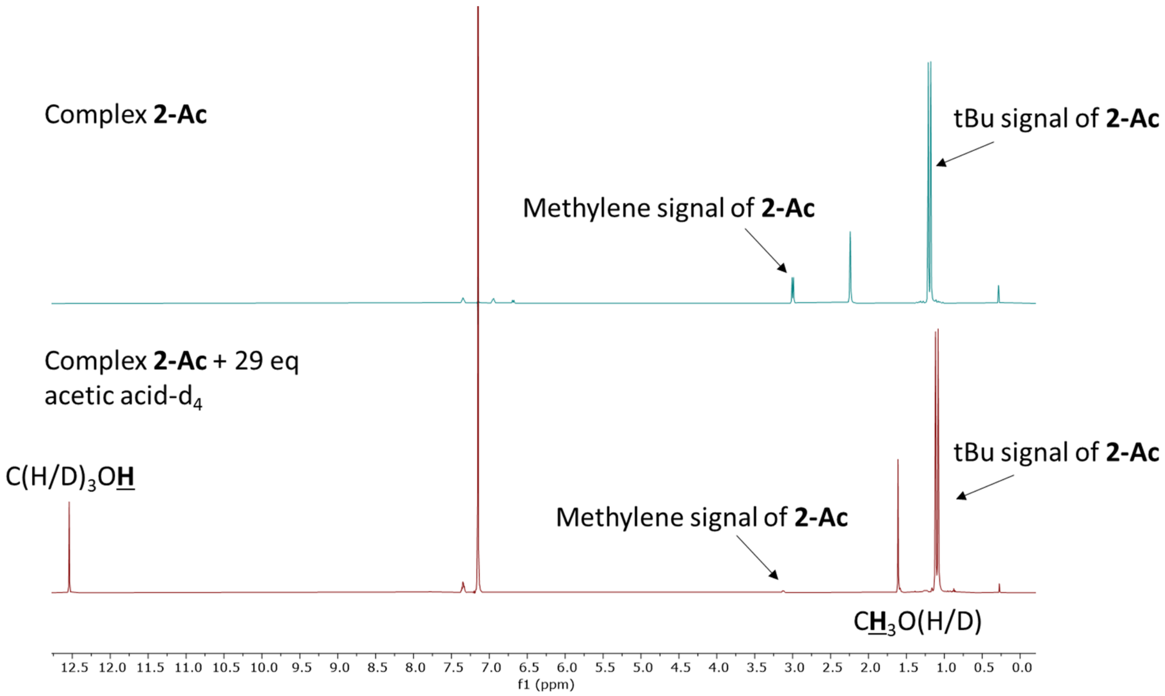

*Figure S14: ^{1}H NMR (C_6H_6, 298 K) of **2-Ac** before (top) and after (bottom) acetic acid-d_4 addition.*

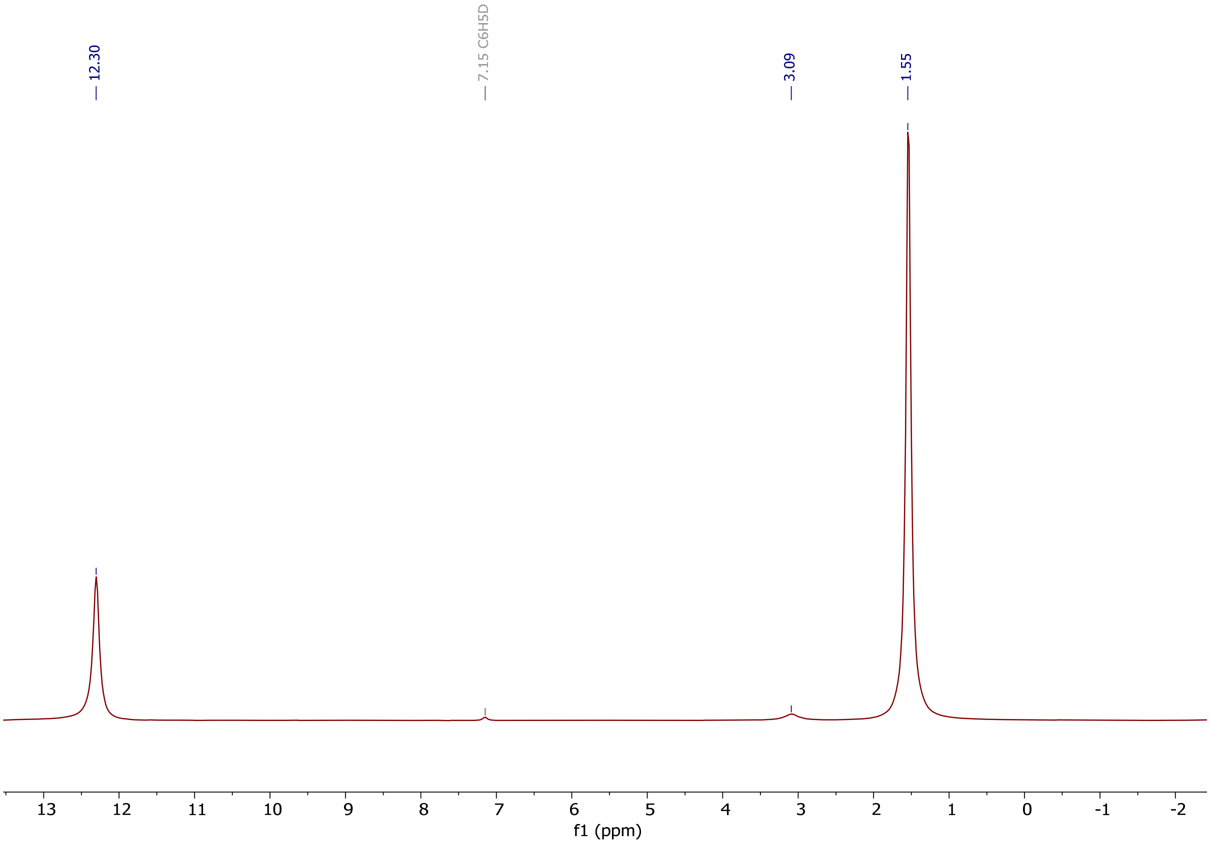

*Figure S15: ^{2}H NMR (C_6H_6, 298 K) of the reaction between **2-Ac** and acetic acid-d_4.*

Reaction of 2-Ac with $HBArF_{24}$

Complex **2-Ac** (3.8 mg, 5.5 µmol, 1eq) was dissolved in MeCN-d_3 yielding a light orange solution. The solution was added to $HBArF_{24}$ (2.7 mg, 2.7 µmol, 0.5 eq), upon which the solution turned more yellow. The ^{1}H NMR spectrum is similar to that of **2-Ac**, however, the peaks are somewhat shifted (similar to what is observed with an excess of acetic acid).

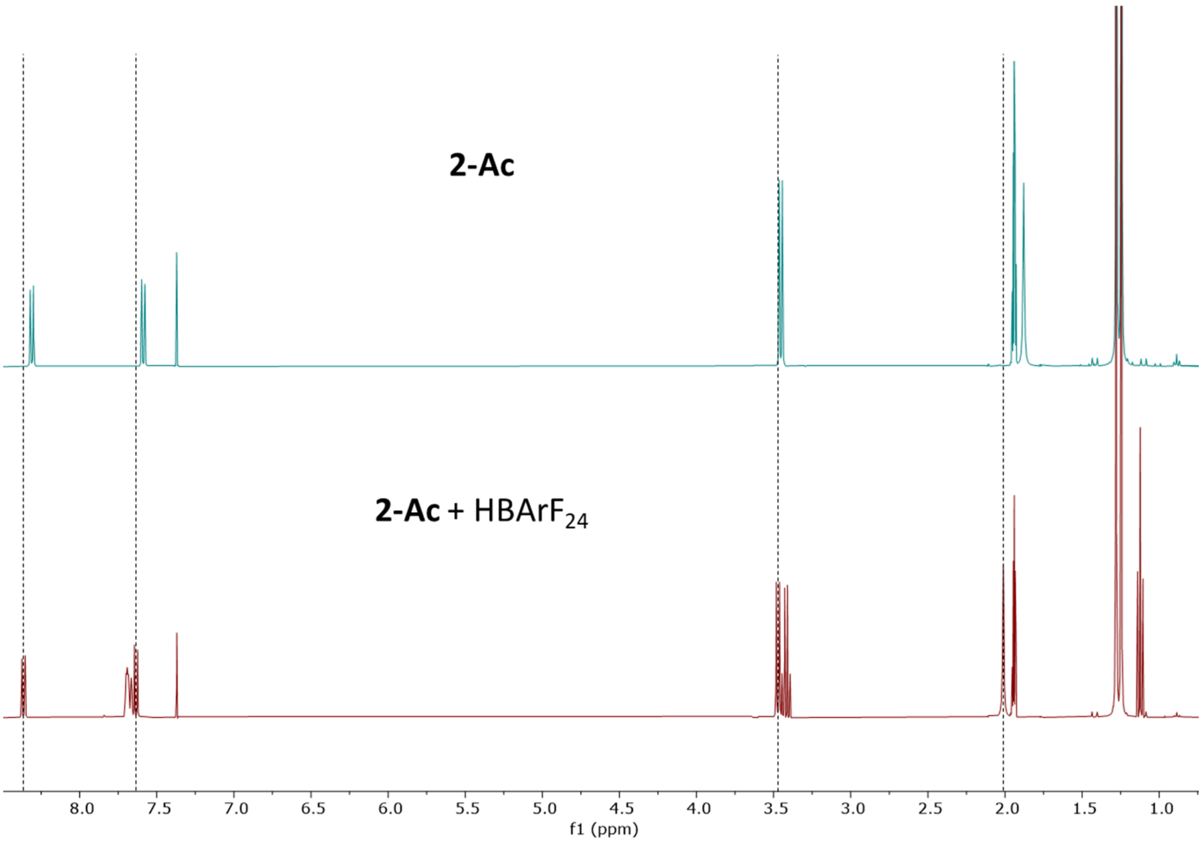

*Figure S16: ^{1}H NMR (MeCN-d_3, 298 K) of **2-Ac** before (top) and after (bottom) $HBArF_{24}$ addition.*

Reactions of 2-Ac with DBU

1 eq of DBU

Complex **2-Ac** (6.6 mg, 9.5 µmol, 1 eq) was dissolved in C_6D_6 and DBU (1.4 µl, 9.4 µmol, 1 eq) was added. The ^{1}H NMR spectrum shows two strongly broadened peaks in the aromatic region which are shifted slightly downfield with respect to the naphthyridine signals in **2-Ac**. The ^{31}P NMR spectrum showed mainly one resonance broadened and slightly shifted downfield from **2-Ac**. Moreover, two new small broad resonances indicative for an asymmetric species were also observed. We interpreted this as that most of the compound is still **2-Ac**, but that its resonances are shifted and broadened due to the proton exchange equilibrium with DBU and its deprotonated analogue (**2C-Ac**), which is the asymmetric species that was observed.

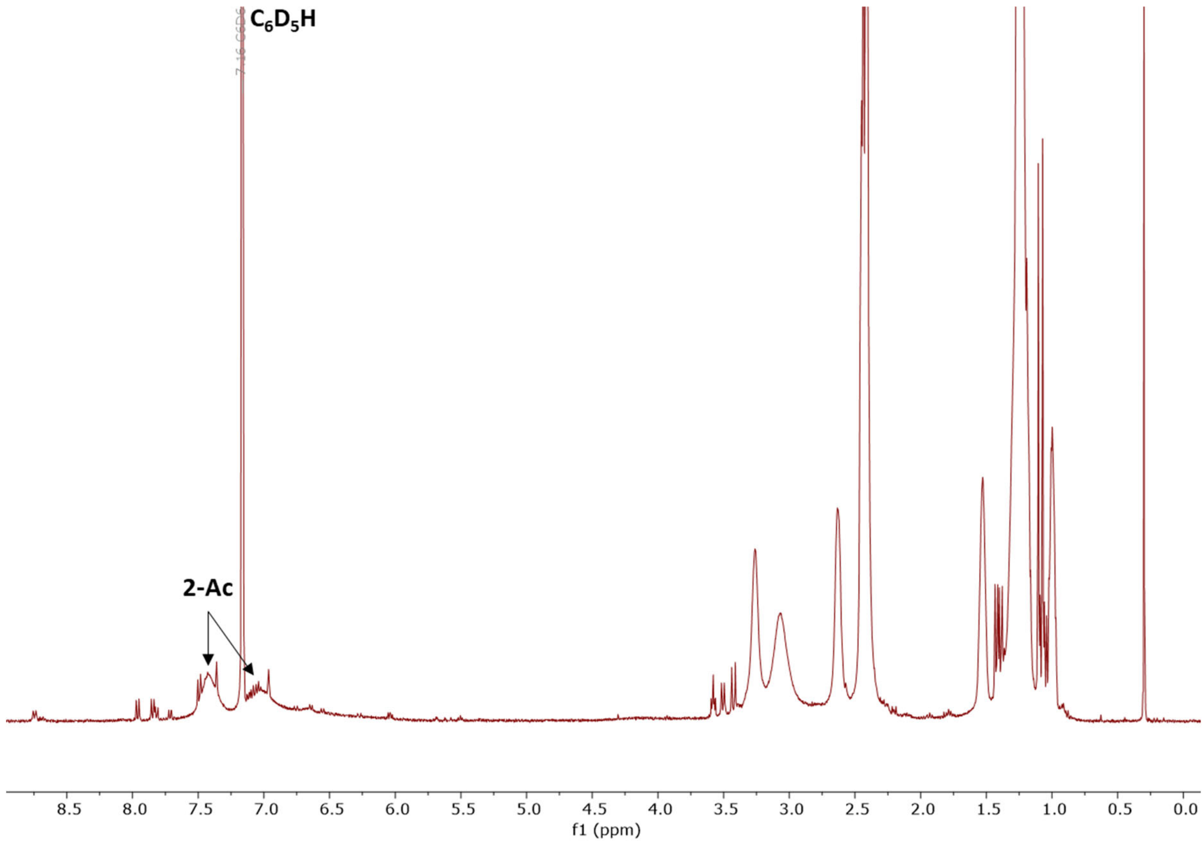

*Figure S17: ^{1}H NMR (C_6D_6, 298 K) of **2-Ac** after addition of 1 eq of DBU.*

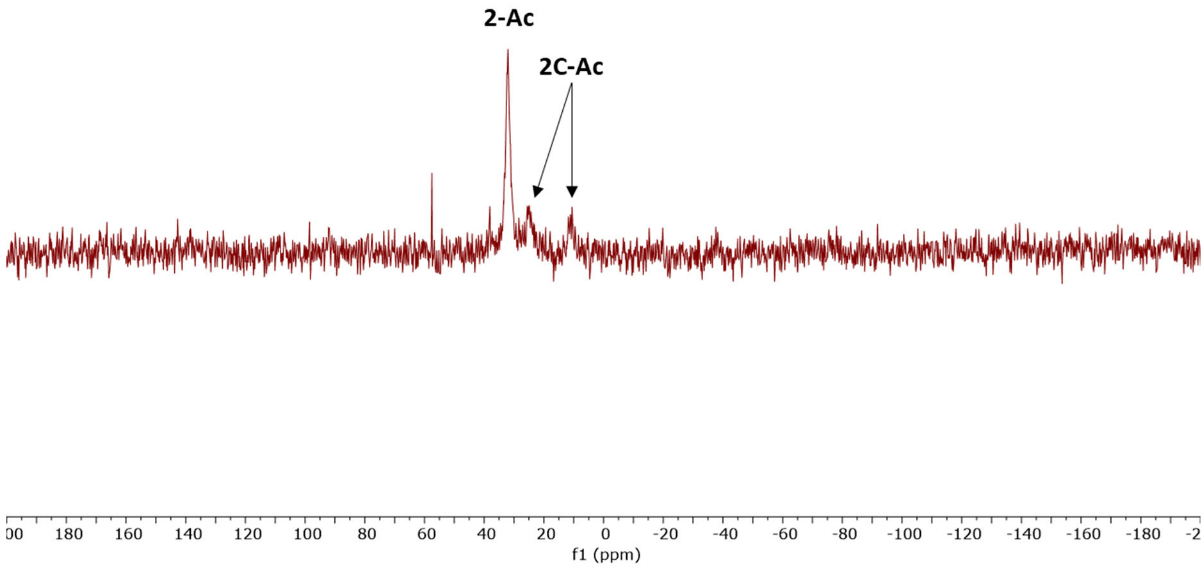

*Figure S18: ^{31}P NMR (C_6D_6, 298 K) of **2-Ac** after addition of 1 eq of DBU.*

10 eq of DBU

Complex **2-Ac** (4.5 mg, 6.5 µmol, 1 eq) was dissolved in THF yielding a brown solution. To this solution, DBU (10 µl, 67 µmol, 10 eq) was added upon which the color changed to red/orange. The ^{1}H NMR spectrum shows broadened peaks consistent with the presence of a symmetrical and an asymmetrical species. The set of peaks associated with the symmetrical species are shifted downfield from the original peak positions of **2-Ac**. Analogous to the reaction with 1 eq of DBU, we interpret this as the resonances of **2-Ac** being broadened and shifted due to the proton exchange with **2C-Ac** and DBU. The ^{31}P NMR spectra are also consistent with this assignment but with 10 eq of DBU, the ratio between **2-Ac** and **2C-Ac** is 0.45 to 1. Taking this ratio together with the concentration of DBU and its reported pKa of 16.6,[13] we calculated the pKa of the methylene linkers of **2-Ac** is 18.1.

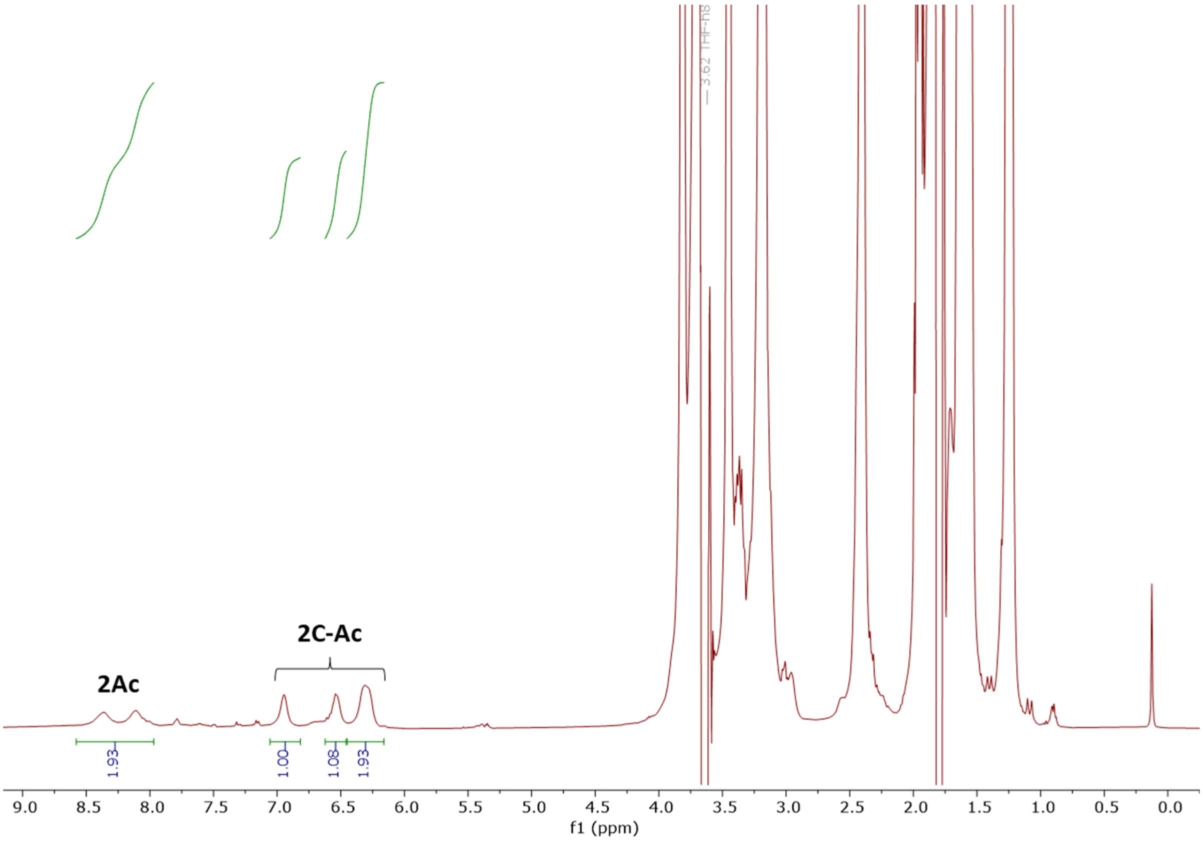

*Figure S19: ^{1}H NMR (THF-h_8, 298 K) of **2-Ac** after addition of 10 eq of DBU with WET solvent suppression.*

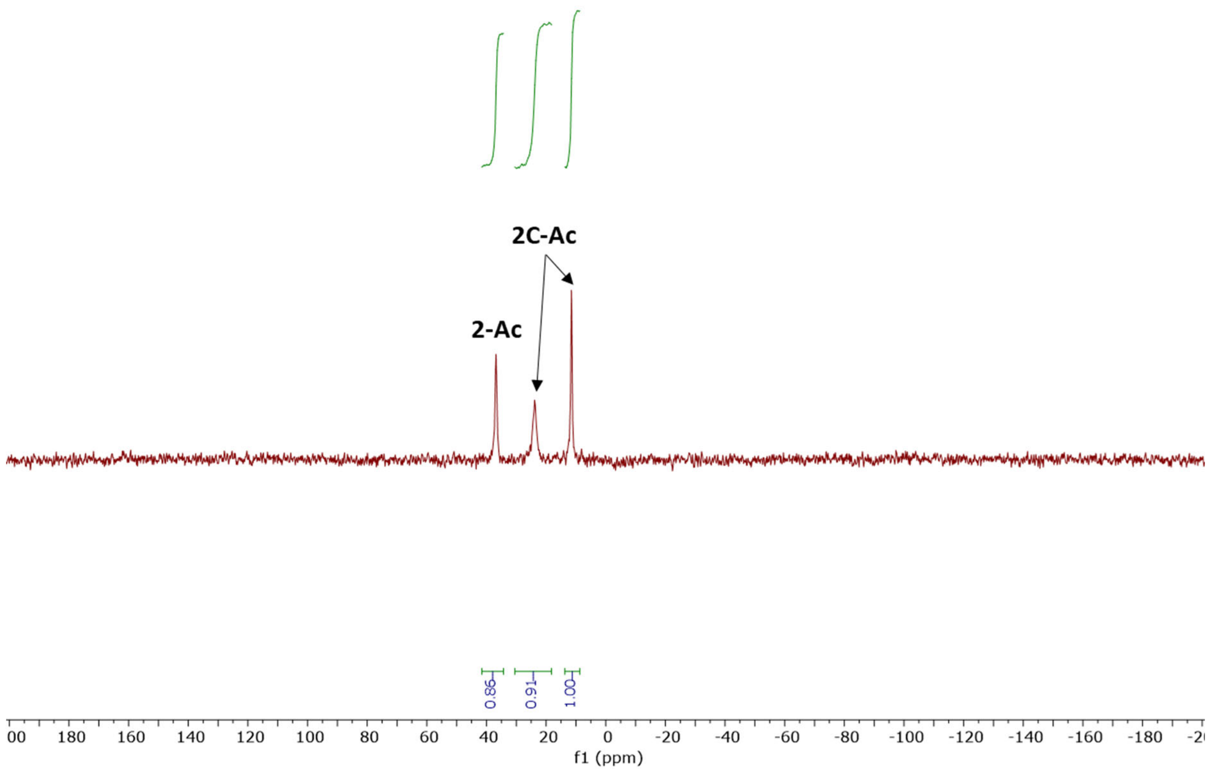

*Figure S20: ^{31}P NMR (THF-h_8, 298 K) of **2-Ac** after addition of 10 eq of DBU.*

Reaction of 1 with 2eq of acetic acid

Complex **1** (5.6 mg, 4.9 µmol, 1 eq) was dissolved in C_6D_6 (0.7 ml) and glacial acetic acid (0.5 µl, 8.7 µmol, 2eq) was added. The ^{1}H NMR spectrum shows signals of both complex **1** as well as **2-Ac**. Extra acetic acid (0.5 µl, 8.7 µmol, 2eq) was added leading to full conversion of **1**, and only **2-Ac** was observed by ^{1}H NMR analysis. This experiment indicates that the intermediates between **1** and **2-Ac** are higher in energy then either of these, which makes it impossible to observe them. We reason that the same likely holds true for the conversion of **1** into **2** with FA.

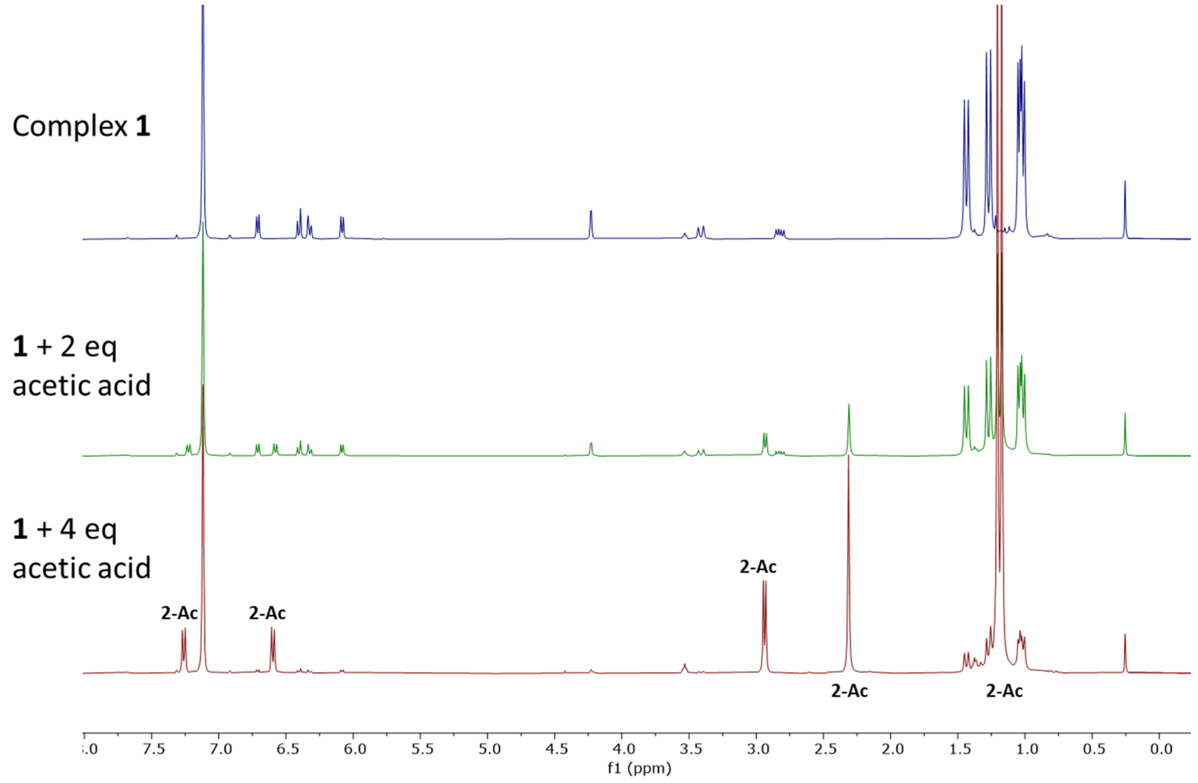

*Figure S21: The stack of ^{1}H NMR spectra (C_6D_6, 298 K) of complex **1**, as well as **1** with 2 eq of acetic acid and with 4 eq of acetic acid added.*

FA dehydrogenation catalysis exploration

FA dehydrogenation with **1** in an NMR tube

Complex **1** (5.0 mg, 4.4 µmol, 1eq) and trimethoxybenzene (internal standard, 0.5 mg, 3.0 µmol, 0.7 eq) were dissolved in THF (0.7 ml) forming a red solution. Formic acid (1.5 µl, 40 µmol, 9 eq) was added after which the color changed from red to brown and eventually a yellow solution with an orange precipitate was formed. The mixture was heated at 50°C and after 3.5 hours the solution has become red again, but there is still precipitate present. The sample was heated at 50°C for 3 days after which a homogeneous red solution is obtained.

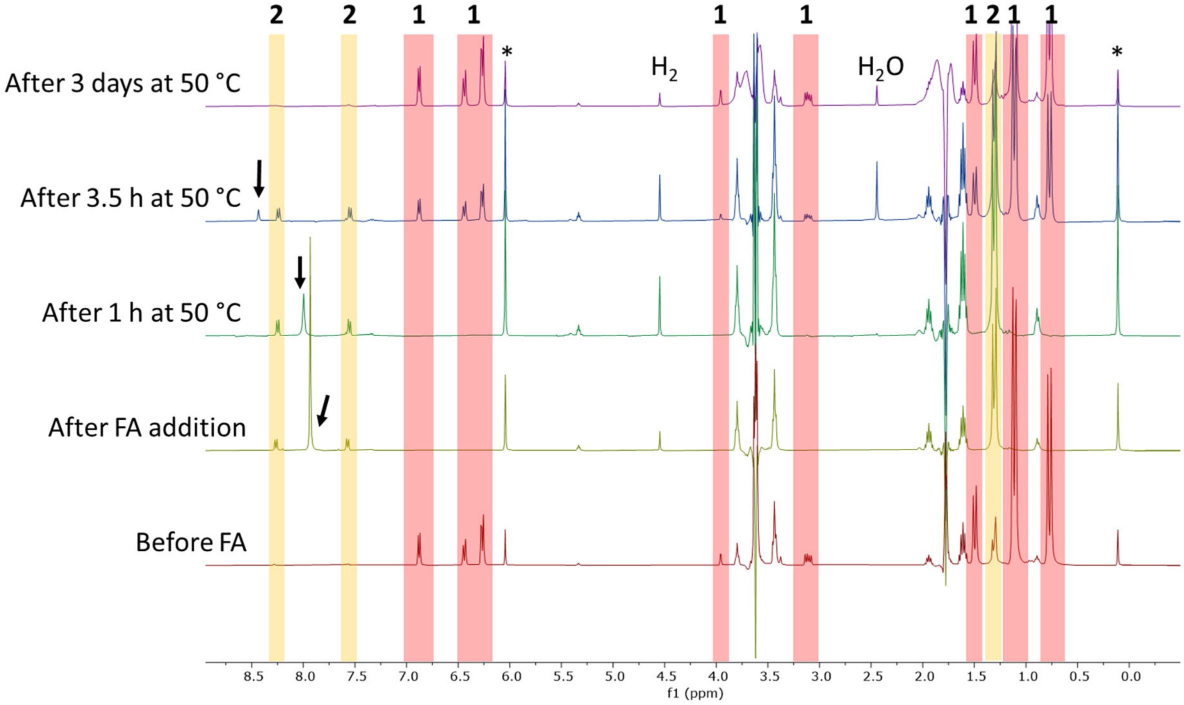

*Figure S22: The stack of ^1H NMR spectra (THF-D_8, 298 K) with PRESAT solvent suppression, of complex **1** with 9 eq of FA added. Before each measurement, excess H_2 pressure was released. The H_2O in the spectrum originates from the FA, however, this is not visible due to fast exchange with the FA OH peak until the excess FA has reacted. * denotes grease and the trimethoxy benzene standard.*

Comparison between THF and MeCN as solvents

To both a solution of **1** (2.5 mg, 2.2 µmol, 1eq) and trimethoxy benezene (internal standard, 11.7 mg, 65 µmol, 30 eq) in THF (0.7 ml), and a solution of **1** (2.4 mg, 2.1 µmol, 1eq) and trimethoxy benezene (internal standard, 11.7 mg, 65 µmol, 30 eq) in MeCN (0.7 ml), formic acid (2 µl, 44 µmol, 20 eq) was added simultaneously. The mixtures were both heated at 50°C for 1 hour after which a ^1H NMR spectrum was recorded. Integration of the formate signal with respect to the internal standard showed no significant conversion in MeCN, while in THF this signal had decreased by 58%.

FA-d_2 dehydrogenation with **1** in an NMR tube

Complex **1** (3.0 mg, 2.6 µmol, 1eq) was dissolved in THF-d_8 (0.6 ml) forming a red solution. Formic acid-d_2 (1.0 µl, 25 µmol, 10 eq) was added after which the color changed from red to brown and eventually a yellow solution with an orange precipitate was formed. The mixture was heated at 50°C for 15 hours after which the solution has become red again, but there is still precipitate present. ^{1}H NMR analysis shows diminished intensities for the methyne and methylene linkers of **1** after the reaction. Comparing the integrals for the signals of the linkers (3.95 and 3.10 ppm*) with the aromatic signals (6.87, 6.44 and 6.26 ppm) of **1** shows ~87% deuteration of the linkers, consistent with the amount of FA-d_2 that was added with respect to the complex.

*The other resonance of the methylene linker in **1** overlaps with the ^{13}C satellite of the solvent and is hence not used for quantification.

Formic acid dehydrogenation kinetics

General procedure

0.6 ml of a stock solution of complex **1** (5.0 mg, 4.4 µmol) in THF (20 ml) was added to a J-Young NMR tube in the glovebox. This sealed tube was consecutively attached to a Schlenk line and cooled to 0°C in an NMR tube adaptor containing degassed silicon oil with a height equal to or higher than the sample liquid in the NMR tube. After cooling, the cap was removed and with a degassed micro syringe, the appropriate amount of a 10%$_{V/V}$ stock solution of formic acid in THF was added after which the tube was quickly sealed and cooled in an ice bath. On ice, the sample was transported to the GC for H_2 gas within 5 min. There it was connected (Figure S23) and heated up to 50°C in a preheated oil bath. After connecting the tube, the GC setup was flushed with N_2 (10 ml/min) for 50 seconds after which the flow was reduced to 1ml/min and a blank was measured. After another minute, the J-Young tube was opened to the system and the measurements were started. The measurement is continued until the amount of H_2 produced drops under the detection limit.

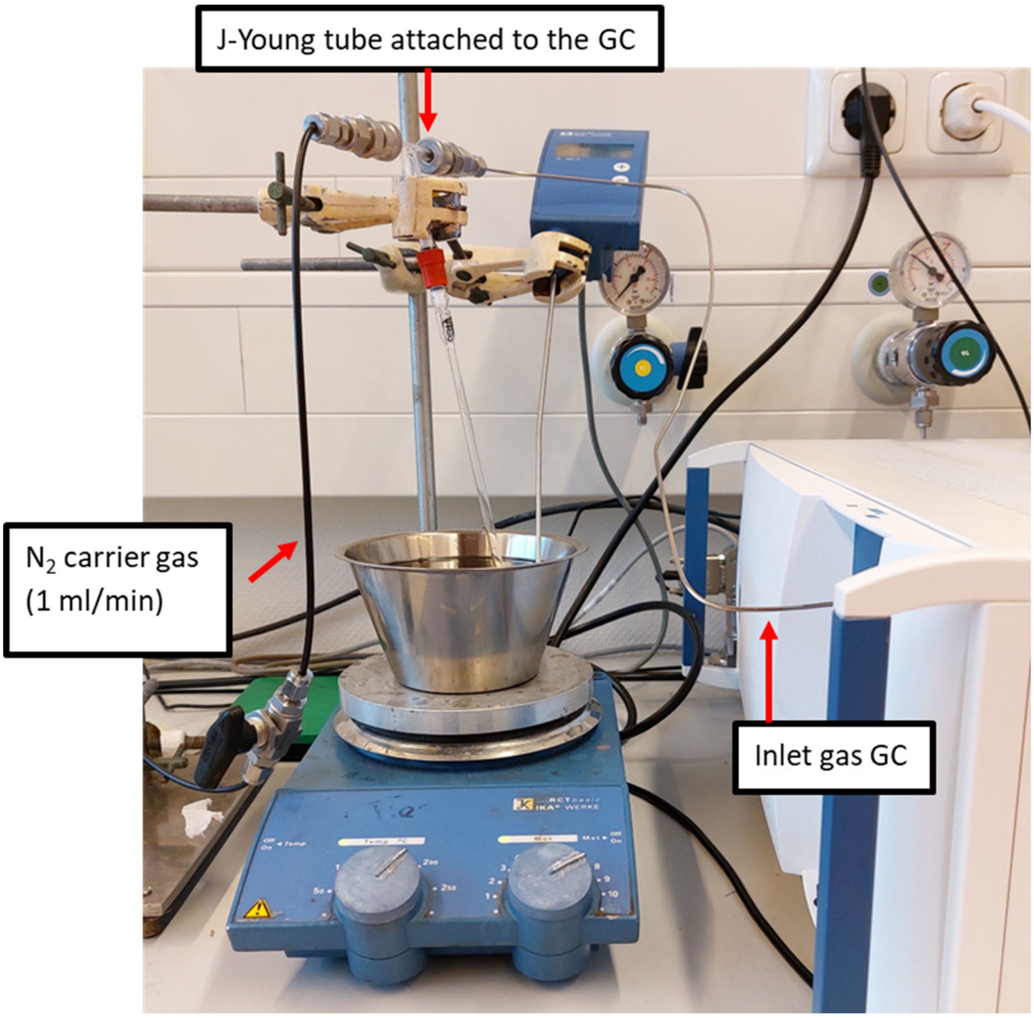

Figure S23: The online GC-setup for H_2 detection that was used for the FA dehydrogenation experiments.

Rate law determination

Order in formic acid

To determine the order in formic acid, the standard procedure for dehydrogenation was used. The amounts of formic acid with respect to **1** that were used for this were: 101 eq (2x), 203 eq, 350 eq (2x), 365 eq, 507 eq, 1014 eq and 1257 eq. The rate was taken as the average rate (mmol/min) over the first 40 minutes of the reaction. To correct for the different consumption of FA in these different reactions, the average FA concentration over the first 40 minutes was used for the Ln([FA]) points on the x-axis of Figure 3a.

Order in catalyst

To determine the order in the catalyst, the standard procedure for dehydrogenation was used albeit with different concentrations of **1** (0.05 µmol, 0.11 µmol, 0.22 µmol, 0.33 µmol and 0.44 µmol). For each reaction 18 µl of the FA stock solution was used. The rate was taken as the average rate (mmol/min) over the first 40 minutes of the reaction. It should be noted that we add **1** as a (pre)catalyst and also use that concentration to calculate the order in the catalyst, however, we observe **2** as resting state of the catalyst during the reaction. This does,

however, not influence the observed order in catalyst but it would change the value of k in the rate law by a factor 2, if **2** would be considered as catalyst.

Kinetic isotope effect experiments

For the kinetic isotope effect measurements, the standard FA dehydrogenation protocol was followed with 10%$_{V/V}$ stock solutions of the different deuterated forms of formic acid. The values for the amounts of hydrogen that were measured with the GC were corrected using the thermal conductivity of D_2 gas for DCOOD or HD gas for DCOOH and HCOOD according to the method reported by Whisnant et al..[14] As an approximation of the thermal conductivity of HD gas, the average of the thermal conductivity of H_2 and D_2 gas was used.[14] To determine the KIE, the average rate over the 38 minutes after 0.005 mmol conversion was taken in each experiment to ensure the same concentration of FA in every experiment. The rate for each formic acid variation was averaged over the three repeat experiments. These average rates were then used to calculate the KIE values, yielding two pairs of KIEs for the C–H and O–H bonds, which were again averaged to obtain the reported KIEs.

FA dehydrogenation with protonated and deprotonated analogues of 1

Dicationic [PNNPCu2H]$_2$

A stock solution (0.22 mM) of the protonated analogue of **1** [PNNPCu$_2$H]$_2$(BArF$_{24}$)$_2$ (5.3 mg, 2.15 µmol) in THF (8.5 ml) was used. This complex will is fully characterized and will be reported in a separate work.[15] Otherwise, the standard FA dehydrogenation protocol was used with 10 µl FA stock solution.

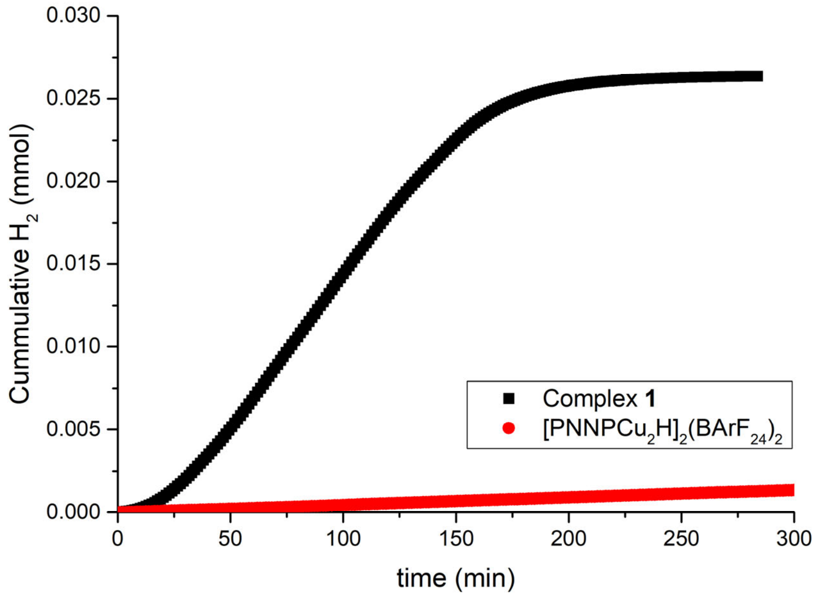

*Figure S24: The comparison of the using complex **1** and using [PNNPCu$_2$H]$_2$(BArF$_{24}$)$_2$ for FA dehydrogenation.*

Anionic PNNP**Cu_2H

A stock solution (0.44 mM) of the previously reported [PNNP**Cu_2H]K18-crown-6 complex[3] (2.2 mg, 2.15 µmol) in THF (5 ml) was used. Otherwise, the standard FA dehydrogenation protocol was used with 18 µl FA stock solution.

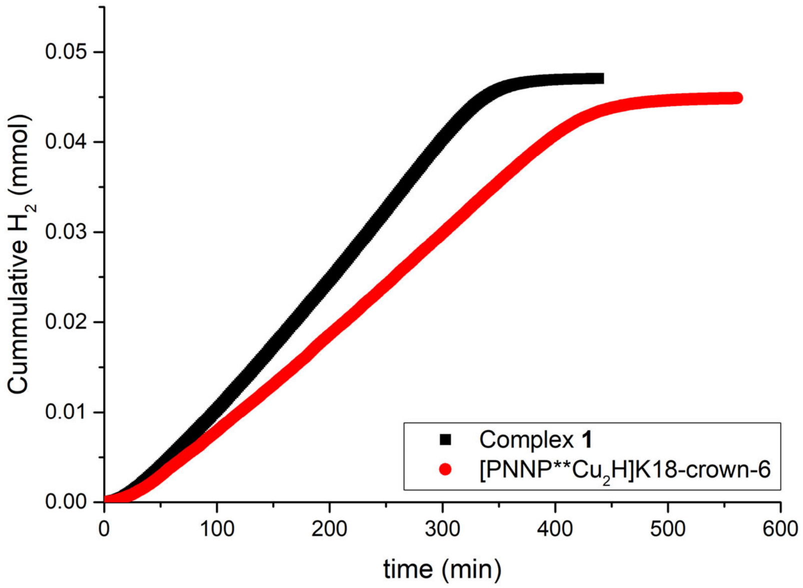

*Figure S25: The comparison of the using complex **1** and using [PNNP**Cu_2H]K18-crown-6[3] for FA dehydrogenation.*

Additive experiments

Triethylamine

To 0.6 ml of the standard stock solution of **1** (1 eq) in THF was added triethylamine (1 µl, 7.2 µmol, 33 eq). After that, the standard FA dehydrogenation protocol was followed and 10 µl of the formic acid stock solution (203 eq) was used.

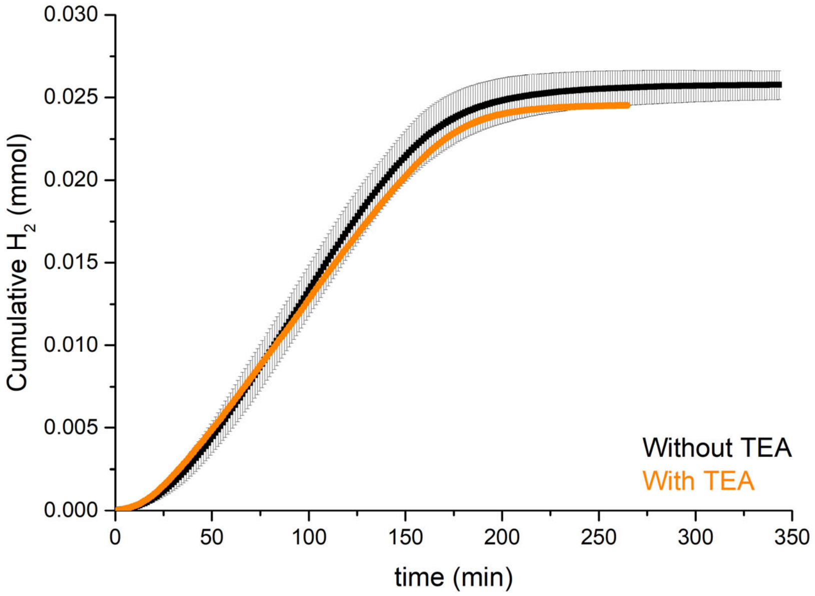

*Figure S26: Comparison of the FA dehydrogenation catalyzed by **1** with and without TEA.*

Water

To 0.6 ml of the standard stock solution of **1** (1 eq) in THF was added degassed water (60 µl, 3.3 mmol, 15000 eq). After that, the standard FA dehydrogenation protocol was followed and 10 µl of the formic acid stock solution (203 eq) was used.

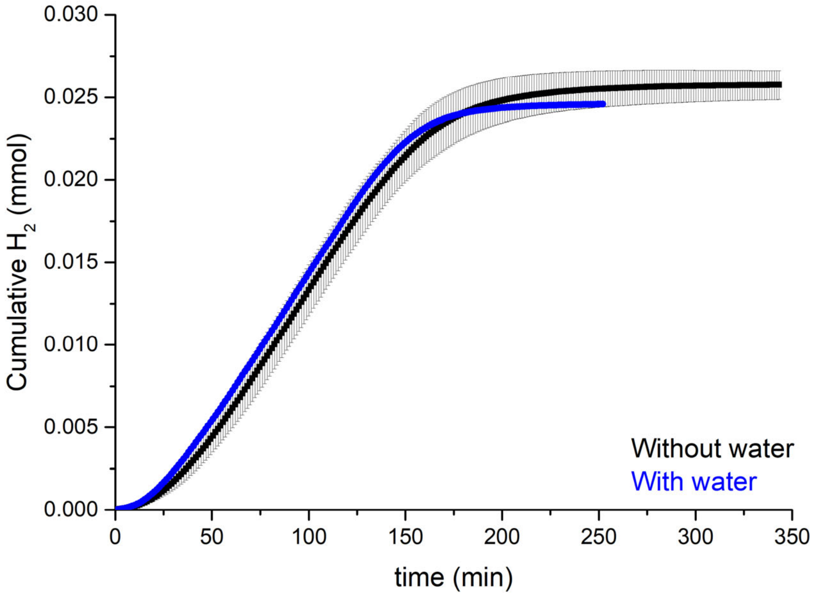

*Figure S27: Comparison of the FA dehydrogenation catalyzed by **1** with and without water.*

DBU

For these experiments, the appropriate amount of DBU was added to the catalyst solution before the formic acid was added. Other than that, the standard FA dehydrogenation protocol with 25 µl FA solution (506 eq) was followed. For the reactions with 0.3 eq and 0.6 eq with respect to formic acid, 10 µl DBU (66.9 µmol) and 5 µl DBU (33.4 µmol) were added respectively. For the experiment with 0.3 eq DBU with respect to the catalyst, a 0.1%$_{V/V}$ stock solution of DBU (10 µl, 0.067 µmol, 0.3 eq) was added.

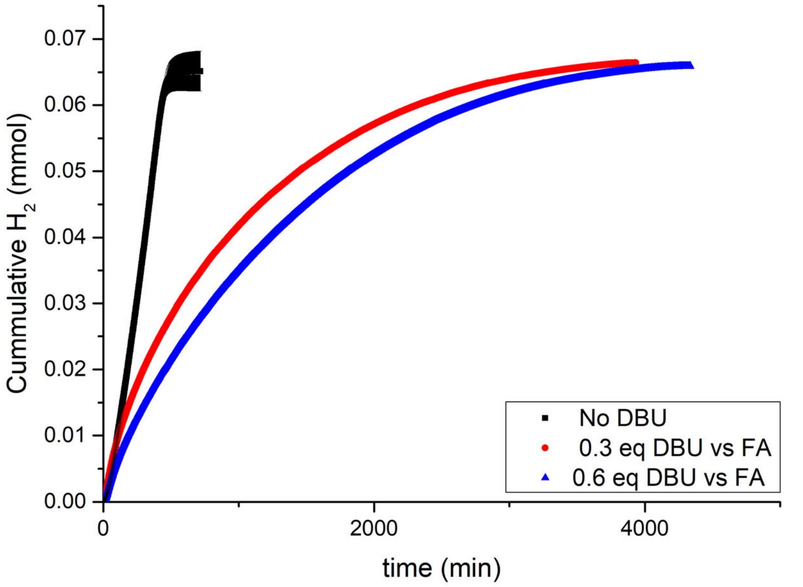

Figure S28: The comparison of the FA dehydrogenation without DBU an with 0.3 eq DBU with respect to FA and 0.6 eq DBU with respect to formic acid.

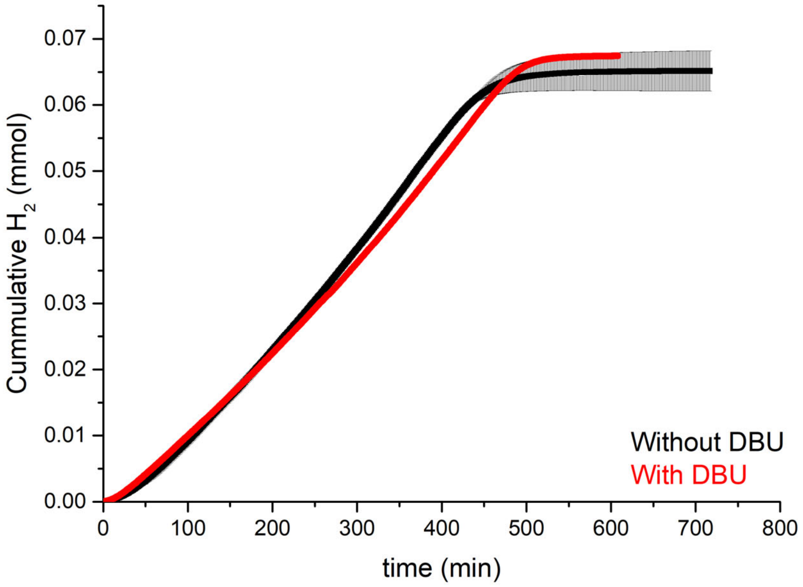

*Figure S29: The comparison of the FA dehydrogenation without DBU an with 0.3 eq DBU with respect to **1**.*

FA dehydrogenation in MeCN

0.6 ml of the standard stock solution of **1** (in THF) was added to a J-Young NMR tube and the solvent was evaporated leaving **1** as a red residue in the required quantity. To this red solid MeCN (0.6 ml) was added yielding a red suspension since **1** is insoluble in MeCN. The rest of the standard protocol was followed and 10 µl (203 eq) of the FA stock solution in THF was used.

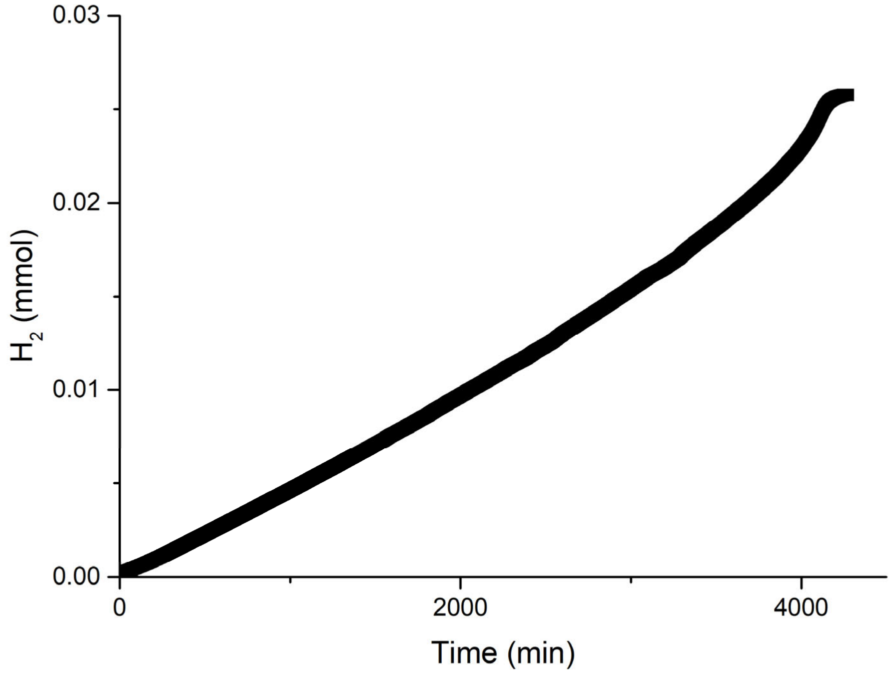

*Figure S30:The H_2 formation over time of FA dehydrogenation catalyzed by **1** in MeCN.*

Rate law modelling

Based on the stoichiometric experiments of **2-Ac** and acids as well as **2-Ac** and deuterated acetic acid, we propose that there are two relevant equilibria with **2** in solution prior to the rate determining step as was discussed in the main text. The observed rate law for the reaction therefore depends on the equilibrium constants of these equilibria. However, since we were unable to determine the equilibrium constants of these experimentally, it is not trivial to determine what rate law should be expected for a proposed mechanism. In turn this prevents the assessment of whether a given proposed mechanism would match with the experimentally observed rate law.

To get an indication of whether a potential mechanism could match the experimental rate law, we modeled the expected rate laws of several mechanisms and probed whether there was a set of K_B and K_C for which this expected rate law would match the experimentally observed rate law.

Rate law modelling with 2B and 2C considered

For this, we considered a bare mechanism with only **2**, **2B**, **2C** and a rate determining step (RDS) (Figure S31). From the acetate exchange of **2-Ac** which involves the methylene linkers as well, we infer that this proceeds through a dissociative mechanism. Based on this, we propose that **2C** is the partially dearomatized dicopper formate complex (Figure S31). For the rate determining step as well as for **2B**, both a dicopper PNNP species or a tetracopper $PNNP_2$ species were considered. To see which of these could match the experimental results that were obtained, the expected rate laws for each combination of these potential forms of **2B** and the RDS were expressed as a function of the two equilibrium constants K_B and K_C(Table S1). The derivation of these rate laws can be found in the section below.

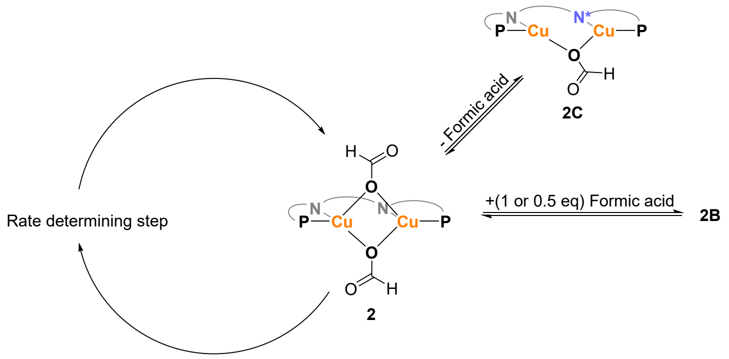

*Figure S31: Bare mechanism that was considered for the expected rate law simulations. **2** and **2C** are drawn schematically and the N* denotes the partially dearomatized backbone in **2C**. The rate determining step as well as **2B** were both considered as being either dimeric or monomeric.*

Table S1: An overview of the theoretical rate laws for the catalytic FA dehydrogenation catalyzed by 1 expressed as functions of equilibrium constants KB and KC. [FA] is the formic acid concentration, [2]i is the initial concentration of 2 (i.e. 2x the concentration of 1).

	**2B** is a $PNNPCu_2$ species	**2B** is a $PNNP_2Cu_4$ species
The RDS has a $PNNPCu_2$ composition	$rate = k[\mathbf{2}] = k\left(\frac{[\mathbf{2}]_i}{1 + K_B[FA] + \frac{K_C}{[FA]}}\right)$	$rate = k[\mathbf{2}] = k\left(\frac{-\left(1+\frac{K_C}{[FA]}\right) + \sqrt{\left(1+\frac{K_C}{[FA]}\right)^2 - 4(2K_B[FA])(-[\mathbf{2}]_i)}}{2(2K_B[FA])}\right)$
The RDS has a $PNNP_2Cu_4$ composition	$rate = k[\mathbf{2}]^2 = k\left(\frac{[\mathbf{2}]_i}{1 + K_B[FA] + \frac{K_C}{[FA]}}\right)^2$	$rate = k[\mathbf{2}]^2 = k\left(\frac{-\left(1+\frac{K_C}{[FA]}\right) + \sqrt{\left(1+\frac{K_C}{[FA]}\right)^2 - 4(2K_B[FA])(-[\mathbf{2}]_i)}}{2(2K_B[FA])}\right)^2$

With the expected rate laws from Table S1, the concentrations for $[\mathbf{2}]_i$ and [FA] that were used for the experimental determination of the rate law (see above) can be filled in to obtain a set of expressions for the rate that depend solely on K_B and K_C. These expressions can then be used to determine the expected orders in [**2**] and [FA] (and with that the expected rate law) in the same way as was done for the experimental rate law (i.e. by plotting the ln of the rate against the ln of [**2**] to obtain the order in catalyst, and by plotting it against the ln of [FA] to obtain the order in FA). To do so, different values for K_B and K_C were filled in by trial-and-error to see which values would give an expected rate laws that matches the experimental rate law.

For the combination of a $PNNPCu_2$ RDS and a $PNNP_2Cu_4$ composition of **2B** (Table S1, top right), as well as for the combination of a $PNNP_2Cu_4$ RDS and a $PNNPCu_2$ composition of **2B** (Table S1, bottom left), no set of K_B and K_C was found for which the theoretical rate law reproduced the experimental results. For the other two theoretical rate laws, there was a set of K_B and K_C which reproduced the experimental results reasonably well (Figure S32). This indicates that both the combination of a $PNNPCu_2$ RDS and a $PNNPCu_2$ composition of **2B**, as well as a combination of a $PNNP_2Cu_4$ RDS and a $PNNP_2Cu_4$ composition of **2B**, match the experimentally observed rate law. Based on the kinetics experiments, these two possibilities cannot be distinguished.

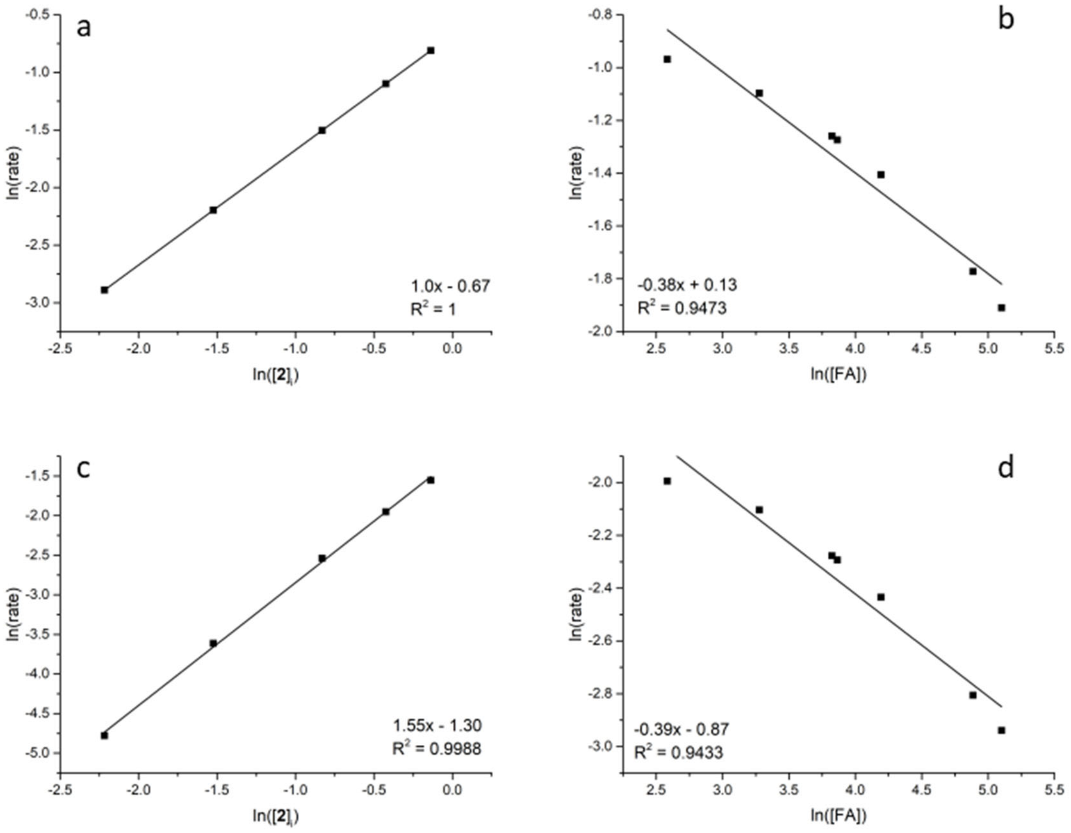

*Figure S32: a) The ln of the calculated rate plotted against the initial catalyst concentration [**2**]$_I$ for the case with PNNPCu$_2$ composition for both the RDS and **2B** (K_B = 0.012, K_C = 0.000001). b) The ln of the calculated rate plotted against the formic acid concentration for the case with PNNPCu$_2$ composition for both the RDS and **2B** (K_B = 0.012, K_C = 0.000001). c) The ln of the calculated rate plotted against the initial catalyst concentration [**2**]$_I$ for the case with PNNP$_2$Cu$_4$ composition for both the RDS and **2B** (K_B = 0.012, K_C = 1). d) The ln of the calculated rate plotted against the formic acid concentration for the case with PNNP$_2$Cu$_4$ composition for both the RDS and **2B** (K_B = 0.012, K_C = 1).*

Rate law modelling with only 2B considered

Since the results above indicate that K_C approximates zero in the case of a monomolecular RDS and **2B**, and since the experimental results suggest that this equilibrium is not relevant in the absence of base due to the vast excess of acid, we also performed the rate law modelling only considering the equilibrium between **2** and **2B**. This means that we consider the general mechanism shown in Figure S33, which leads to the rate laws shown in table 2. Modelling the expected rate laws provides very similar results compared to when K_C was taken into account, although the expected rate law for the bimolecular RDS and **2B** is a bit further from the experimental result (Figure S34).

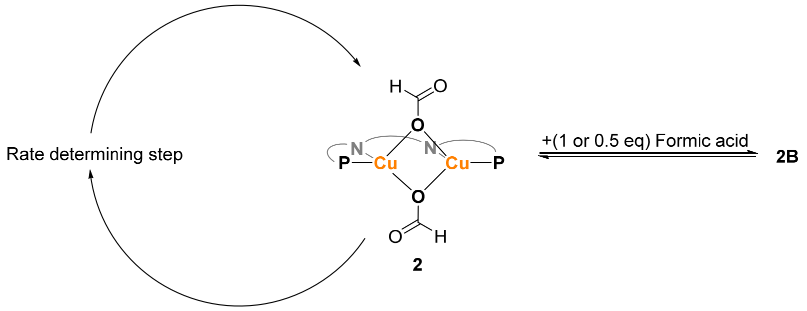

Figure S33: Bare mechanism that was considered for the expected rate law simulations. ***2*** *is drawn schematically. The rate determining step as well as* ***2B*** *were both considered as being either dimeric or monomeric.*

Table S2: An overview of the theoretical rate laws for the catalytic FA dehydrogenation catalyzed by 1 expressed as functions of equilibrium constant K_B. [FA] is the formic acid concentration, $[2]_i$ is the initial concentration of 2 (i.e. 2x the concentration of 1).

	**2B** is a $PNNPCu_2$ species	**2B** is a $PNNP_2Cu_4$ species
The RDS has a $PNNPCu_2$ composition	$rate = k[\mathbf{2}] = k\left(\frac{[\mathbf{2}]_i}{1+K_B[FA]}\right)$	$rate = k[\mathbf{2}] = k\left(\frac{-1+\sqrt{1^2-4(2K_B[FA])(-[\mathbf{2}]_i)}}{2(2K_B[FA])}\right)$
The RDS has a $PNNP_2Cu_4$ composition	$rate = k[\mathbf{2}]^2 = k\left(\frac{[\mathbf{2}]_i}{1+K_B[FA]}\right)^2$	$rate = k[\mathbf{2}]^2 = k\left(\frac{-1+\sqrt{1^2-4(2K_B[FA])(-[\mathbf{2}]_i)}}{2(2K_B[FA])}\right)^2$

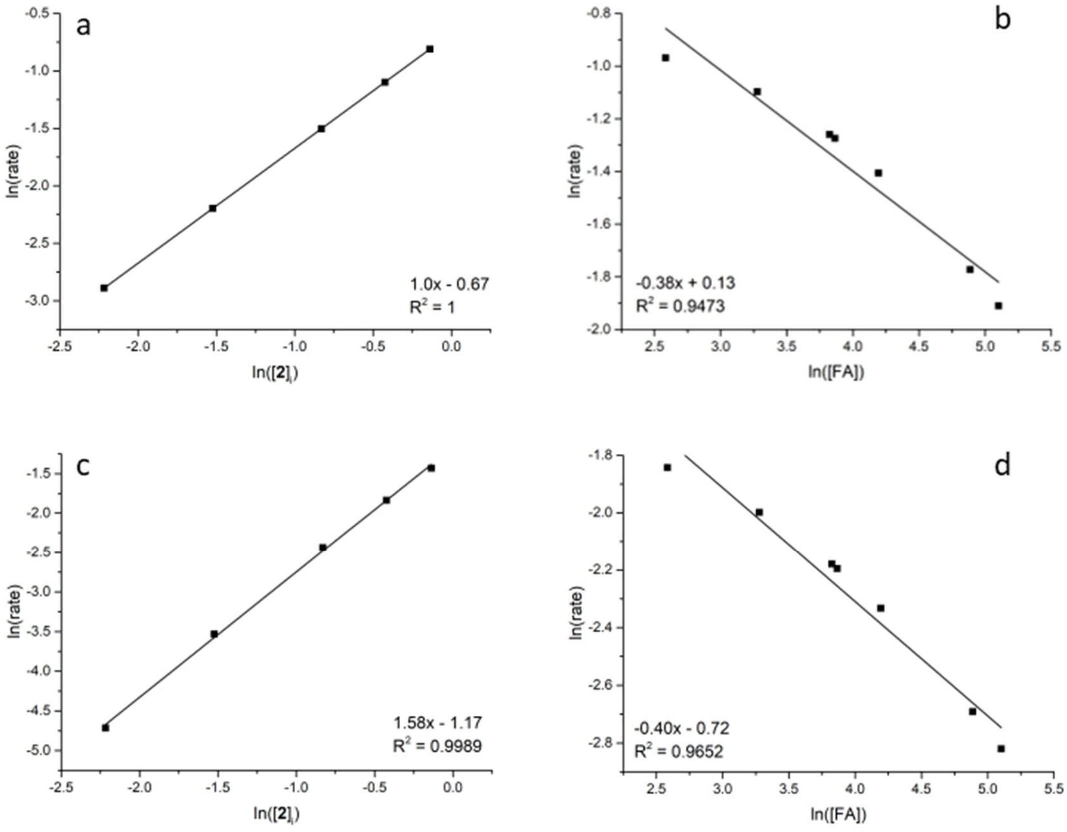

*Figure S34: a) The ln of the calculated rate plotted against the initial catalyst concentration [**2**]$_I$ for the case with $PNNPCu_2$ composition for both the RDS and **2B** (K_B = 0.012). b) The ln of the calculated rate plotted against the formic acid concentration for the case with $PNNPCu_2$ composition for both the RDS and **2B** (K_B = 0.012). c) The ln of the calculated rate plotted against the initial catalyst concentration [**2**]$_I$ for the case with $PNNP_2Cu_4$ composition for both the RDS and **2B** (K_B = 0.010). d) The ln of the calculated rate plotted against the formic acid concentration for the case with $PNNP_2Cu_4$ composition for both the RDS and **2B** (K_B = 0.010).*

Rate law derivation

For the derivation of the rate laws as a function of K_B and K_C, we assume that the only complexes that have a significant concentration in solution are **2**, **2B** and **2C**, in line with the notion that **2** is the resting state of the catalyst and that this is the only complex that is observed during the reaction. This leads to equations 1 and 2, for a monomeric and dimeric **2B** respectively. [**1**]$_i$ is the initial concentration of **1**, [**2**]$_i$ is the initial concentration of **2**, and [**2**], [**2B**] and [**2C**] are the concentrations of **2**, **2B** and **2C** respectively.

$$2[\mathbf{1}]_i = [\mathbf{2}]_i = [\mathbf{2}] + [\mathbf{2B}] + [\mathbf{2C}] \quad (1)$$

$$2[\mathbf{1}]_i = [\mathbf{2}]_i = [\mathbf{2}] + 2[\mathbf{2B}] + [\mathbf{2C}] \quad (2)$$

In case of a RDS of composition $PNNPCu_2$ or $PNNP_2Cu_4$, the rate laws are given in equation 3 and 4 respectively. The equation for the equilibrium between **2** and **2C** is provided in equation 5. For the cases in which **2B** has the composition of $PNNPCu_2$ or $PNNP_2Cu_4$, the equilibrium equations are provided in equation 6 and 7 respectively.

$$rate = k[\mathbf{2}] \quad (3)$$

$$rate = k[\mathbf{2}]^2 \quad (4)$$

$$K_C = \frac{[\mathbf{2C}][FA]}{[\mathbf{2}]} \quad (5)$$

$$K_B = \frac{[\mathbf{2B}]}{[\mathbf{2}][FA]} \quad (6)$$

$$K_B = \frac{[\mathbf{2B}]}{[\mathbf{2}]^2[FA]} \quad (7)$$

Equation 5 can be used to express [**2C**] in terms of K_c, [FA] and [**2**]. Similarly, equation 6 or 7 can be used to express [**2B**] in terms of K_B, [FA] and [**2**]. These expressions for [**2B**] and [**2C**] can then be filled in into equation 1 or 2. In case of **2B** having a $PNNPCu_2$ composition, rearranging the obtained equation will lead to an expression of [**2**] in terms of [**2**]$_i$, [FA], K_B and K_C. Filling in this expression into equations 3 and 4 will provide the two rate laws on the left column of Table S1 top and bottom respectively. In the case of **2B** having a $PNNP_2Cu_4$ composition, the quadratic formula was used to obtain an expression of [**2**] in terms of [**2**]$_i$, [FA], K_B and K_C. Filling in this expression into equations 3 and 4 will provide the two rate laws on the right column of Table S1 top and bottom respectively. For the rate laws in Table S2, the same was done, however, in equations 1 and 2, the + [**2C**] term was left out.

Computational methods

General considerations

Calculations were performed using Gaussian 16 rev. C01 software.[16] The ωB97X-D functional developed by Head-Gordon and coworkers was used.[17] The redefinition of Ahlrichs triple-zeta split valence basis set (def2-TZVP) was used on all atoms unless noted otherwise.[18] All calculations were performed with an SMD implicit solvation model (Tetrahydrofuran) unless mentioned otherwise.[19] All intermediates were confirmed to be stationary points by the absence of imaginary vibrational frequencies and the transition states were confirmed by the presence of a singular imaginary frequency. All calculations were performed at 298.15 K except for the KIE calculations which were calculated at 323.15 K. To calculate the KIE, the frequencies of the structures of **2**, **2'** and **TS1** were calculated with deuterium on both OOC-H positions to obtain the free energy barrier with deuterium. Then using the Arrhenius equation, the ratio between the reaction rates with hydrogen and deuterium was calculated at 323.15 K to obtain the calculated KIE.

The energies plotted for PES scans are the electronic energies referenced to the lowest energy structure in the scan and the energy of this structure is provided with respect to **2**. This means that a thermochemical correction is applied (denoted with a *) to the lowest energy structure to provide its energy relative to **2** as a Gibbs free energy. The energy difference between such a lowest energy structure and the other structures in the PES scan is, however, an electronic energy difference (unless noted otherwise). It should therefore be noted that the graphs bear

the implicit assumption that the thermal correction to the lowest energy structure and the other structures is the same, which is a very crude approximation. The reader should therefore be cautioned that the energies of the structures in the PES with respect to **2** have a somewhat larger error than usual for DFT calculations. However, in most cases the conclusions from the PES scans are not affected by this. In the cases where this approximation may be problematic for the interpretation, the thermally corrected energy is also provided (again denoted with a *).

Structures of intermediates and transition states

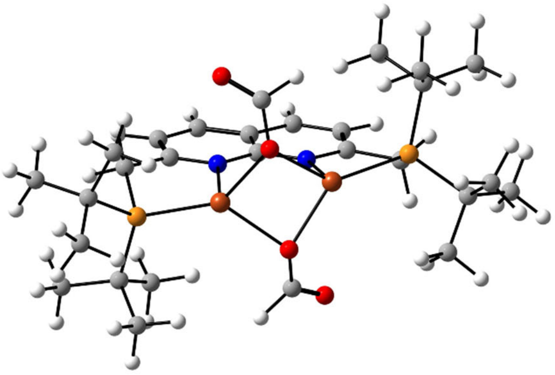

2

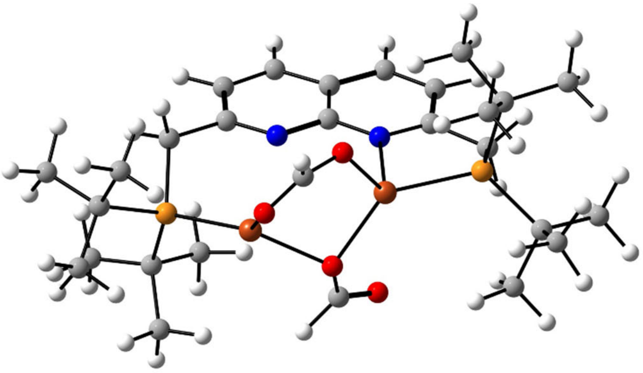

2'

*Figure S35: The optimized structures of **2** and **2'** with relative energies of 0.0 kcal/mol and 0.9 kcal/mol.*

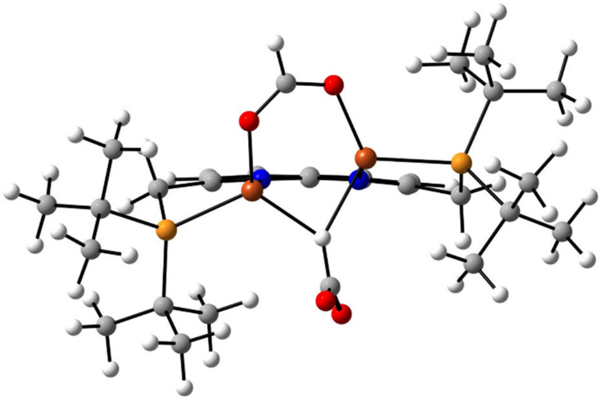

Int A

*Figure S36: The optimized structure of **Int A** with a relative energy of 14.4 kcal/mol.*

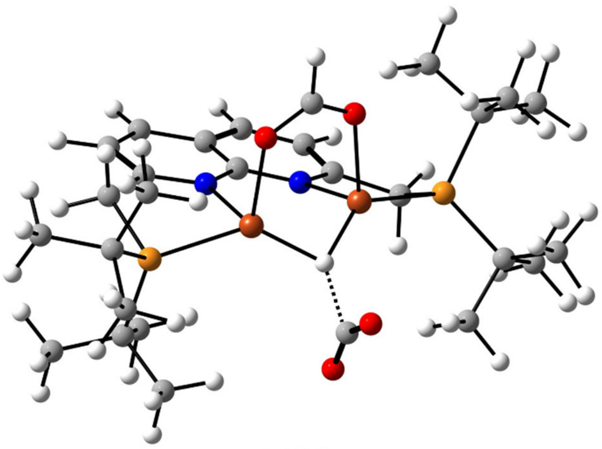

TS1

*Figure S37: The optimized structure of **TS1** with a relative energy of 16.0 kcal/mol. The dotted C–H bond indicates the orientation of the negative vibration at -503 cm^{-1}.*

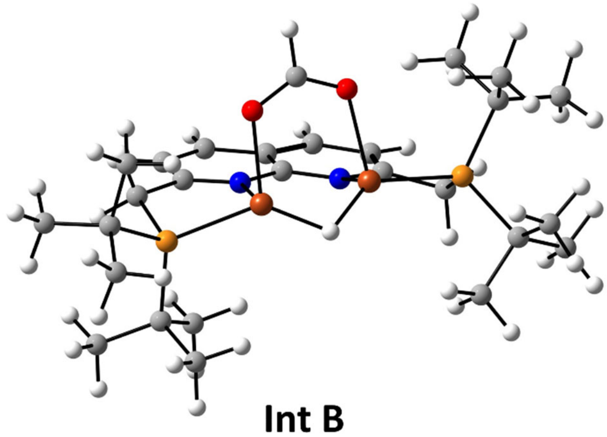

Int B

*Figure S38: The optimized structure of **Int B** with a relative energy of 1.1 kcal/mol.*

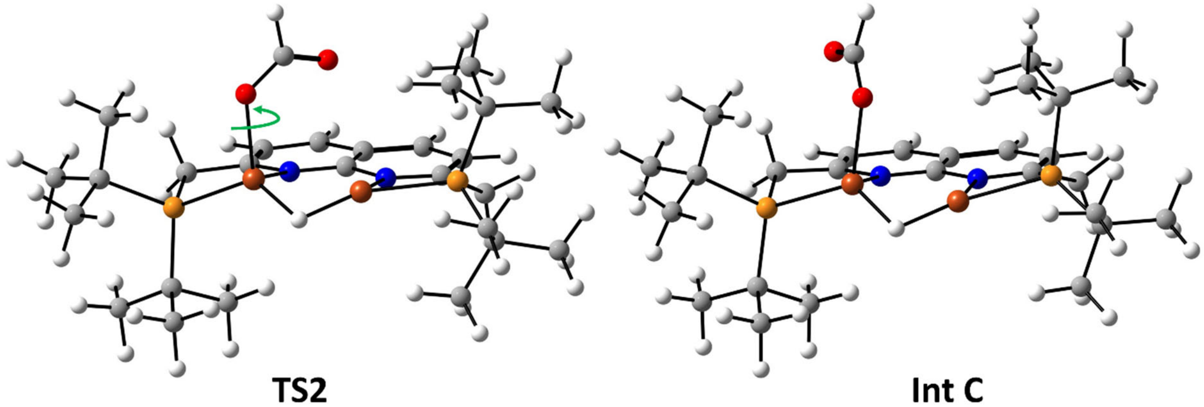

TS2 **Int C**

*Figure S39: The optimized structures of **TS2** and **Int C** with relative energies of 5.6 kcal/mol and 2.0 kcal/mol. The green arrow indicates the negative vibration at -89 cm^{-1}.*

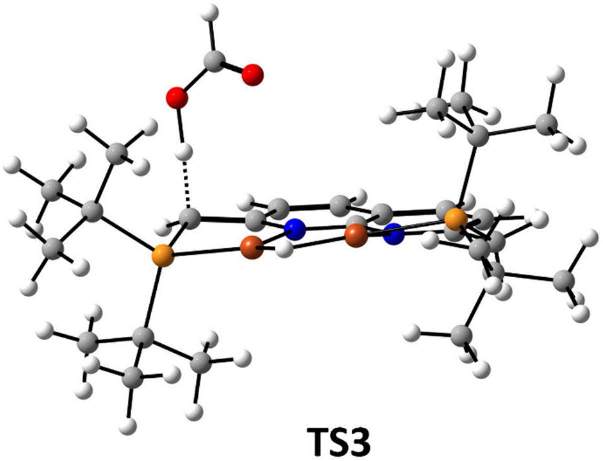

TS3

*Figure S40: The optimized structure of **TS3** with a relative energy of 15.3 kcal/mol. The dotted C–H bond indicates the orientation of the negative vibration at -1316 cm^{-1}.*

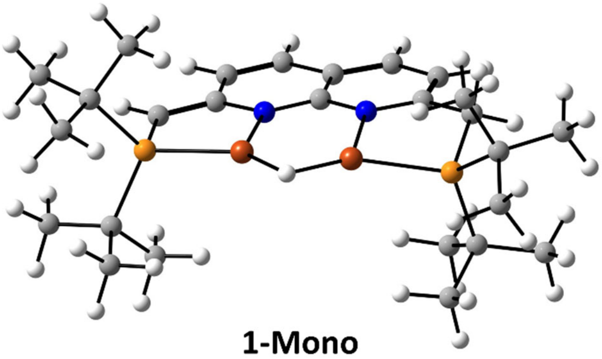

1-Mono

*Figure S41: The optimized structure of **1-mono** with a relative energy of 7.7 kcal/mol.*

Potential energy surface scan from 2 to Int A

To evaluate the feasibility of the formation of **Int A** a PES scan was performed in which the Cu–H bond in **Int A** was gradually lengthened (Figure S42). This scan was performed at ωB97XD/def2SVP level of theory with SMD (THF) solvent correction. Unfortunately, we were not able to obtain a converged structure of one of the steps of the PES scan with the def2TZVP basis set and hence used the lower level of theory results in this case.

Attempts to perform a PES scan from **2** to **Int A** were unsuccessful likely for practical reasons since the tumbling of the formate is a rather complex motion that is uphill in energy. Therefore these attempts all yielded more stable structures in which one of the oxygen atoms was still bound to a Cu center. In the PES scan of **Int A** to **2** it is evident that when the Cu–H distance is sufficiently long to allow for O–Cu coordination, that the energy drops suddenly.

After the main tumbling in which the first O–Cu bond is formed, there have to be some minor rearrangements of the formate ligands to form **2** or **2'**. Based on the experimentally observed functionality of the formate ligands, it seems reasonable to assume that such rearrangements should be feasible and facile compared to RDS of the reaction.

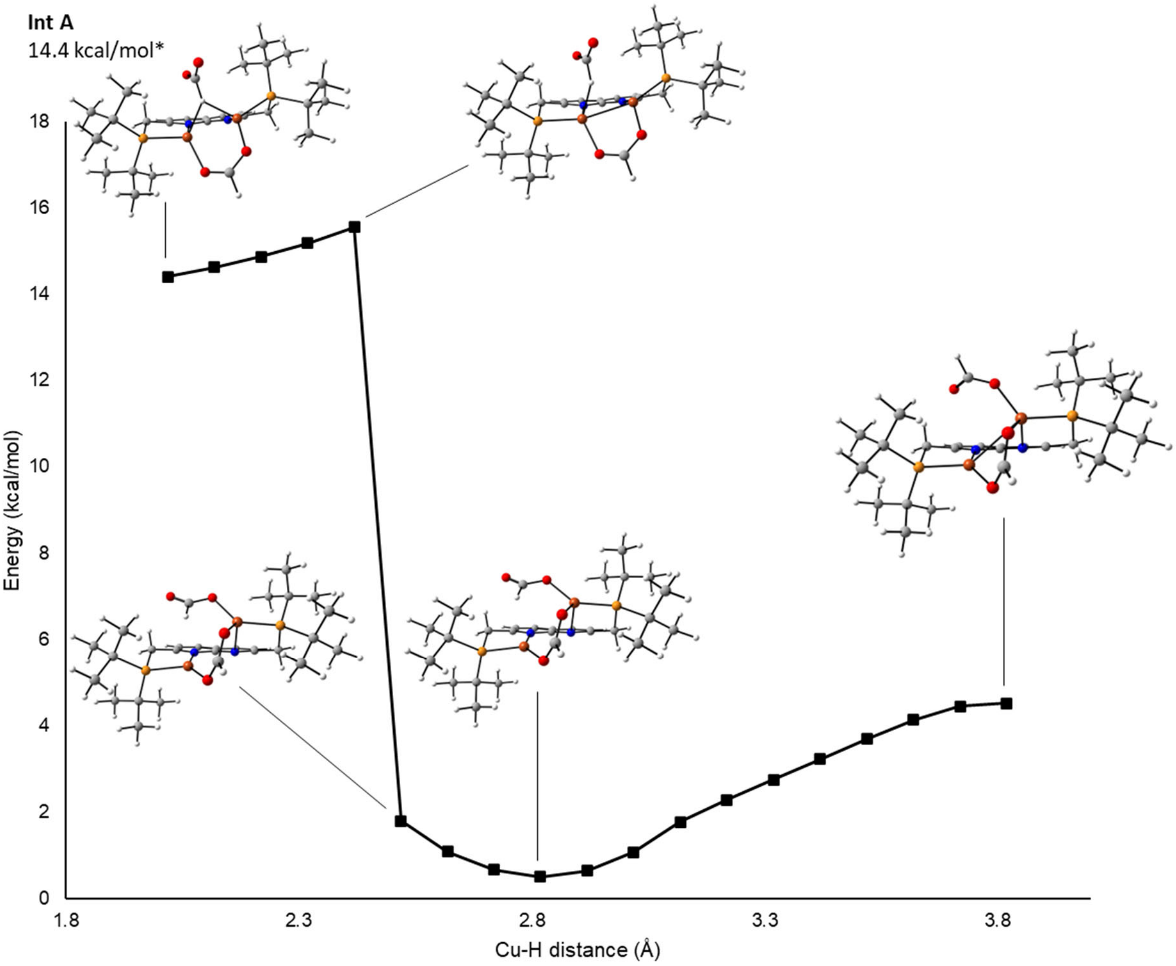

*Figure S42: Potential energy surface scan of the formate tumble going from **2** to **Int A**. Calculated at ωB97XD/def2SVP level of theory (no solvent model). The electronic energies in the PES scan are normalized to the energy of **Int A** relative to **2**. The * denotes that this energy includes a thermochemical correction.*

Potential energy surface scan formate dissociation from Int C

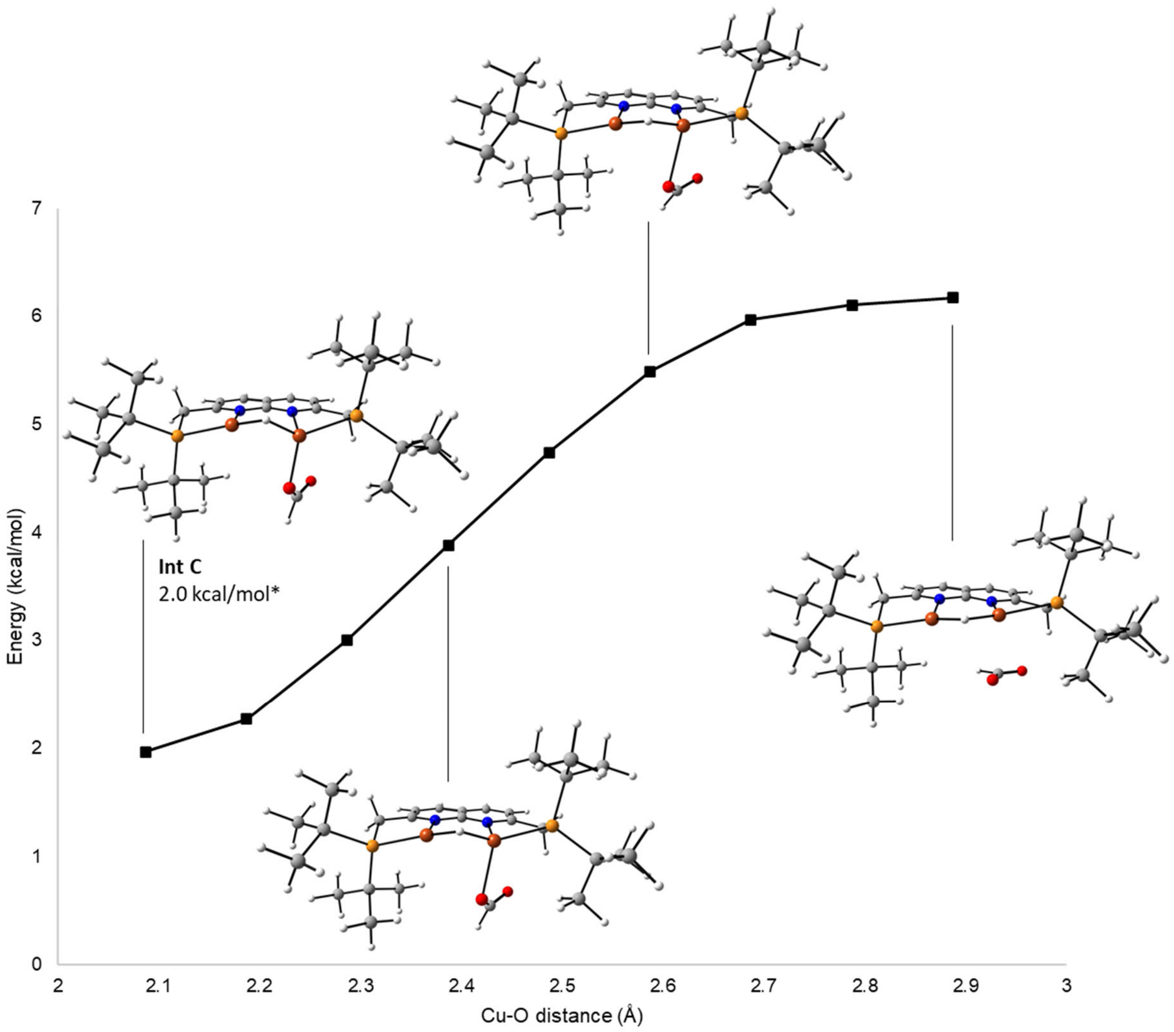

Figure S43: Potential energy surface scan of the formate dissociation from ***Int C*** *calculated at ωB97XD/def2TZVP level of theory with SMD solvent correction (THF). The electronic energies in the PES scan are normalized to the energy of* ***Int C*** *relative to* ***2****. The * denotes that this energy includes a thermochemical correction.*

Potential energy surface scan concerted backbone deprotonation

Based on the structures from the PES scan in Figure S44, we attempted to optimize **TS3’**. However, any optimization attempt would yield either **TS3** (see Figure 5 and Scheme 3) or **Int C**. Since the energy of **TS3’** in the PES scan is relatively close to that of **TS3**, we calculated the frequencies of the highest energy structure in the PES scan to obtain the thermochemically corrected energy of **TS3’** (17.6 kcal/mol). Note that the PES scan is performed along the reaction coordinate (the O-H bond which should be formed) here and that therefore the structure of **TS3’** we used is optimized in every dimension but the reaction pathway, making the vibrational analysis still valid[20] even though it is not an optimized geometry in a global sense.

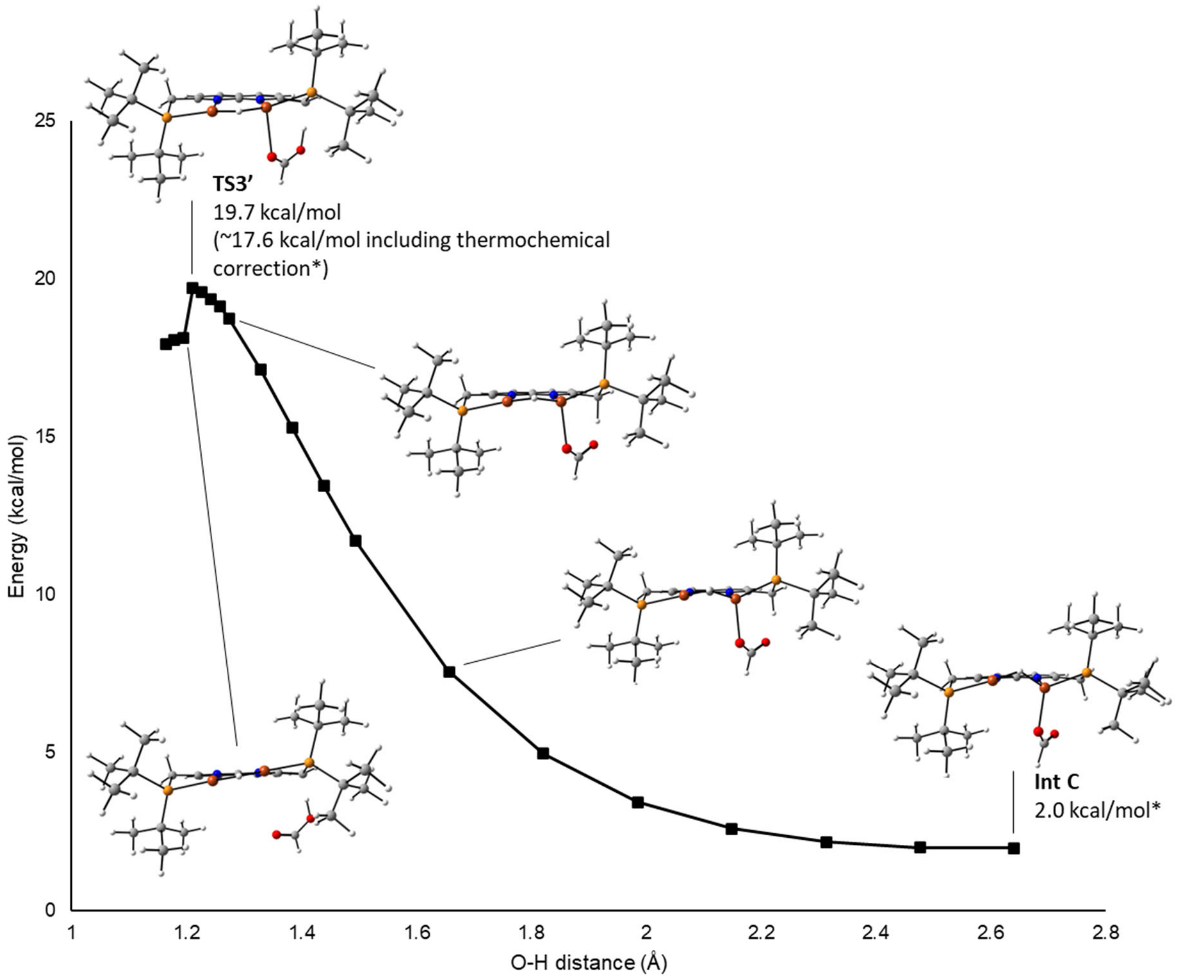

*Figure S44: Potential energy surface scan for the intramolecular MLC methylene deprotonation. Calculated at ωB97XD/def2TZVP level of theory with SMD solvent correction (THF). The electronic energies in the PES scan are normalized to the energy of **Int C** relative to **2**. The * denotes that this energy includes a thermochemical correction.*

Potential energy surface scan MLC H_2 formation

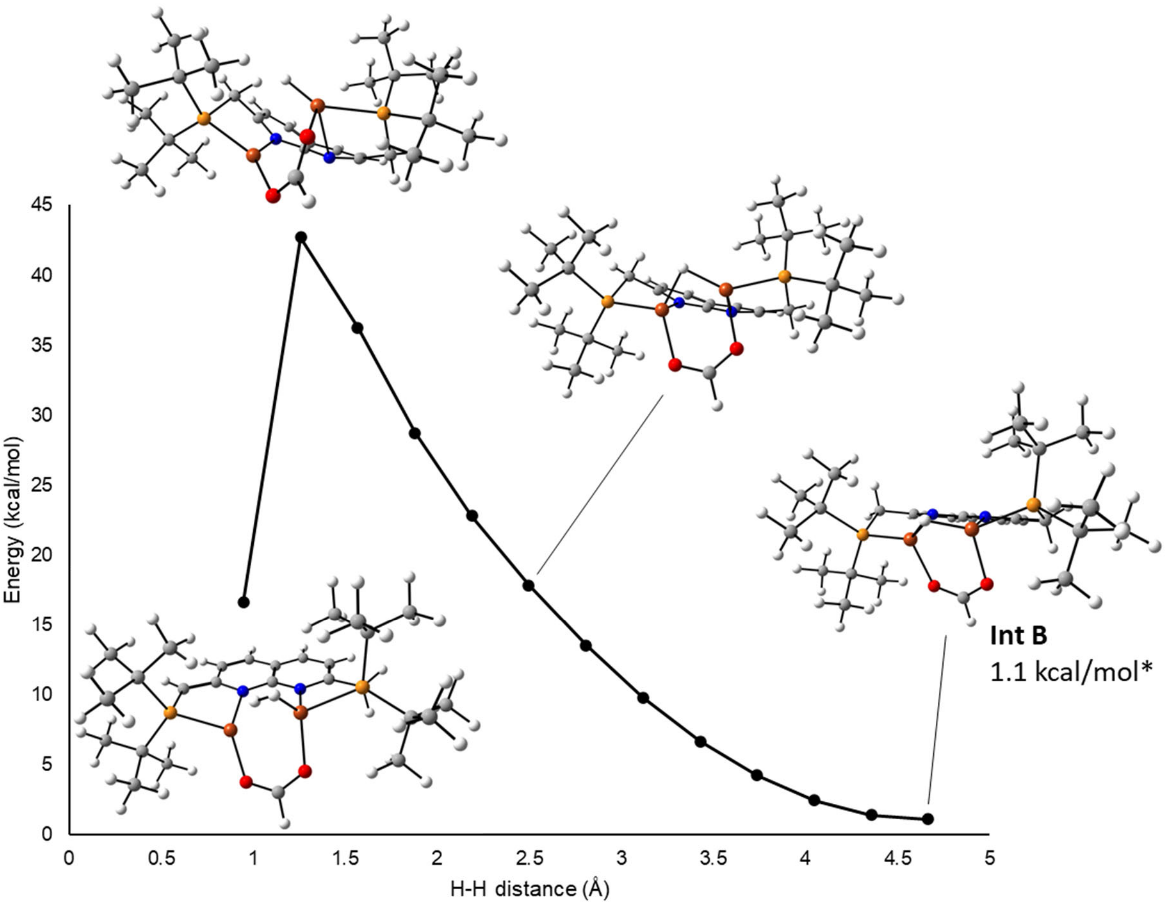

*Figure S45: Potential energy surface scan of the proposed MLC H_2 release pathway. Calculated at ωB97XD/def2SVP level of theory (no solvent model). The electronic energies in the PES scan are normalized to the energy of **Int B** relative to **2**. The * denotes that this energy includes a thermochemical correction.*

Potential energy surface scan β-hydride elimination

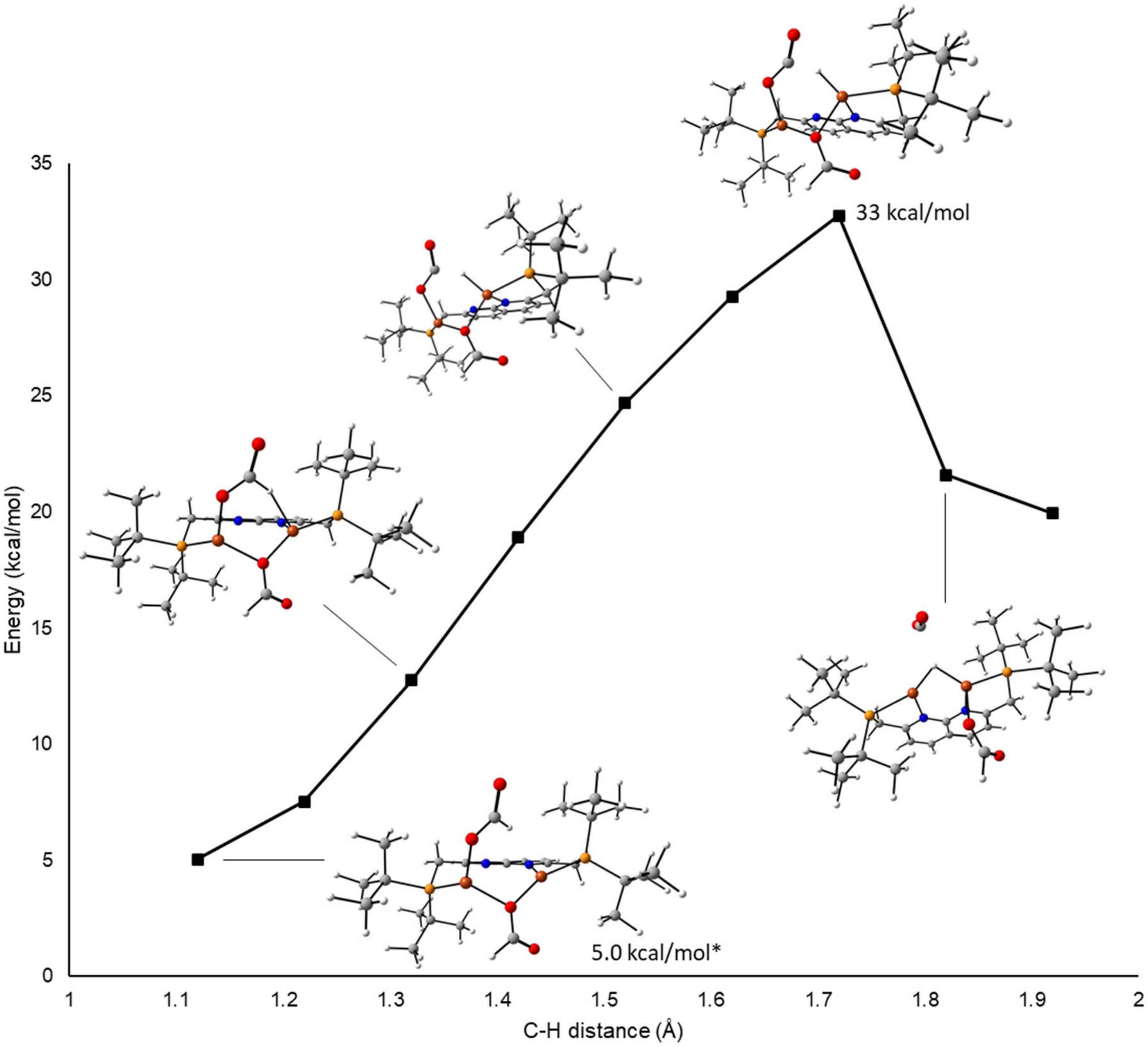

*Figure S46: Potential energy surface scan of the alternative C–H cleavage pathway via β-hydride elimination. Calculated at ωB97XD/def2TZVP level of theory with SMD solvent correction (THF). The electronic energies in the PES scan are normalized to the energy of the lowest intermediate relative to **2**. The * denotes that this energy includes a thermochemical correction.*

Estimating the reaction rate at room temperature without excess FA

The DFT calculations only take into account a single molecule of **2** and no excess of FA, however, in our experiments there is always an excess of FA which influences the reaction rate. In order to assess whether the calculated barrier of 16.0 kcal/mol (at room temperature) is reasonable for the experimentally observed rate, we need to translate the observed rate at 50 °C with excess FA to what the rate would be under the conditions taken into account in the DFT calculations. To do so we used the reaction rates (in triplo) for FA-h_2 from the experiments that were also used for the KIE determinations (see above). These rates are averages over 38 minutes, so the average FA concentration over that same time period was taken for each repeat of the experiment (i.e. in triplo). Using the rate law that was determined (see main text, equation 1), the rate constant k was determined, which was then used to find the reaction rate at 0.88 mM FA concentration (4 eq FA v.s. **1**). This provided an average

corrected rate over the 3 experiments of 0.72 mmol/min (~2.7 turnovers/min). Using the Arrhenius equation with the calculated barrier of 16.0 kcal/mol indicates that at 25 °C, the reaction should be 8.1 times slower than at 50 °C which leads to a TOF at 25 °C of 0.3 min^{-1}. This means that one turnover takes 3 minutes at 25 °C, which indicates to us that the calculated barrier of 16.0 kcal/mol is reasonable.

X-ray crystal structure of 2

$C_{28}H_{46}Cu_2N_2O_4P_2$, Fw = 663.69, red needle, 0.52 × 0.07 × 0.07 mm^3, orthorhombic, $P2_12_12$ (no. 18), a = 18.8987(10), b = 18.9349(5), c = 8.8746(3) Å, V = 3175.7(2) $Å^3$, Z = 4, D_x = 1.388 g/cm^3, μ = 2.87 mm^{-1}. The diffraction experiment was performed on a Bruker Proteum diffractometer with rotating anode and Helios optics (λ = 1.54184 Å) at a temperature of 250(2) K up to a resolution of $(\sin \theta/\lambda)_{max}$ = 0.59 $Å^{-1}$. The Eval14 software[21] was used for the intensity integration. A multi-scan absorption correction and scaling was performed with SADABS[22] (correction range 0.39-0.75). A total of 25174 reflections was measured, 5546 reflections were unique (R_{int} = 0.060), 5307 reflections were observed [$I>2\sigma(I)$]. The structure was solved with Patterson superposition methods using SHELXT.[23] Structure refinement was performed with SHELXL-2019[24] on F^2 of all reflections. Pseudo-tetragonal twinning was included in the refinement with a twofold rotation about *hkl*=(1,1,0) as twin operation. Additional inversion twinning could not be refined because of large correlations in the least-squares matrix. Non-hydrogen atoms were refined freely with anisotropic displacement parameters. The structure contains two independent molecules which are both located on twofold rotation axes. Both molecules show whole-molecule disorder. Hydrogen atoms were introduced in calculated positions and refined with a riding model. 441 Parameters were refined with 1504 restraints (geometry of the disordered molecules; displacement parameters of all non-hydrogen atoms except copper). R1/wR2 [$I > 2\sigma(I)$]: 0.0920 / 0.2595. R1/wR2 [all refl.]: 0.0946 / 0.2658. S = 1.057. Twin fraction BASF = 0.302(4). Residual electron density between -0.43 and 1.10 e/$Å^3$. Geometry calculations and checking for higher symmetry was performed with the PLATON program.[25]

CCDC 2332075 contains the supplementary crystallographic data for this paper. These data can be obtained free of charge from The Cambridge Crystallographic Data Centre via www.ccdc.cam.ac.uk/data_request/cif.

References

(1) Kounalis, E.; Lutz, M.; Broere, D. L. J. Cooperative H $_2$ Activation on Dicopper(I) Facilitated by Reversible Dearomatization of an “Expanded PNNP Pincer” Ligand. *Chemistry – A European Journal* **2019**, *25* (58), 13280–13284. https://doi.org/10.1002/chem.201903724.

(2) Kounalis, E.; Lutz, M.; Broere, D. L. J. Tuning the Bonding of a μ-Mesityl Ligand on Dicopper(I) through a Proton-Responsive Expanded PNNP Pincer Ligand. *Organometallics* **2020**, *39* (4), 585–592. https://doi.org/10.1021/acs.organomet.9b00829.

(3) Bienenmann, R. L. M.; Schanz, A. J.; Ooms, P. L.; Lutz, M.; Broere, D. L. J. A Well-Defined Anionic Dicopper(I) Monohydride Complex That Reacts like a Cluster. *Angewandte Chemie International Edition* **2022**, *61* (29), e202202318. https://doi.org/10.1002/anie.202202318.

(4) Brookhart, M.; Grant, B.; Volpe, A. F. $[(3,5\text{-}(CF_3)_2C_6H_3)_4B]^-[H(OEt_2)_2]^+$: A Convenient Reagent for Generation and Stabilization of Cationic, Highly Electrophilic Organometallic Complexes. *Organometallics* **1992**, *11* (11), 3920–3922. https://doi.org/10.1021/om00059a071.

(5) Fulmer, G. R.; Miller, A. J. M.; Sherden, N. H.; Gottlieb, H. E.; Nudelman, A.; Stoltz, B. M.; Bercaw, J. E.; Goldberg, K. I. NMR Chemical Shifts of Trace Impurities: Common Laboratory Solvents, Organics, and Gases in Deuterated Solvents Relevant to the Organometallic Chemist. *Organometallics* **2010**, *29* (9), 2176–2179. https://doi.org/10.1021/om100106e.

(6) Bianchini, C.; Ghilardi, C. A.; Meli, A.; Midollini, S.; Orlandini, A. Facile Reduction of Carbon Dioxide, Carbonyl Sulfide, and Carbon Disulfide by Copper(I) Borohydride. X-Ray Crystal Structure of the Complex $[(Triphos)Cu(O_2CH)]$. *J Organomet Chem* **1983**, *248* (2), c13–c14. https://doi.org/10.1016/0022-328X(83)85031-1.

(7) Phung, K.; Thuéry, P.; Berthet, J.-C.; Cantat, T. $CO_2/^{13}CO_2$ Dynamic Exchange in the Formate Complex $[(2,9\text{-}(^tBu)_2\text{-Phen})Cu(O_2CH)]$ and Its Catalytic Activity in the Dehydrogenation of Formic Acid. *Organometallics* **2023**. https://doi.org/10.1021/acs.organomet.3c00302.

(8) Costes, J. P.; Dahan, F.; Laurent, J. P. A Further Example of a Dinuclear Copper(II) Complex Involving Monoatomic Acetate Bridges. Synthesis, Crystal Structure, and Spectroscopic and Magnetic Properties of Bis(.Mu.-Acetato)Bis(7-Amino-4-Methyl-5-Aza-3-Hepten-2-Onato(1-))Dicopper(II). *Inorg Chem* **1985**, *24* (7), 1018–1022. https://doi.org/10.1021/ic00201a011.

(9) Shi, W.-J.; Hou, L.; Li, D.; Yin, Y.-G. Supramolecular Assembly Driven by Hydrogen-Bonding and π–π Stacking Interactions Based on Copper(II)-Terpyridyl Complexes. *Inorganica Chim Acta* **2007**, *360* (2), 588–598. https://doi.org/10.1016/j.ica.2006.08.004.

(10) Pauly, M. A.; Erwin, E. M.; Powell, D. R.; Rowe, G. T.; Yang, L. A Synthetic, Spectroscopic and Computational Study of Copper(II) Complexes Supported by Pyridylamide Ligands. *Polyhedron* **2015**, *102*, 722–734. https://doi.org/10.1016/j.poly.2015.11.015.

(11) Fernandes, C.; Neves, A.; Bortoluzzi, A. J.; Mangrich, A. S.; Rentschler, E.; Szpoganicz, B.; Schwingel, E. A New Dinuclear Unsymmetric Copper(II) Complex as Model for the Active Site of Catechol Oxidase. *Inorganica Chim Acta* **2001**, *320* (1–2), 12–21. https://doi.org/10.1016/S0020-1693(01)00470-4.

(12) Hakimi, M.; Rezaei, H.; Moeini, K.; Mardani, Z.; Eigner, V.; Dušek, M. Formation of a Copper–Copper Bond in Coordination of a Cyclotriphosphazene Ligand toward Cu(II): Structural, Spectral and Docking Studies. *J Mol Struct* **2020**, *1207*, 127804. https://doi.org/10.1016/j.molstruc.2020.127804.

(13) Kütt, A.; Selberg, S.; Kaljurand, I.; Tshepelevitsh, S.; Heering, A.; Darnell, A.; Kaupmees, K.; Piirsalu, M.; Leito, I. PKa Values in Organic Chemistry – Making Maximum Use of the Available Data. *Tetrahedron Lett* **2018**, *59* (42), 3738–3748. https://doi.org/10.1016/j.tetlet.2018.08.054.

(14) Whisnant, C. S.; Hansen, P. A.; Kelley, T. D. Measuring the Relative Concentration of H_2 and D_2 in HD Gas with Gas Chromatography. *Review of Scientific Instruments* **2011**, *82* (2). https://doi.org/10.1063/1.3531974.

(15) Bienenmann, R. L. M.; Thangsrikeattigun, C.; Schanz, A. J.; Lutz, M.; Baik, M-H.; Broere, D. L. J.. *Manuscript in preparation*.

(16) Gaussian 16, Revision C.01, M. J. Frisch, G. W. Trucks, H. B. Schlegel, G. E. Scuseria, M. A. Robb, J. R. Cheeseman, G. Scalmani, V. Barone, G. A. Petersson, H. Nakatsuji, X. Li, M. Caricato, A. V. Marenich, J. Bloino, B. G. Janesko, R. Gomperts, B. Mennucci, H. P. Hratchian, J. V. Ortiz, A. F. Izmaylov, J. L. Sonnenberg, D. Williams-Young, F. Ding, F. Lipparini, F. Egidi, J. Goings, B. Peng, A. Petrone, T. Henderson, D. Ranasinghe, V. G. Zakrzewski, J. Gao, N. Rega, G. Zheng, W. Liang, M. Hada, M. Ehara, K. Toyota, R. Fukuda, J. Hasegawa, M. Ishida, T. Nakajima, Y. Honda, O. Kitao, H. Nakai, T. Vreven, K. Throssell, J. A. Montgomery, Jr., J. E. Peralta, F. Ogliaro, M. J. Bearpark, J. J. Heyd, E. N. Brothers, K. N. Kudin, V. N. Staroverov, T. A. Keith, R. Kobayashi, J. Normand, K. Raghavachari, A. P. Rendell, J. C. Burant, S. S. Iyengar, J. Tomasi, M. Cossi, J. M. Millam, M. Klene, C. Adamo, R. Cammi, J. W. Ochterski, R. L. Martin, K. Morokuma, O. Farkas, J. B. Foresman, and D. J. Fox, Gaussian, Inc., Wallingford CT, 2019.

(17) Chai, J.-D.; Head-Gordon, M. Long-Range Corrected Hybrid Density Functionals with Damped Atom–Atom Dispersion Corrections. *Physical Chemistry Chemical Physics* **2008**, *10* (44), 6615. https://doi.org/10.1039/b810189b.

(18) Weigend, F.; Ahlrichs, R. Balanced Basis Sets of Split Valence, Triple Zeta Valence and Quadruple Zeta Valence Quality for H to Rn: Design and Assessment of Accuracy. *Physical Chemistry Chemical Physics* **2005**, *7* (18), 3297. https://doi.org/10.1039/b508541a.

(19) Marenich, A. V.; Cramer, C. J.; Truhlar, D. G. Universal Solvation Model Based on Solute Electron Density and on a Continuum Model of the Solvent Defined by the Bulk Dielectric Constant and Atomic Surface Tensions. *J Phys Chem B* **2009**, *113* (18), 6378–6396. https://doi.org/10.1021/jp810292n.

(20) Ochterski, J. W. *Vibrational Analysis in Gaussian*. **1999**, https://gaussian.com/vib/ accessed 20-08-2024 (11:49).

(21) Duisenberg, A. J. M.; Kroon-Batenburg, L. M. J.; Schreurs, A. M. M. An Intensity Evaluation Method: *EVAL* -14. *J Appl Crystallogr* **2003**, *36* (2), 220–229. https://doi.org/10.1107/S0021889802022628.

(22) Krause, L.; Herbst-Irmer, R.; Sheldrick, G. M.; Stalke, D. Comparison of Silver and Molybdenum Microfocus X-Ray Sources for Single-Crystal Structure Determination. *J Appl Crystallogr* **2015**, *48* (1), 3–10. https://doi.org/10.1107/S1600576714022985.

(23) Sheldrick, G. M. *SHELXT* – Integrated Space-Group and Crystal-Structure Determination. *Acta Crystallogr A Found Adv* **2015**, *71* (1), 3–8. https://doi.org/10.1107/S2053273314026370.

(24) Sheldrick, G. M. Crystal Structure Refinement with *SHELXL*. *Acta Crystallogr C Struct Chem* **2015**, *71* (1), 3–8. https://doi.org/10.1107/S2053229614024218.

(25) Spek, A. L. Structure Validation in Chemical Crystallography. *Acta Crystallogr D Biol Crystallogr* **2009**, *65* (2), 148–155. https://doi.org/10.1107/S090744490804362X.
